# Identification of abemaciclib derivatives targeting cyclin-dependent kinase 4 and 6 using molecular dynamics, binding free energy calculation, synthesis, and pharmacological evaluation

**DOI:** 10.3389/fphar.2023.1154654

**Published:** 2023-05-10

**Authors:** Yanting Zhou, Xiandeng Li, Peifang Luo, Huiting Chen, Yan Zhou, Xueting Zheng, Yuan Yin, Haoche Wei, Hongji Liu, Wen Xia, Mingsong Shi, Xiaoan Li

**Affiliations:** ^1^ Key Laboratory of Basic Pharmacology of Ministry of Education and Joint International Research Laboratory of Ethnocentric of Ministry of Education, Zunyi Medical University, Zunyi, Guizhou, China; ^2^ College of Pharmacy, Chongqing Medical University, Chongqing, China; ^3^ Department of Cardiovascular Surgery, Affiliated Hospital of Zunyi Medical University, Zunyi, Guizhou, China; ^4^ NHC Key Laboratory of Nuclear Technology Medical Transformation, Mianyang Central Hospital, School of Medicine, University of Electronic Science and Technology of China, Mianyang, Sichuan, China; ^5^ State Key Laboratory of Biotherapy/Collaborative Innovation Center of Biotherapy and Cancer Center, West China Hospital, Sichuan University, Chengdu, Sichuan, China; ^6^ Department of Ophthalmology, Mianyang Central Hospital, School of Medicine, University of Electronic Science and Technology of China, Mianyang, Sichuan, China

**Keywords:** abemaciclib, cyclin-dependent kinase 4, cyclin-dependent kinase 6, inhibitor, molecular dynamics simulation, binding free energy

## Abstract

CDK4/6 plays a crucial role in various cancers and is an effective anticancer drug target. However, the gap between clinical requirements and approved CDK4/6 drugs is unresolved. Thus, there is an urgent need to develop selective and oral CDK4/6 inhibitors, particularly for monotherapy. Here, we studied the interaction between abemaciclib and human CDK6 using molecular dynamics simulations, binding free energy calculations, and energy decomposition. V101 and H100 formed stable hydrogen bonds with the amine-pyrimidine group, and K43 interacted with the imidazole ring via an unstable hydrogen bond. Meanwhile, I19, V27, A41, and L152 interacted with abemaciclib through π-alkyl interactions. Based on the binding model, abemaciclib was divided into four regions. With one region modification, 43 compounds were designed and evaluated using molecular docking. From each region, three favorable groups were selected and combined with each other to obtain 81 compounds. Among them, C2231-A, which was obtained by removing the methylene group from C2231, showed better inhibition than C2231. Kinase profiling revealed that C2231-A showed inhibitory activity similar to that of abemaciclib; additionally, C2231-A inhibited the growth of MDA-MB-231 cells to a greater extent than did abemaciclib. Based on molecular dynamics simulation, C2231-A was identified as a promising candidate compound with considerable inhibitory effects on human breast cancer cell lines.

## 1 Introduction

**FIGURE 1:**
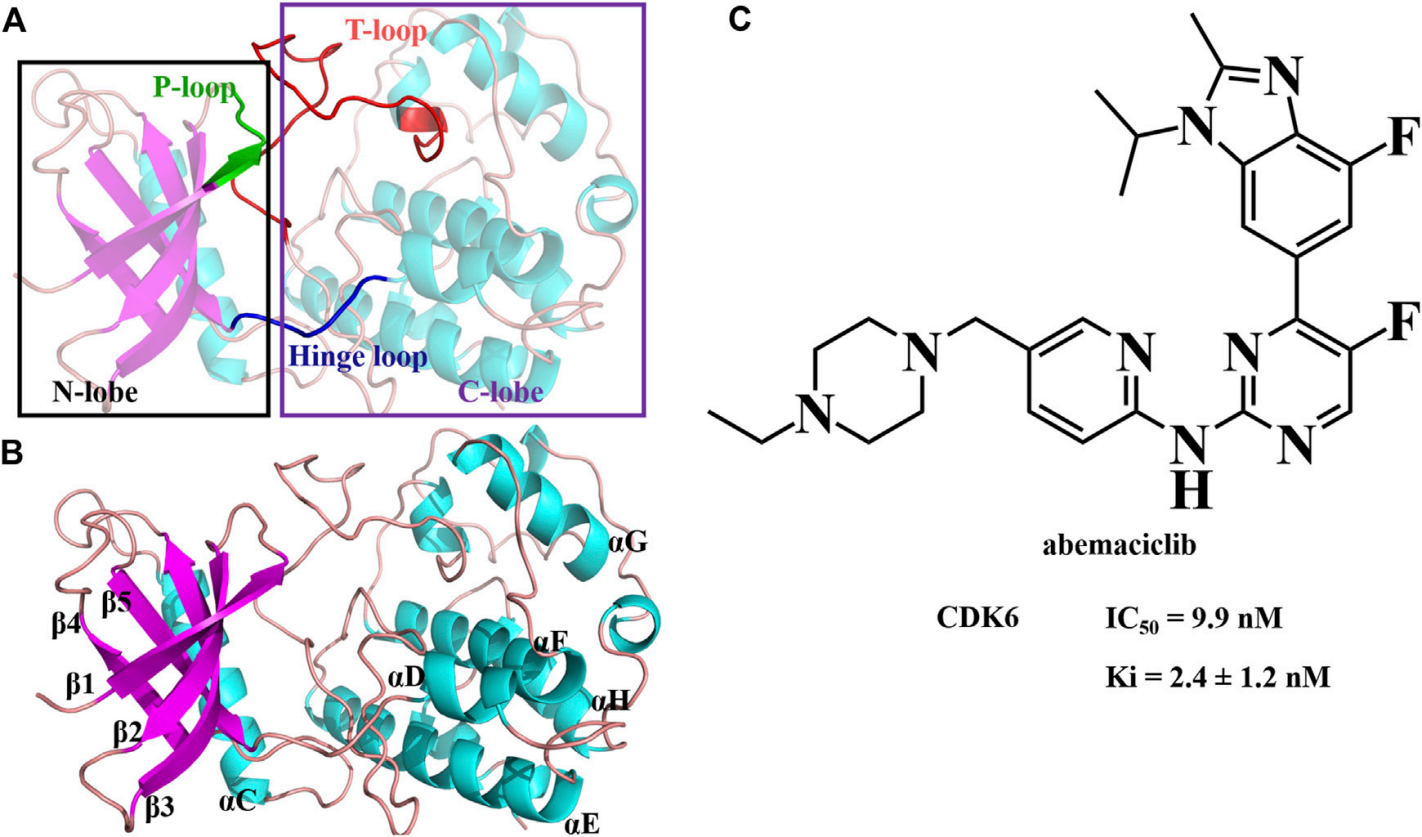
Structure of human CDK6 and abemaciclib. **(A)** Structure of human CDK6 labeled as different domains. **(B)** Different regions labeled with human CDK6. **(C)** Structure of abemaciclib, and biochemical activity of abemaciclib with human CDK6.

Cyclin-dependent kinases (CDKs) belong to the serine/threonine protein kinase family and have a classical protein kinase structure (Figure 1). They are activated by cyclin binding to form cyclin-CDK heterodimers. The cyclin-CDK heterodimer regulates cell cycle progression (Yousuf et al., 2022; Zabihi et al., 2022; Hope et al., 2023) and phosphorylates the upstream signals, including the retinoblastoma (Rb) protein (Macaluso et al., 2006; Choi and Anders, 2014; Spring et al., 2020). The phosphorylated Rb induces the release of the critical transcription factor early 2 factor (E2F) to initiate the transcription of target genes, thereby facilitating the activation of various signaling pathways, including the G1/S transition, mitochondrial dynamics, and metabolism (Sanchez-Martinez et al., 2015; Ingham and Schwartz, 2017; Matson and Cook, 2017; Sanchez-Martinez et al., 2019). When CDK4/6 is inhibited, the G1 phase of proliferating cells is blocked (Yuan et al., 2021; Chen et al., 2022; Watt and Goel, 2022; Yousuf et al., 2022). Additionally, CDK4/6 is disordered in numerous cancers (Wesierska-Gadek et al., 2009; Sanchez-Martinez et al., 2019; Yuan et al., 2021; Ghafouri-Fard et al., 2022; Kargbo, 2022; Yousuf et al., 2022; Zabihi et al., 2022), such as breast cancer (Gelbert et al., 2014; Chen et al., 2022; Watt and Goel, 2022), osteosarcoma (Wang and Bao, 2022), and acute megakaryoblastic leukemia (Qi et al., 2022). Thus, CDK4/6 is an effective anticancer drug target, especially in hormone receptor-positive and human epidermal growth factor receptor 2-negative (HR+/HER2-) breast cancer.

Several CDK4/6 inhibitors have been recently developed and employed to treat cancer (Supplementary Figure S1) (Bhurta and Bharate, 2022; Shi Z. F. et al., 2022; Ettl et al., 2022; Ji et al., 2022; Jorda et al., 2022; Manohar, 2022; Watt and Goel, 2022; Yousuf et al., 2022; Yu et al., 2022; Zeng et al., 2022). Three adenosine triphosphate (ATP)-competitive CDK4/6 inhibitors have been approved by the U.S. Food and Drug Administration (FDA); these are palbociclib (PD-0332991), ribociclib (LEE011), and abemaciclib (LY2835219) (Dhillon, 2015; Kim, 2017; Syed, 2017). Palbociclib is an oral, reversible, and selective CDK4/6 inhibitor with inhibition activity (Ki) of 0.26 ± 0.03 nM (CDK4) and 0.26 ± 0.07 nM (CDK6) (Chen et al., 2016). The FDA approved palbociclib in combination with letrozole as the first-line treatment for advanced breast cancer (Dhillon, 2015). Meanwhile, the selective CDK4/6 inhibitor ribociclib inhibits the activity, with Ki of 0.53 ± 0.08 nM and 2.3 ± 0.3 nM for CDK4 and CDK6, respectively (Chen et al., 2016). Furthermore, the combination of ribociclib and an aromatase inhibitor was approved as a treatment for advanced breast cancer in the USA in March 2017 (Syed, 2017). Conversely, abemaciclib was approved as a combination with fulvestrant or monotherapy (Kim, 2017). Abemaciclib is the only CDK4/6 inhibitor approved for breast cancer treatment as monotherapy (Abraham, 2018). Nevertheless, these three inhibitors have several side effects, such as neutropenia, gastrointestinal toxicities, anemia, and thrombopenia (Syed, 2017; Braal et al., 2021). The reasons for dose modification were myelosuppression (ribociclib and palbociclib) and diarrhea (abemaciclib). Therefore, developing novel CDK6 inhibitors is necessary. Additionally, there is a gap between the clinical requirements and approved CDK4/6 drugs. Accordingly, there is an urgent need to develop selective and oral CDK4/6 inhibitors, particularly for monotherapy.

N-{5-[(4-ethylpiperazin-1-yl) methyl] pyridin-2-yl}-5-fluoro-4-[4-fluoro-1-isopropyl-2-methyl-1H-benzo (d) imidazol-6-yl] pyrimidin-2-amine (abemaciclib; LY2835219; Verzenio; Figure 1) is a selective, administered, ATP-competitive, and reversible kinase inhibitor of CDK4/6 (Gelbert et al., 2014). Abemaciclib has several distinct chemical [2-anilino-2, 4-pyrimidine-(5-benzimidazole) scaffold], pharmacological (continuous dosing schedule), and clinical properties (single agent therapy) compared to palbociclib and ribociclib (Yuan et al., 2021; Wander et al., 2022). Abemaciclib inhibits Rb phosphorylation and induces G1 cell cycle arrest, resulting in antitumor activity (Gelbert et al., 2014; Wright and Md, 2021; Nardone et al., 2022; Nawa et al., 2022; Sun et al., 2022; Wander et al., 2022; Wang and Bao, 2022). Abemaciclib is a mainstay of HR + breast cancer treatment. It is currently under clinical trials for several other tumors, such as non-small cell lung cancer, Ewing sarcoma, and renal cell carcinoma (Kim, 2017; Kosovec et al., 2017; Small et al., 2017; Dowless et al., 2018; Naz et al., 2018; Wander et al., 2022; Wang and Bao, 2022). Meanwhile, the adverse effects of abemaciclib mainly include neutropenia, leukopenia, anemia, diarrhea, and eosinophilic pneumonia (Xie Q. et al., 2022; Lim et al., 2022; Mitarai et al., 2022). Notably, a black-box warning for abemaciclib resulted from venous thromboembolism (Watson et al., 2022). *In vitro*, abemaciclib inhibits CDK6 functions with Ki of 2.4 ± 1.2 nM (or 0.07 ± 0.01 nM or 8.2 ± 1.1 nM) and half-maximal inhibitory concentration (IC_50_) of 9.9 nM (Gelbert et al., 2014; Chen et al., 2016; Torres-Guzman et al., 2017). Abemaciclib potently inhibits osteosarcoma cell lines, with IC_50_ ranging from 90 nM to >20 μM (Wang and Bao, 2022). Some other potential targets have been identified from kinase screens *in vitro*, specifically CDK9, proviral insertion in murine malignancies serine/threonine kinases (PIM1), glycogen synthase kinase-3 beta (GSK3β), homeodomain-interacting protein kinase 2 (HIPK2), dual-specificity tyrosine-phosphorylation-regulated kinase 2 (DYRK2), and casein kinase 2 (CK2) with 57 ± 42 nM, 50 nM, 192 nM, 31 nM, 61 nM, and 117 nM, respectively (Gelbert et al., 2014; Kaltheuner et al., 2021). These studies indicate that abemaciclib has remarkable potential for use in developing novel inhibitors with improved inhibition activity and selectivity.

In this study, we describe a binding model and the key interactions between abemaciclib and human CDK6. The binding model of abemaciclib with human CDK6 was optimized using molecular dynamics (MD) simulations. The binding free energy was calculated using the molecular mechanics/generalized Born (GB) surface area (MM/GBSA) method and decomposed to identify the key residues that mainly contribute to the binding of abemaciclib with CDK6. Based on the binding models, some abemaciclib derivatives were designed, and their binding affinities were tested using molecular docking. Candidate inhibitors were synthesized, and their inhibitory activity against CDK6 was evaluated *in vitro* using MDA-MB-231 and MCF-7 cell lines.

## 2 Materials and methods

### 2.1 Molecular dynamics simulations

The abemaciclib/CDK6 complex structure with a resolution of 2.27 Å was obtained from the Protein Database Bank (PDB ID: 5L2S) (Chen et al., 2016). The restrained electrostatic potential (RESP) protocol (Bayly et al., 1993) was employed to generate the atom charge of abemaciclib. The force field parameters were produced based on the general amber force field (GAFF, version 2) (Wang et al., 2004) in AMBERTools21 (Case et al., 2020). The AMBER ff19SB force field (Tian et al., 2020) was applied to construct the topology parameters of human CDK6. Afterward, the abemaciclib/CDK6 system was neutralized by adding sodium chloride ions and solvated in a cuboid box with TIP3P water (Jorgensen et al., 1983). Finally, the solvated abemaciclib/CDK6 complex system included human CDK6 (291 residues), one small molecule (abemaciclib), and solvent water (19,723). The complex was subjected to the steepest descent method (9,000 steps) and conjugate gradient (1,000 steps), while abemaciclib and human CDK6 were fixed. Next, the entire abemaciclib/CDK6 system was optimized using the 10,000-step conjugate gradient method. Subsequently, Langevin dynamics (Martyna et al., 1994; Feller et al., 1995) was performed to increase the temperature of the solvated abemaciclib/CDK6 system from 0 K to 300 K for 200 ps. Subsequently, isotropic position scaling (Berendsen et al., 1984) was employed to maintain the pressure at 1 bar and perform a 200 ps simulation. Then, the abemaciclib/CDK6 system was equilibrated with an isothermal-isobaric ensemble at 300 K and 1 bar, and then a 200 ps simulation was run. Finally, the solvated abemaciclib/CDK6 system was subjected to 500 ns MD simulations to collect and analyze data for abemaciclib binding with human CDK6. In this study, MD simulations were performed using the AMBER20 software (Case et al., 2020). To analyze the trajectories of abemaciclib/CDK6, the *CPPTRAJ* module (Roe and Cheatham, 2018; Cheatham et al., 2019) was employed for calculating the root mean square deviation (RMSD), root-mean-square fluctuation, hydrogen bonds, distance, and angle. Detailed information regarding MD simulations and cluster analysis is provided in the Supplementary Material.

### 2.2 Binding free energy calculation

The MM/GBSA approach has been widely used to evaluate ligand and enzyme systems; it was used to calculate the binding free energies between abemaciclib and human CDK6 (Srinivasan et al., 1998; Lee et al., 2004; Tse and Verkhivker, 2015; Wang et al., 2017; Shi and Xu, 2019). The framework of MM/GBSA has been extensively discussed (Honig and Nicholls, 1995; Genheden and Ryde, 2015; Onufriev and Case, 2019). Different energy terms of abemaciclib/CDK6 complex system were obtained via a statistical average from the last 100 ns of the MD trajectory over 1,000 frames. The entropy was averaged over 100 frames. The contribution of each residue was obtained by decomposing the enthalpy energy between human CDK6 and abemaciclib with MM/GBSA energy decomposition (Gaillard and Simonson, 2014). The energy terms were assessed using the MMPBA.py program in AMBERTools21 (Miller et al., 2012). Additional information about the calculation of binding free energy is provided in the Supplementary Material.

### 2.3 Chemistry

**SCHEME 1:**
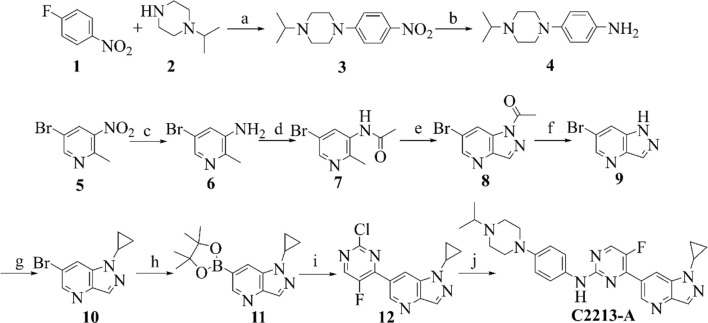
Synthetic route of compound C2213-A. Reagents and conditions: **(A)** K_2_CO_3_, MeCN, 80°C; **(B)** Pd/C, H_2_, rt; **(C)** Fe/NH_4_Cl, MeOH/H_2_O, 80°C; **(D)** AcOK, acetic anhydride, CHCl_3,_ reflux; **(E)** isopentyl nitrite, 18-Crown-6, K_2_CO_3_, CHCl_3,_ reflux; **(F)** KOH, H_2_O, rt; **(G)** cyclopropylboronic acid, Na_2_CO_3_, Cu (AcO)_2_, 2,2′-Bipyridine, DCM, 80°C; **(H)** bis (pinacolato) diboron, PdCl_2_ (dppf), AcOK, dioxane, 90°C; **(I)** 2,4-Dichloro-5-fluoropyrimidine, PdCl_2_ (dppf), K_2_CO_3_, dioxane/water (V/V, 10/1), 80°C; **(J)** compound 4, Pd_2_ (dba)_3_, Xantphos, Cs_2_CO_3_, dioxane, reflux.

Compounds 1 and 2 underwent N coupling reactions in the presence of potassium carbonate to generate compound 3 (Scheme 1). Intermediate 3 was reduced to 4 in a hydrogen atmosphere under palladium catalysis. Compound 5 was reduced to compound 6 under the action of iron powder and ammonium chloride. Then, subsequent amide condensation of 6 with acetic anhydride afforded compound 7. Intermediate 7 underwent cyclization and hydrolysis to yield compounds 8 and 9 successively. Intermediate 9 and isopropylboronic acid underwent a C-N coupling reaction to generate compound 10. Intermediate 10 then underwent a Suzuki coupling reaction to generate compound 12. Finally, intermediates 12 and 4 underwent Buchwald coupling to yield the target product C2213-A. Detailed chemical information is provided in the Supplementary Material.

### 2.4 Pharmacological evaluation

The inhibitory activity of C2213-A against CDK6 was validated using the KinaseProfiler radiometric protein kinase assay and IC_50_ values, which were obtained from Eurofins Pharma Discovery Services UK Limited (Wolverhampton, UK). The ATP concentrations used here represent the *K*_m_ values of human CDK6. Cell proliferation assays for MDA-MB-231 and MCF-7 cells, colony formation assays for MDA-MB-231 cells, and cell cycle analysis based on MDA-MB-231 cells are described in the Supplementary Material.

## 3 Results and discussion

### 3.1 System stability

**FIGURE 2:**
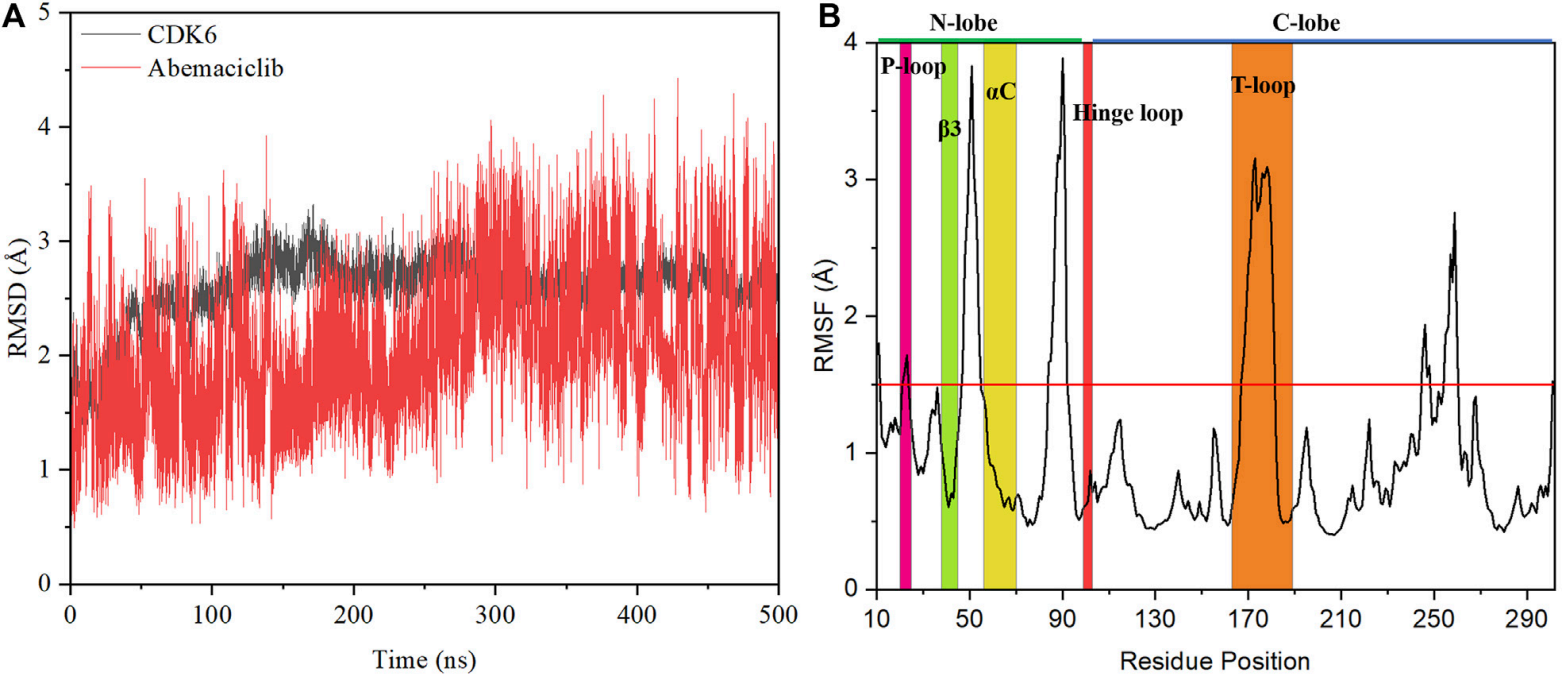
Stability analysis of the abemaciclib/CDK6 system. **(A)** Root mean square deviation (RMSD) value of heavy backbone atoms for human CDK6 and that of heavy atoms of abemaciclib along 500 ns molecular dynamics (MD) simulation for the abemaciclib/CDK6 system. **(B)** Root-mean-square fluctuation variations for Cα atom of human CDK6 for the abemaciclib/CDK6 system from the 500 ns MD simulation.

The stability of the abemaciclib/CDK6 complex system was determined from the RMSD values of CDK6 and abemaciclib. The structure of the receptor protein CDK6 was stable after 100 ns (Figure 2). However, the conformation of abemaciclib fluctuated during the simulation. The average RMSD value of CDK6 (2.57 ± 0.28 Å) was larger than that of abemaciclib (2.03 ± 0.59 Å) for the abemaciclib/CDK6 complex system. Meanwhile, the RMSD value of CDK6 was centered at 2.57 Å and that of abemaciclib was between 1 and 4 Å (Supplementary Figure S3). This result suggested that the fluctuation in the conformation of abemaciclib was greater than that in CDK6, which is more stable than abemaciclib. However, the RMSD value was increased to 3.33 ± 0.43 Å when abemaciclib was not used for human CDK6 (Supplementary Figure S4). This stable CDK6 and fluctuating abemaciclib conformation can also be found in the other two replicated abemaciclib/CDK6 complex systems (Supplementary Figure S5). In addition, the RMSD value of the C-lobe region (residues: 104–300, 3.18 ± 0.41 Å) was larger than that of the N-lobe region (residues: 11–98, 2.21 ± 0.35 Å), as shown in Supplementary Figure S6. The structure of human CDK6 was stable in the simulations after 100 ns, whereas that of abemaciclib was unstable throughout the simulation. The stability of this system suggests that CDK6 with abemaciclib is more stable than apo-CDK6, and abemaciclib conformation fluctuates in the ATP-binding pocket of human CDK6.

The root-mean-square fluctuation in the conformation of human CDK6 residues was analyzed based on the frames in the 500 ns simulations. Notably, there were four regions of human CDK6 with significant fluctuations (Figure 2)—the regions at positions 47–55 (loop region between β3 and αC), 84–92 (loop region between β4 and β5), 167–181 (T-loop), and 245–260 (loop region between αG and αH). The T-loop is in an open conformation from the crystal structures of human CDK6, such as fisetin (PDB ID: 1XO2) (Lu et al., 2005), palbociclib (PDB ID: 2EUF) (Lu and Schulze-Gahmen, 2006), aminopurvalanol (PDB ID: 2F2C) (Lu and Schulze-Gahmen, 2006), and pyrido-[4′, 3′: 4, 5] pyrrolo [2,3-d] pyrimidine compound (PDB ID: 4TTH) (Li et al., 2014) binding with human CDK6 (Supplementary Figure S7). Meanwhile, some published crystal structures, such as 4-(pyrazol-4-yl)-pyrimidines (PDB ID:3NUX and 3NUP) (Cho et al., 2010), 7-azabenzimidazoles (PDB ID:4AUA and 4EZ5) (Cho et al., 2012), palbociclib (PDB ID: 5L2I) (Chen et al., 2016), abemaciclib (PDB ID: 5L2S) (Chen et al., 2016), ribociclib (PDB ID: 5L2T) (Chen et al., 2016), pyrazolopyrimidine compounds (PDB ID: 6OQL and 6OQO) (Bronner et al., 2019), and human CDK6, were missing from the T-loop. In addition, the T-loop for protein kinase has diversified conformations, with open, closed, and intermediate conformations. For instance, human FAK may exhibit an open or closed T-loop conformation while binding with FAK inhibitors (Shi et al., 2022a; Guo et al., 2022). However, some inhibitors, such as the pan-kinase inhibitor bosutinib-bound SIK2, can also bind to the protein kinase with conformational plasticity in the active pocket of the T-loop (Shi et al., 2022c). Thus, the large fluctuation in the conformation of the T-loop of CDK6 binding with abemaciclib is a classical phenomenon. Additionally, the T-loop of CDK6 is phosphorylated to activate kinase activity and regulate downstream signaling.

These observations indicate that different conformations of the T-loop are extremely important for studying the interactions between abemaciclib and human CDK6. The loop region (residues 47–55) between β3 and αC was also found to be flexible. This region directly or indirectly regulates the salt bridge between K43 in β3 and E61 in αC; it also affects the kinase activity of CDK6. From the crystal inhibitor/human CDK6 complex structures, the salt bridge can be formed with fisetin, palbociclib, and aminopurvalanol compounds binding to human CDK6 (Supplementary Figure S8). Meanwhile, a salt bridge cannot form when palbociclib, abemaciclib, and ribociclib bind to human CDK6. Thus, the flexibility of this loop region suggested that the conformation of αC fluctuated during the simulation. The RMSD value of αC was calculated after the superposition of the entire human CDK6 structure, indicating that the conformation of αC changed during the simulation time (Supplementary Figure S9). The distance between K43 and E61 was also determined; it was 11.51 ± 1.22 Å and 11.46 ± 1.18 Å for K43_NZ−E61_OE1 and K43_NZ−E61_OE2, respectively (Supplementary Figure S10). This result implied that the salt bridge was not formed in the simulation, consistent with the experimental results (9.88 Å and 10.82 Å for K43_NZ−E61_OE1 and K43_NZ−E61_OE2, respectively) (Chen et al., 2016). The flexible region (residues: 84–92) was located between β4 and β5 in the N-lobe of the kinase domain, far from the ATP-binding site. The last flexible region in the C-lobe was also far from the ATP-binding pocket, which may not directly affect inhibitor binding with CDK6. These flexible regions, especially the loop region (between β3 and αC) and the T-loop, can be induced by the binding of abemaciclib to the ATP-binding site of human CDK6. Furthermore, the overall structure of CDK6 was stable near the T-loop region in the simulation.

Snapshots at 100 ns, 200 ns, 300 ns, 400 ns, and 500 ns, obtained from the MD simulation, were drawn to demonstrate the conformational change in abemaciclib in the ATP-binding site of human CDK6 (Supplementary Figure S11). However, abemaciclib remained as a stable binding complex during the simulation period, which indicated that abemaciclib can directly bind to the ATP-binding pocket of human CDK6. The piperazine group of abemaciclib, which was exposed to the solvent, fluctuated during the simulation. This result also suggests that the piperazine group of abemaciclib can be replaced with other solvent-friendly groups, such as piperidine, morpholine, cyclohexane, and cyclopentane. The overall structure of abemaciclib fluctuated in the ATP-binding site, which was consistent with the fluctuation in the RMSD value of abemaciclib. This surface-exposed piperazine group of abemaciclib can be considered as a modification.

### 3.2 Hydrogen bond analysis

Hydrogen bonds play an important role as kinase inhibitors bound to protein kinases and are commonly required for the actin of potential kinase inhibitors (Stavenger, 2008; Xing et al., 2015; Pang et al., 2021). Moreover, hydrogen bonds may significantly contribute to the binding of inhibitors with human CDK6 as a protein kinase (Xie Z. L. et al., 2022). Accordingly, the number of hydrogen bonds formed between abemaciclib and human CDK6 was first examined for the abemaciclib/CDK6 complex system from the simulation trajectories (Supplementary Figure S12). The wide range of hydrogen bond numbers (0–7) for the abemaciclib/CDK6 system indicated that some unstable hydrogen bonds were formed during the simulation. The number of hydrogen bonds formed between human CDK6 and abemaciclib was 2.59 ± 0.86. Hydrogen bonds were mainly occupied by 2 or 3 number, indicating that there are disordered hydrogen bonds between abemaciclib and the ATP-binding site of human CDK6.

**FIGURE 3:**
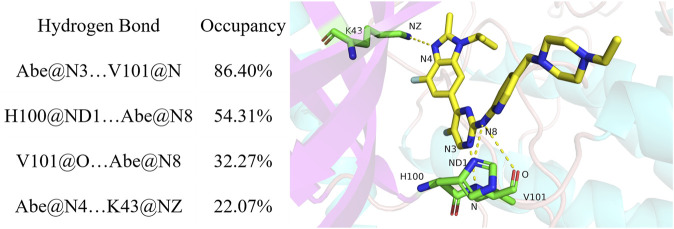
Hydrogen analysis between abemaciclib and human CDK6.

Hydrogen bond occupancy analysis was performed to determine stable hydrogen bonds between abemaciclib and CDK6. In this study, a hydrogen bond was formed between the acceptor and donor atoms <3.5 Å, and the internal angle of the acceptor···H-donor was >120°. The results of hydrogen bond occupancy analysis are summarized in Figure 3. A high occupancy was observed for abemaciclib with the hinge loop of human CDK6. The hydrogen bond occupancy between atom N of V101 and atom N3 of abemaciclib was 86.40%, indicating that this hydrogen bond can be stably formed during the simulation. The other hydrogen bond formed between abemaciclib and the hinge loop of CDK6 was transformed between the ND1 atom of H100 (54.31%) and the O atom of V101 (32.27%) to form a hydrogen bond with N8 of abemaciclib. However, in the last 200 ns of the simulation, hydrogen bonds were mainly formed with H100 for 80.27% occupancy (Supplementary Figure S13). The formation of these two hydrogen bonds, which formed between abemaciclib and the hinge loop of CDK6, was consistent with the results of experimental and theoretical studies (Yuan et al., 2021; Chen et al., 2022; Sokolsky et al., 2022; Wang et al., 2023). In addition, the hydrogen bond between the atom N4 of abemaciclib and the NZ atom of K43 was not unstable from the occupancy with 22.07% for the 500 ns simulation and 20.74% for the last 200 ns simulation, respectively. Therefore, two stable hydrogen bonds were formed between abemaciclib and the hinge loop of CDK6.

Occupancy was expressed as a percentage of the period (500 ns, 50,000 frames) during which specific hydrogen bonds were formed. A hydrogen bond is defined as the distance between the acceptor and donor atoms (<3.5 Å), with an internal angle between the H-acceptor, hydrogen atom, and H-donor (>120°).

The distance between the acceptor and donor atoms for the hydrogen bond and the angles between the acceptor, hydrogen, and donor atoms were also determined (Supplementary Figures S14, S15). The distances for Abe@N3 … V101@N (abemaciclib was labeled as Abe), H100@ND1 … Abe@N8, V101@O … Abe@N8, and Abe@N4 … K43@NZ were 3.12 ± 0.18 Å, 4.01 ± 1.10 Å, 3.69 ± 0.53 Å, and 3.21 ± 0.32 Å from the 500 ns simulation, respectively. Meanwhile, those distance were 3.13 ± 0.18 Å, 3.32 ± 0.35 Å, 3.94 ± 0.42 Å, and 3.19 ± 0.25 Å for Abe@N3 … V101@N, H100@ND1 … Abe@N8, V101@O … Abe@N8, and Abe@N4 … K43@NZ in the last 200 ns simulation, respectively. This result indicates that the hydrogen bond between the N3 of V101 and the amine-pyrimidine group has the same strength as that between ND1 of H100 or the O of V101 and the amine-pyrimidine group. These results were consistent with those of the occupancy analysis. Meanwhile, the distance between ND1 of H100 and the amine-pyrimidine group of abemaciclib (3.32 ± 0.35 Å) was maintained to form a hydrogen bond. They also indicate that this hydrogen bond is stable during the last 200 ns of the simulation. However, these two hydrogen bonds should also be maintained when designing novel CDK6 inhibitors. Thus, other scaffolds, which also formed hydrogen bonds with the hinge loop, such as 1H-pyrrolo [2,3-b]pyridine, 1H-indazole, adenine, 7H-pyrrolo [2,3-d] pyrimidin-4-amine, 1H-pyrazole-3-amine, 7H-pyrrolo [2,3-d] pyrimidine pyrimidine-4, 6-diamine, and pyridine, can be replaced with the 2-amine-pyrimidine group.

### 3.3 Binding free energy

The information regarding the binding of abemaciclib to CDK6 was obtained from simulations. However, determining the binding affinity of abemaciclib and CDK6 is challenging. Thus, the binding free energy for abemaciclib and CDK6 was calculated using the MM/GBSA method as a quantitative measurement. This study used frames from the last 100 ns to calculate the binding free energy. Detailed information regarding the binding free energies of the abemaciclib/CDK6 system is summarized in Supplementary Table S1. Abemaciclib bound to the ATP-binding pocket of human CDK6, with $∆G_{bind}^{cal}$ = −9.74 kcal/mol (Table 1), which is consistent with the experimental binding free energy of −9.6 kcal/mol obtained from isothermal titration calorimetry without cyclin D (Chen et al., 2016). Similarly, the binding free energies for replicas 1 and 2 were −8.99 and −12.26 kcal/mol, respectively (Supplementary Tables S2, S3). Due to the similarity in results among the three simulations, the first simulation was employed for analysis in the following section. The binding entropy and enthalpy for abemaciclib and human CDK6 were −24.18 kcal/mol and −33.92 kcal/mol, respectively. The negative values of enthalpy and entropy suggest that the binding of abemaciclib to CDK6 is an enthalpy-driven process.

**TABLE 1:** Binding free energies, decomposition, electrostatic interactions ( $E_{ele}$ ), van der Waals interactions ( $E_{vdW}$ ), solvation free energies ( $E_{GB}$ ), nonpolar solvation energies ( $E_{surf}$ ), and entropy ( $TS_{total}$ ) of the abemaciclib/CDK6.

Energy	Complex	Receptor	Ligand	Delta
$E_{vdW}$	−2,396.80	−2,339.28	−5.83	−51.70
$E_{ele}$	−19,936.00	−20,063.71	155.02	−27.31
$E_{GB}$	−3,259.72	−3,291.36	−19.65	51.29
$E_{surf}$	104.35	106.18	4.37	−6.20
$E_{gas}$	−6,224.90	−5,990.32	−155.57	−79.01
$E_{solv}$	−3,155.37	−3,185.18	−15.28	45.09
$E_{gas}+E_{sol}$	−9,380.27	−9,175.50	−170.84	−33.92
$TS_{total}$	3,282.60	3,241.42	65.36	−24.18
$∆G_{bind}^{cal}$				−9.74
$∆G_{bind}^{\exp}$				−9.60

The energy values are expressed in kcal/mol. $E_{gas}$ : contribution to the binding free energy, $E_{vdW}$ + $E_{ele}$ . $E_{solv}$ : contribution to the binding free energy, $E_{GB}$ + $E_{surf}$ . $∆G_{bind}^{cal}$ : final estimated binding free energy from $E_{gas}+G_{sol}$ — $TS_{total}$ . $∆G_{bind}^{\exp}$ was obtained from reference 31.

Van der Waals (vdW) interactions (−51.70 kcal/mol) mainly contributed to the binding between abemaciclib and CDK6. The electronic interaction (−27.31 kcal/mol) is also favorable for the binding process. vdW interactions contribute more energy (−24.39 kcal/mol) than electronic interactions for the binding of abemaciclib with CDK6. Generally, the binding energy is divided into two major categories—polar binding energy ( $E_{ele}$ + $E_{GB}$ ) and nonpolar binding energy ( $E_{vdW}$ + $E_{surf}$ ). The polar interactions with a positive value (23.98 kcal/mol) are disadvantageous for abemaciclib and CDK6. Conversely, the negative values of the nonpolar terms were −57.90 kcal/mol, suggesting that the nonpolar binding is the main contributor to the binding of abemaciclib with CDK6. Thus, polar interactions are disadvantageous and nonpolar interactions are advantageous for the binding of abemaciclib to CDK6. RMSD analysis showed that the conformation of abemaciclib in the ATP-binding site fluctuated. Then, cluster analysis for the 20,000 frames obtained from the last 200 ns simulation was performed to find the different class trajectories for the binding of abemaciclib with CDK6. Two class clusters of abemaciclib bound CDK6, with 1,030 and 1,176 frames for Clusters 1 and 2, respectively (Supplementary Figure S16). The binding free energy was also calculated from the trajectories extracted from Clusters 1 and 2. The binding free energy for Cluster 1 was −6.05 kcal/mol, which was less than the experimental result; this showed that this binding model was not the best model for the binding of abemaciclib with CDK6 (Supplementary Table S4). However, the binding affinity for Cluster 2 (−7.45 kcal/mol, Supplementary Table S5) was higher, by approximately 1.00 kcal/mol, than that of Cluster 1 and lower, by about 2.00 kcal/mol, than the experimental value (−9.60 kcal/mol). The energy difference between Cluster 1 or 2 and the experimental result was mainly obtained from the entropy term (2.78 and 1.89 kcal/mol for Clusters 1 and 2, respectively). In total, abemaciclib bound human CDK6 with a strong binding free energy.

### 3.4 Free energy decomposition

The binding of abemaciclib to human CDK6 has been explained by MD simulation. The key residues in the abemaciclib/CDK6 complex system remain unclear. Thus, the per-residue energy decomposition approach was employed to identify the key residues, which contributed more than 0.50 kcal/mol to the abemaciclib/CDK6 system (Supplementary Table S6).

H100 and V101 in the hinge loop of CDK6 contributed −1.17 and −0.91 kcal/mol from the hydrogen bonds formed with the amine-pyrimidine group of abemaciclib, which agreed with hydrogen bond analysis results (Table 2). H100 formed a stable hydrogen bond and contributed more than V101 did. Thus, the hydrogen bonds formed between the hinge loop of CDK6 and the amine-pyrimidine group of abemaciclib must remain for novel inhibitor binding with CDK6.

**TABLE 2:** Free energy decomposition for the abemaciclib/CDK6 complex on the individual residue basis.

Residue	$∆E_{vdW}$	$∆E_{ele}$	$∆E_{GB}$	$∆E_{surf}$	$∆E_{subtotal}$	$S∆E_{subtotal}$	$B∆E_{subtotal}$
I19	−3.67	−0.78	1.77	−0.60	−3.27	−3.59	0.31
V27	−1.70	−0.32	0.22	−0.15	−1.95	−1.78	−0.17
K29	−0.84	−1.71	2.10	−0.08	−0.53	−0.19	−0.34
A41	−1.35	−0.07	0.14	−0.13	−1.40	−1.36	−0.05
K43	−0.81	−7.62	5.36	−0.07	−3.14	−2.77	−0.37
V77	−0.54	0.02	−0.44	−0.03	−0.99	−0.70	−0.28
F98	−1.15	0.03	0.12	−0.08	−1.08	−0.87	−0.21
E99	−0.31	−0.23	2.29	−0.02	1.72	0.08	1.64
H100	−1.49	−1.65	2.08	−0.11	−1.17	0.05	−1.23
V101	−1.07	−0.69	0.90	−0.05	−0.91	−0.76	−0.15
D104	−0.67	−1.46	3.90	−0.13	1.64	1.50	0.14
N150	−0.74	0.45	−0.31	−0.05	−0.65	−0.52	−0.13
L152	−1.66	−0.07	−0.38	−0.25	−2.36	−2.24	−0.12
A162	−0.85	−0.44	0.28	−0.08	−1.09	−0.82	−0.27
D163	−2.02	−0.09	6.25	−0.20	3.94	3.89	0.05
R168	−0.34	0.17	−1.15	−0.01	−1.32	−1.31	−0.01

The total energy ( $∆E_{subtotal}$ ) decomposition was calculated in terms of the contributions of the van der Waals energy ( $∆E_{vdW}$ ), electrostatic interaction energy ( $∆E_{ele}$ ), polar solvation free energy ( $∆E_{GB}$ ), nonpolar solvation free energy ( $∆E_{surf}$ ), backbone energy ( $B∆E_{subtotal}$ ), and side chain energy ( $S∆E_{subtotal}$ ). The energy values are expressed in kcal/mol.

However, the largest contribution residue, K43 (−3.14 kcal/mol), forms an unstable hydrogen bond with benzo [d]imidazole group of abemaciclib. Meanwhile, K43 also formed a hydrogen bond with D163 (the distance between NZ of K43 and OD1 of D163 was 2.90 ± 0.30 Å, Supplementary Figure S17). This hydrogen bond between K43 and D163 reduced the strength of the hydrogen bond between abemaciclib and K43. Thus, the contribution of D163 (3.94 kcal/mol) was unfavorable for the binding of abemaciclib to human CDK6. Additionally, the OD2 of D163 formed a hydrogen bond with the ND2 of N150 (distance between the ND2 of N150 and the OD2 of D163 was 3.58 ± 0.57 Å). This weakened the hydrogen bond between D163 and K43 to contribute to the binding of abemaciclib with K43; N150 contributed −0.65 kcal/mol. The orientation of R168 must be considered to disturb the interactions among abemaciclib, D163, K43, and N150. For example, the distance between the NH2 of R168 and the OD2 of D163 was 14.49 ± 1.83 Å for the first 10 ns and 5.20 ± 0.32 Å for the last 10 ns. In the simulation, the location of D163 remained, and R168 was transformed into the ATP-binding site (Supplementary Figure S18) and contributed −1.32 kcal/mol. Meanwhile, the region (^163^DFGLAR^168^) at the beginning of the T-loop plays a key role in the kinase activity of CDK6. The conserved DFG motif also regulates the activity of other protein kinases, such as SIKs, FAK, and ROCKs (Roskoski, 2016; Bronner et al., 2019; Tesch et al., 2021; Arter et al., 2022; Shi et al., 2022a; Shi et al., 2022b; Ladduwahetty et al., 2022). Furthermore, the binding of palbociclib with human CDK6 showed that R168 was far from the ATP-binding site (Supplementary Figure S19) (Lu and Schulze-Gahmen, 2006; Chen et al., 2016). This orientation of R168 means that R168 can be employed to form an interaction with the other groups by substituting the benzo [d]imidazole group of abemaciclib.

The P-loop also plays an important role in the binding of inhibitors to protein kinases, such as TYK2 and ABL1 (Yang et al., 2014; Wang et al., 2017; Fensome et al., 2018). The I19 in P-loop region also contributes the largest energy (−3.27 kcal/mol) for the binding of abemaciclib with human CDK6. In particular, the side chain of I19 contributed −3.59 kcal/mol, and the backbone contributed 0.31 kcal/mol. The side chain of I19 interacted with the π-alkyl interaction of the pyridine ring of abemaciclib, which also resulted from the distance between the pyridine ring of abemaciclib and CD1 of I19 (3.96 ± 0.38 Å for the last 200 ns, Supplementary Figure S20). Experimental and computational studies have also shown that I19 plays a key role in the binding of other inhibitors with CDK6 (Chen et al., 2022; Yuan et al., 2022; Bhattacharya et al., 2023; Wang et al., 2023).

V27, located in β2, contributed approximately −1.95 kcal/mol to abemaciclib binding with CDK6; the side chain of V27 interacted with the imidazole ring of abemaciclib. The distances between the CG1/CG2 of V27 and the imidazole ring of abemaciclib were 4.36 ± 0.50 Å and 4.18 ± 0.34 Å, respectively (Supplementary Figure S21). The distance of the V27_CG1-imidazole ring decreased by approximately 170 ns. However, the distances for the V27-imidazole ring were well maintained in the last 300 ns of the simulation. This result indicated that the π-alkyl interaction for V27-abemaciclib was mainly attributed to the abemaciclib bound to human CDK6. In other words, the imidazole ring must balance the π-alkyl from V27 and the hydrogen bond from K43. Thus, the imidazole ring can be substituted by the 1, 2, 3-triazole ring.

The ATP-binding site is formed between the N-lobe and C-lobe for protein kinases. β3, which includes A41, is the main domain of the N-lobe that forms the ATP-binding pocket of human CDK6. A41 forms a π-alkyl interaction with the pyrimidine ring of abemaciclib. However, L152 of the C-lobe also interacted with the pyrimidine ring via π-alkyl interactions. A41 and L152 formed clips to stabilize the interaction between abemaciclib and CDK6. Those π-alkyl interactions can be verified from the distance between CB of A41 or CD1 of L152 and the pyrimidine ring (A41_CB-Abe_pyrimidine: 3.70 ± 0.26 Å and L152_CD1-Abe_pyrimidine: 3.49 ± 0.25 Å, Supplementary Figure S22). The angle between CB of A41, the pyrimidine ring of abemaciclib, and CD1 of L152 was approximately 154.96° ± 9.97°. This suggests that the three points were related to the line structure. These π-alkyl interactions resulted in A41 and L152 contributing to −1.40 and −2.36 kcal/mol for abemaciclib, forming a complex system with human CDK6. Hence, the pyrimidine ring needs to remain as an aromatic ring and maintain the hydrogen bond with the hinge loop of CDK6, such as 1H-pyrrolo [2, 3-b] pyridine, 1H-indazole, adenine, 7H-pyrrolo [2, 3-d] pyrimidin-4-amine, 1H-pyrazole-3-amine, 7H-pyrrolo [2, 3-d] pyrimidine pyrimidine-4,6-diamine, and pyridine.

Additionally, some hydrophobic residues possessed substantial subtotal binding free energies, such as V77, F98, E99, and A162. These residues form a hydrophobic cavity to accommodate fluorine on the pyrimidine ring of abemaciclib (Supplementary Figure S23). Meanwhile, the cavity was not large enough for large groups and only accommodated small groups, such as halogen, methyl, ethyl, propyl, and cyclopropyl groups.

The conformation of the piperazine ring of abemaciclib fluctuated during the simulation and was exposed to the solvent. This indicates that the piperazine group may interact with the N-lobe (K29) or C-lobe (D104) of human CDK6 (Supplementary Figure S24). K29 contributed to the binding of abemaciclib with CDK6 via the van der Waals interactions (−0.84 kcal/mol) and electrostatic interactions (−1.71 kcal/mol). Those interactions also formed with the side chain (−0.19 kcal/mol) and backbone (−0.37 kcal/mol) of K29. However, D104 was unfavorable (1.64 kcal/mol. These disadvantageous interactions were mainly obtained from polar solvation interaction (3.90 kcal/mol). In other words, if D104 forms a hydrogen bond with the piperazine group of abemaciclib, the solvated water around D104 will first repush, possibly increasing the energy barrier. Thus, water molecules around the ATP-binding site of human CDK6 should also be considered when designing novel CDK6 inhibitors.

The main contributions of H100 and V101 to human CDK6 were obtained from hydrogen bonds. K43, N150, D163, R168, and the imidazole ring of abemaciclib form a hydrogen bond network. I19, V27, K29, A41, V77, F98, L152, and A162 were favorable for abemaciclib binding with CDK6 while E99 and D104 were unfavorable. The ATP-binding pocket binds to the inhibitor mainly via hydrogen bonds and π-alkyl interactions.

### 3.5 Design of novel CDK6 inhibitors

**FIGURE 4:**
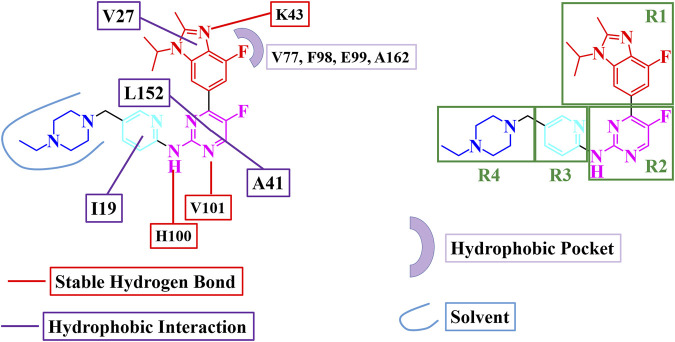
Binding model for abemaciclib binding with human CDK6.

Based on the MD simulation and binding free energy calculations, a binding model and key interactions for abemaciclib binding with human CDK6 were obtained (Figure 4). The benzo [d] imidazole ring of abemaciclib, defined as R1, formed hydrogen bonds with K43 and π-alkyl interactions with V27. V101 and H100 in the hinge loop formed two hydrogen bonds with amine-pyrimidine (named R2), constituting flips with A41 and L152. Meanwhile, the pyridine ring of abemaciclib, which interacted with I19 of CDK6 via π-alkyl interactions, was probed as R3. The piperazine group was also modified to R4 and exposed to the solvent.

Cluster analysis for the 20,000 frames obtained from the last 200 ns simulation was performed to find the representative frame for the binding of abemaciclib with CDK6. There were two representative clusters of abemaciclib binding CDK6 with 82.4% and 17.6% for Clusters 1 and 2, respectively (Supplementary Figure S25). The centroid frame for every cluster was then selected as the representative complex structure and labeled Cluster1 and Cluster2. To test the binding affinity of the designed inhibitors, we extracted the CDK6 structures of Cluster1 and Cluster2 as receptor structures for molecular docking. Ensemble docking (Vilar and Costanzi, 2013; Amaro et al., 2018) was performed to assess the effects of these modifications on inhibitor binding affinity. The ensemble docking method for the designed inhibitors considered Cluster1 and Cluster2 conformations. The RMSD of 2.01 Å between Cluster1 and Cluster2 indicates that the conformational difference was insignificant.

The selected representative complex structures for abemaciclib/CDK6 were used to evaluate the power of the docking process. The native conformation in the complex structures and the docking conformation were superimposed well (Supplementary Figure S26). This result confirmed that the docking process was robust for molecular docking in the design of inhibitors.

#### 3.5.1 One-region modification

Based on the binding model and different regions, 42 novel compounds with one-region modification (Supplementary Figure S27) were designed to inhibit the activity of CDK6. The lowest binding energy of Cluster1 and Cluster2 docking were defined as the docking scores for the designed compound (Supplementary Table S7). The score for abemaciclib (−9.46 kcal/mol) was employed as one standard value to determine whether the designed compound has a higher affinity than abemaciclib for targeting human CDK6. Most compounds had a docking score similar to that of abemaciclib, and a few designed compounds had better docking scores than abemaciclib.

As indicated, the docking score for the R1 region translation from benzene ring (abemaciclib) to pyridine ring (R1a), 1H-1, 2, 3-triazole ring (R1b), and 1H-pyrazole (R1i) decreased the binding affinity from −9.46 to −9.31, −9.31, and −8.58 kcal/mol, respectively, indicating that benzene and pyridine rings showed a similar binding affinity with V27 of CDK6. Conversely, 1-cyclopentyl-1H-[1, 2, 3] triazolo [4,5-b] pyridine (R1f) and 1-cyclopentyl-1H-pyrazolo [4,3-b] pyridine (R1m), which replaced 4-fluoro-1-isopropyl-2-methyl-1H-benzo [d] imidazole (abemaciclib), improved the activity by −10.03, −9.78, and −9.46 kcal/mol, respectively. Thus, the R1 region will be considered an isopropyl, cyclopropyl, and cyclopentyl group for the remaining similarity in binding affinity with human CDK6.

The amine-pyrimidine group plays a key role in abemaciclib binding to human CDK6 via hydrogen bonding with the hinge loop. Thus, this amine-pyrimidine group was reserved, and fluorine atoms were substituted with other groups to locate the hydrophobic cavity. Methyl (R2a), cyclopropyl (R2b), formyl (R2e), cyclopropylethan (R2f), ethyl (R2h), and hydrogen groups were used to occupy the hydrophobic cavity. Thus, the hydrogen atom, fluorine atom, and methyl group were selected as the favorable groups.

In addition, certain modifications have been made in the R3 region to improve binding affinity. R3e (fluorine group, −10.39 kcal/mol), R3f (methyl, −10.37 kcal/mol), and methoxy group (R3i, −10.28 kcal/mol) showed a higher dock score than abemaciclib (−9.46 kcal/mol). This region has been previously used to improve the inhibitory activity of protein kinases, for example, the addition of a methyl group in dasatinib. Thus, hydrogen, fluorine, and methoxy groups were considered better groups for the R3 region.

The R4 region of abemaciclib was unstable during the simulation. In molecular docking, the exposed amino group (R4a) increased the binding affinity to −10.31 kcal/mol. Isopropyl (R4b), methyl (R4g), cyclopropyl (R4i), and cyclopentyl (R4j) groups were also favorable for binding with ROCK6. Thus, the hydrogen, methyl, and isopropyl groups were considered suitable for the R4 region.

#### 3.5.2 Combination four regions

**FIGURE 5:**
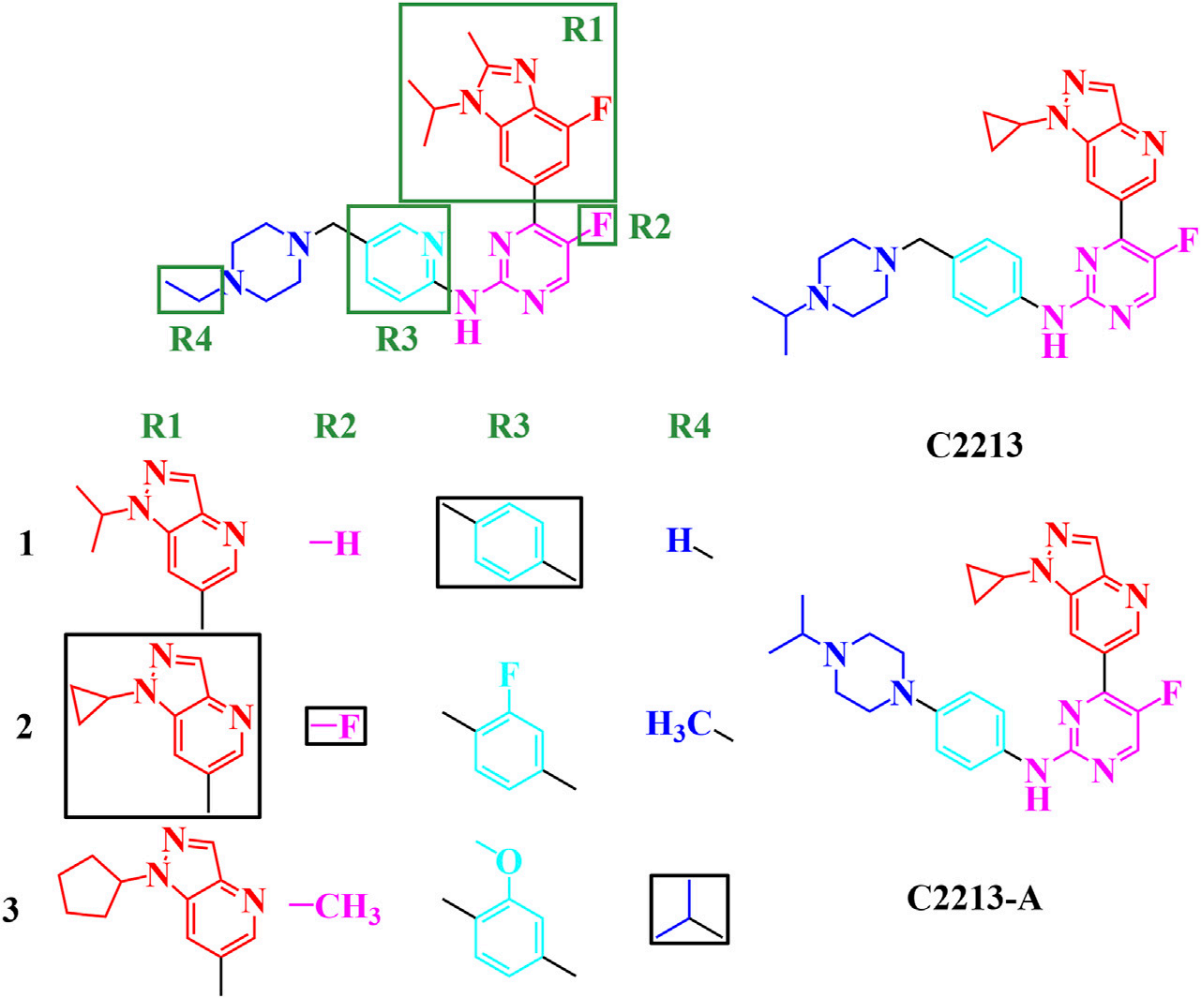
Combination strategies for designing novel inhibitors. The combination compound was named with the group numbers R1, R2, R3, and R4. For example, C1333 with an isopropyl group was named as R1, with a methyl group as R2, with a methoxy group as R3, and with an isopropyl group as R4.

From the single-region modification, some better groups were selected for R1 (isopropyl, cyclopropyl, and cyclopentyl groups), R2 (hydrogen, fluorine, and methyl groups), R3 (hydrogen, fluorine, and methoxy groups), and R4 (hydrogen, methyl, and isopropyl groups). In this step, a combination of R1, R2, R3, and R4 was employed to identify additional potential inhibitors. The strategies for this combination are shown in Figure 5; 81 compounds were identified in the combination strategies. The combination compounds were named as R1, R2, R3, and R4. For example, C1333 with an isopropyl group was named as R1 (1), that with a methyl group as R2 (3), that with a methoxy group as R3 (3), and that with an isopropyl group as R4 (3). These combination inhibitors have also been subjected to molecular docking using Cluster1 and Cluster2 receptors (Supplementary Table S8).

When R1 remained in the isopropyl group, four compounds (C1231, C1311, C1321, and C1331) had docking scores less than −10.00 kcal/mol. Meanwhile, three compounds had dock scores < −10.00 kcal/mol (C2121, C2213, and C2331) for R1 with the cyclopropyl group. R1 with the cyclopentyl group showed a stronger binding affinity than that with the isopropyl and cyclopropyl groups. Most (12/27) designed compounds had higher binding affinities than −10.00 kcal/mol. Thus, the cyclopentyl group was the best among the isopropyl, cyclopropyl, and cyclopentyl groups. However, these three groups had similar binding conformations in the ATP-binding site.

Six compounds had better binding energy than −10.00 kcal/mol with hydrogen, fluorine, and methyl groups for R2. The trifluoromethyl group for R2 was also found for roniciclib and SY-5609, and the halogen group for abemaciclib, QHRD107, THZ1, NVP2, and THZ531 (Supplementary Figure S28). Thus, the hydrogen, halogen, and methyl groups are suitable for the R2 site and occupy the hydrophobic cavity of human CDK6. Eight, six, and four designing inhibitors had calculated binding energy above −10.00 kcal/mol for R3 with hydrogen, fluorine, and methoxy groups, respectively. Thus, the benzene ring retains a π-alkyl interaction with I19.

Hydrogen atoms with R4 can be used to form hydrogen bonds with CDK6, which contributes to inhibitor binding. Thus, in this study, the secondary amine group exposed one hydrogen atom as a hydrogen bond donor and a tertiary amine without a hydrogen atom for hydrogen bonding. The fluctuation in the conformation of the piperazine ring of abemaciclib also indicates that the flexibility of this region is insufficient for abemaciclib binding to human CDK6.

By combining R1, R2, R3, and R4, the C2213 compound was selected as the superior inhibitor. Furthermore, the variant of C2213 without the methylene group between piperazine and benzene groups also decreased the fluctuation of the piperazine group, similar to compound C2213-A (Figure 5).

#### 3.5.3 Candidate inhibitors

**FIGURE 6:**
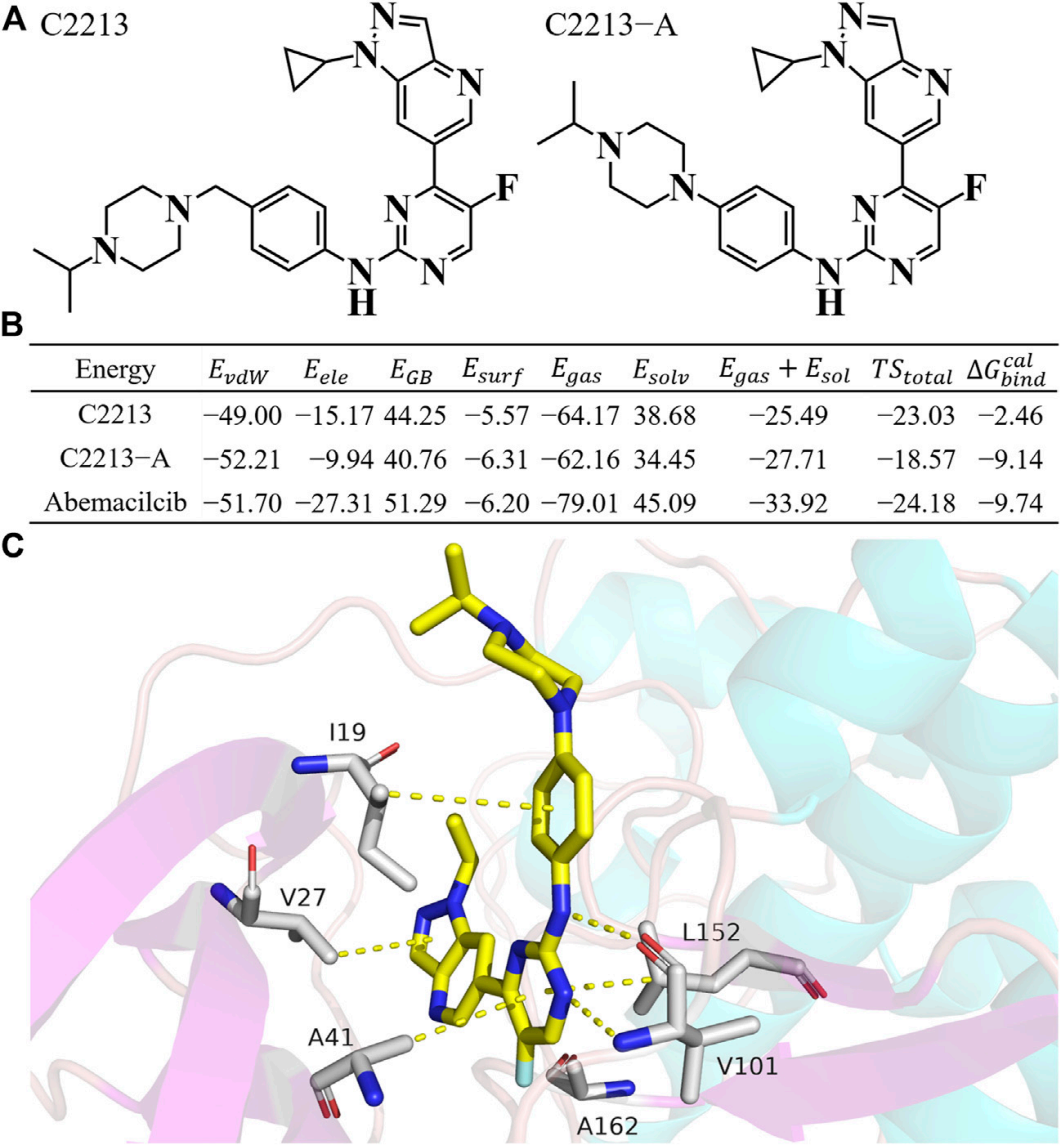
Binding free energy for C2213 and C2213-A. **(A)** Structures of C2213 and C2213-A. **(B)** Binding free energy for C2213 and C2213-A binding with human CDK6. **(C)** The interaction model for C2213-A from 100 ns frame.

Representative inhibitors, including C2213 and C2213-A, have a binding model with human CDK6 similar to that of abemaciclib (Supplementary Figure S29). These two complex systems (C2213/CDK6 and C2213-A/CDK6) were subjected to 100-ns MD simulations to calculate the binding free energies between C2213 and C2213-A and human CDK6. The RMSD vs time plot showed that the C2213/CDK6 and C213-A/CDK6 systems remained stable after 40 ns (Supplementary Figure S30). Frames 10, 20, 30, 40, 50, 60, 70, 80, 90, and 100 ns for the C2213/CDK6 and C2213-A/CDK6 systems were extracted. They revealed that C2213-A was tightly bound to the ATP-binding site of human CDK6, and C2213 conformation fluctuated in the pocket between the N-lobe and C-lobe of CDK6 (Supplementary Figures S31, S32). The side chain of the piperazine ring with C2213, which has a methylene group between the piperazine and benzene groups, exposed the piperazine group to the solvent. It disordered the hydrogen bond between the hinge loop of CDK6 and the amine-pyrimidine group of abemaciclib. In addition, the binding free energies were calculated based on the last 20 ns of the simulation trajectories (Figure 6; Supplementary Tables S9, S10). The binding free energy for C2213-A binding with CDK6 was −9.14 kcal/mol, which was 0.46 kcal/mol lower than that of abemaciclib ( $∆G_{bind}^{\exp}$ = −9.60 kcal/mol). This shows that C2213-A has a binding affinity similar to that of abemaciclib. In addition, $∆G_{bind}^{cal}$ of C2213 was −2.46 kcal/mol and showed a lower binding affinity than abemaciclib. Meanwhile, the binding free energy of C2213 was higher, by 6.68 kcal/mol, than that of C2213-A. These results indicated that the binding affinity can be increased owing to the inflexibility of the piperazine group. Subsequently, C2213-A was used for synthesis and pharmacological evaluation.

### 3.6 Pharmacological evaluation

C2213-A was selected as the candidate drug for the calculation studies. C2213-A {4-[1-cyclopropyl-1H-pyrazolo (4, 3-b) pyridin-6-yl]-5-fluoro-N-(4-(4-isopropylpiperazin-1-yl) phenyl) pyrimidin-2-amine} was synthesized as shown in Scheme 1. Detailed information can also be found in the Supplementary Material.

**FIGURE 7:**
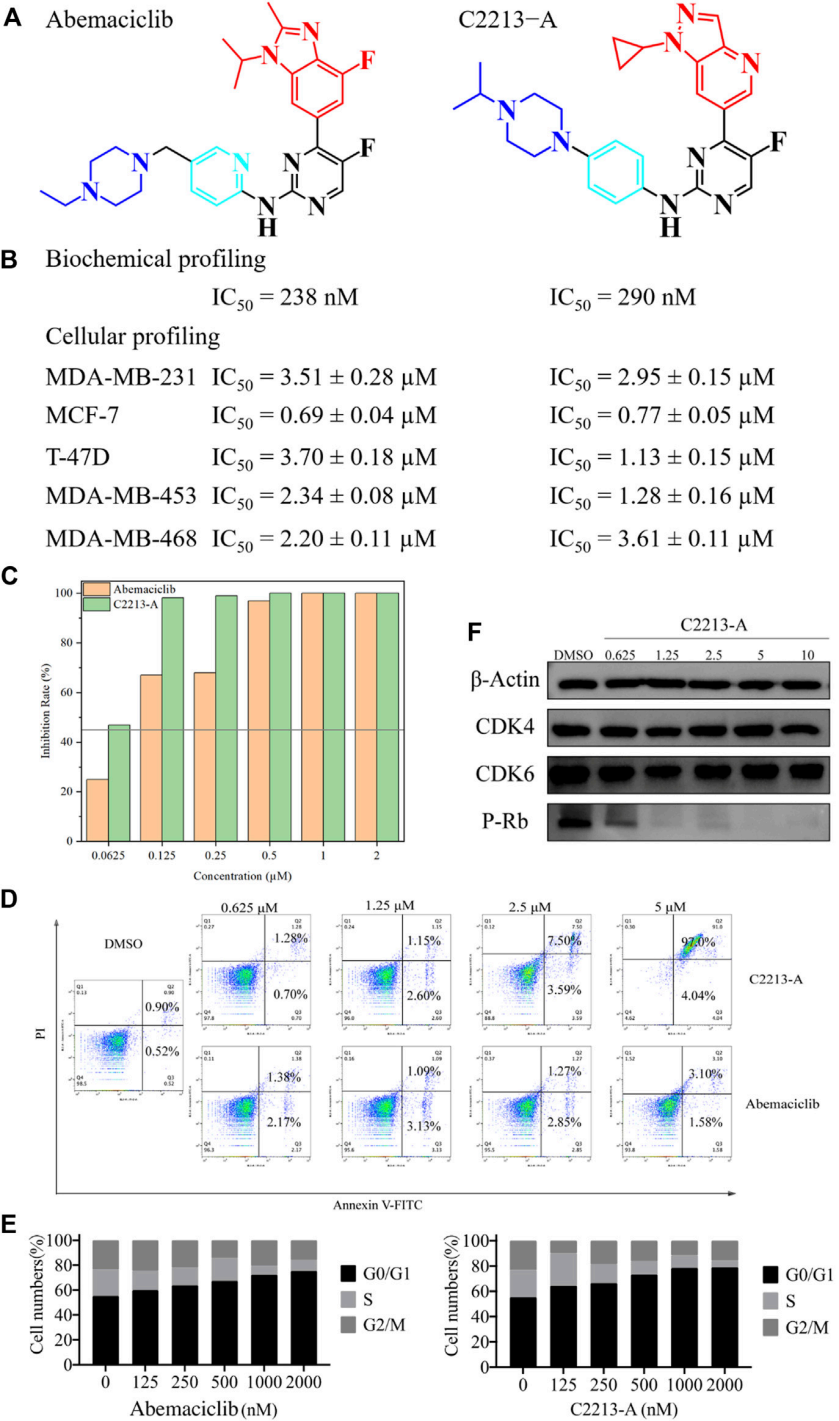
Pharmacological evaluation of C2213-A and abemaciclib. **(A)** Structures of abemaciclib and C2231-A. **(B)** Biochemical and cellular profiling of abemaciclib and C2231-A. **(C)** Colony-formation assays for MDA-MB-231 with different concentrations of C2231-A. **(D)** Percentage of apoptotic cells after treatment with different concentrations of compound C2213-A for 48 h **(E)** MDA-MB-231 cell lines exhibited obvious G1 arrest and a decrease in S phase cells after incubation with different doses of compound C2213-A or abemaciclib. **(F)** Compound C2213-A inhibited the phosphorylation of Rb at S780 in a dose-dependent manner in MDA-MB-231.

The IC_50_ values of abemaciclib and C2213-A against human CDK6 were evaluated using the commercial KinaseProfiler Service. The IC50 value for C2213-A was 290 nM, similar to the estimate of 238 nM for abemaciclib targeting human CDK6/cyclin D3 (Figure 7 and Supplementary Figure S33). This agreed with the binding free energy calculation with −9.14 and −9.74 kcal/mol for C2213-A and abemaciclib, respectively. Thus, C2213-A was identified as an abemaciclib derivative for continued optimization to target CDK6.

In addition, the antiproliferative effects of abemaciclib and C2213-A were evaluated in MDA-MB-231 (a human breast cancer cell line). The antiproliferative activity of C2213-A was significantly higher than that of abemaciclib alone, with IC_50_ values of 2.95 ± 0.15 µM and 3.51 ± 0.28 µM, respectively (Figure 7). In MCF-7 cells, the IC_50_ values of abemaciclib and C2213-A were 0.69 ± 0.04 µM and 0.77 ± 0.05 µM, respectively (Supplementary Figure S33). To further demonstrate the broad applications of our findings, the inhibitory activities of abemaciclib and C2213-A were tested using T-47D, MDA-MB-453, and MDA-MB-468 cell lines. C2231-A showed a better inhibitory effect than that of abemaciclib in T-47D (IC_50_ values were 1.13 ± 0.15 µM and 3.70 ± 0.18 µM, respectively). However, C2231-A was less effective than abemaciclib in inhibiting the growth of MDA-MB-468 cells (3.61 ± 0.11 µM and 2.20 ± 0.11 µM, respectively). Moreover, C2231-A showed activity similar to that of abemaciclib in MDA-MB-453 cells (1.28 ± 0.16 µM and 2.34 ± 0.08 µM, respectively). These observations indicated that the inhibitory activities of C2231-A and abemaciclib differ among cell lines and suggest that C2213-A inhibits the proliferation of MDA-MB-231, MCF-7, T-47D, MDA-MB-453, and MDA-MB-468 cells.

A clone formation assay was conducted using MDA-MB-231 cells to investigate the inhibitory activity of C2213-A, with abemaciclib for comparison (Supplementary Figure S34). The proliferation of MDA-MB-231 cells was impaired (inhibition rate >45%) by abemaciclib (62.5 nM) and C2231-A (125 nM) compared with that in the control group (Figure 7). These results suggest that C2213-A inhibits the activity of MDA-MB-231 cells.

An annexin V-FITC/propidium iodide staining assay using human breast tumor MDA-MB-231 cells showed that approximately 83% of treated cells underwent apoptosis after a 48-h treatment with compound C2213-A at a concentration of 5 μM (Supplementary Figure S35). These findings confirmed that the anti-proliferative effect of C2213-A is most likely due to the induction of apoptosis.

We also compared the effects of compound C2213-A and abemaciclib on the cell cycle profile of MDA-MB-231 cells by flow cytometry (Supplementary Figure S36). These findings revealed that both compounds elicited a dose-dependent effect, leading to the accumulation of cells in the G1 phase, as expected.

The effects of compound C2213-A on CDK4/6 and the phosphorylation of Rb (S780) were assessed by a Western blot assay on MDA-MB-231 cells. Compound C2213-A blocked the CDK4/6/Rb/E2F signaling pathway in a dose-dependent manner after 24 h of incubation (Supplementary Figure S37). Treatment with compound C2213-A resulted in the dose-dependent blockage of Rb phosphorylation, consistent with its targeted mechanism of action on CDK4/6 in cells.

## 4 Conclusion

CDK4/6 is disordered in numerous cancers, such as breast cancer, osteosarcoma, and acute megakaryoblastic leukemia. CDK4/6 is considered an effective anticancer drug target. Three CDK4/6 inhibitors have been approved; however, there is still a gap between the clinical requirements and approved CDK4/6 drugs. Thus, selective and oral CDK4/6 inhibitors urgently need to be developed, particularly for monotherapy. This study investigated the interaction between abemaciclib and human CDK6 using MD simulations. Simultaneously, the binding free energy and energy decomposition were calculated using the MM/GBSA approach. V101 and H100 formed stable hydrogen bonds with the amine pyrimidine group of abemaciclib. K43 interacted with an imidazole ring via an unstable hydrogen bond. Meanwhile, I19 (pyridine ring), V27 (imidazole ring), A41 (pyrimidine ring), and L152 (pyrimidine ring) interacted with abemaciclib via an π-alkyl force. Based on the binding model of abemaciclib/CDK6. Abemaciclib treatment was divided into four regions; 43 compounds were designed with only one region modification. Three favorable groups for each region were selected from the docking score for single-region modification. Subsequently, a combination of the favorable groups within the four regions was performed, and 81 compounds were obtained. Among them, C2231 emerged as a better compound from the four-region combination strategy. C2231-A, which lacks a methylene group compared to C2213, showed superior flexibility reduction. Additionally, kinase profiling and cell profiling of MDA-MB-231 and MCF-7 cells were performed after synthesizing C2231-A. C2231-A caused greater inhibition than did abemaciclib in MDA-MB-231 cells. Based on MD simulation, one candidate compound was designed, which showed a significant inhibitory effect on a human breast cancer cell line. However, computational tools and experimental methods must be used to identify more compounds based on C2231-A. In the future, C2213-A derivatives will be studied using molecular docking, MD simulation, binding free energy calculation, kinase, and cellular profiling.

## Data Availability

The datasets presented in this study can be found in online repositories. The names of the repository/repositories and accession number (s) can be found in the article/Supplementary Material.
