# Supplementary material for: Identification of abemaciclib derivatives targeting cyclin-dependent kinase 4 and 6 using molecular dynamics, binding free energy calculation, synthesis, and pharmacological evaluation
Source: Front Pharmacol. 2023 May 10;14:1154654. doi: 10.3389/fphar.2023.1154654 (PMC10206264; doi:10.3389/fphar.2023.1154654)
Supplement: Supplementary file 1 [file DataSheet1.PDF]

Identification of abemaciclib derivatives targeting cyclin-dependent kinase 4 and 6 using molecular dynamics, binding free energy calculation, synthesis, and pharmacological evaluation

Yanting Zhou[1,#], Xiandeng Li[2,#], Peifang Luo[3], Huiting Chen[2], Yan Zhou[4], Xueting Zheng[4], Yuan Yin[4], Haoche Wei[5], Hongji Liu[6], Wen Xia[1,*], Mingsong Shi[4,*], Xiaoan Li[4]

[1] Key Laboratory of Basic Pharmacology of Ministry of Education and Joint International Research Laboratory of Ethnocentric of Ministry of Education, Zunyi Medical University, Zunyi, Guizhou, 563006, China
[2] College of Pharmacy, Chongqing Medical University, Chongqing, 400016, China
[3] Department of Cardiovascular Surgery, Affiliated Hospital of Zunyi Medical University, Zunyi, Guizhou, 563099, China.
[4] NHC Key Laboratory of Nuclear Technology Medical Transformation, Mianyang Central Hospital, School of Medicine, University of Electronic Science and Technology of China, Mianyang, Sichuan, 621000, China
[5] State Key Laboratory of Biotherapy/Collaborative Innovation Center of Biotherapy and Cancer Center, West China Hospital, Sichuan University, Chengdu, Sichuan, 610041, China
[6] Department of Ophthalmology, Mianyang Central Hospital, School of Medicine, University of Electronic Science and Technology of China, Mianyang, Sichuan, 621000, China

[#] These authors have contributed equally to this work.
* Correspondence:
Corresponding Authors: Wen Xia, Mingsong Shi
xxxyxkk@163.com, therotyonth@163.com

Contents

Molecular Dynamics Simulation S6
Cluster Analysis S7
Binding Free Energy Estimation S7
Chemistry S8
1-isopropyl-4-(4-nitrophenyl)piperazine (**3**) S9
4-(4-isopropylpiperazin-1-yl)aniline (**4**) S9
5-bromo-2-methylpyridin-3-amine (**6**) S9
N-(5-bromo-2-methylpyridin-3-yl)acetamide (**7**) S10
1-(6-bromo-1H-pyrazolo[4,3-b]pyridin-1-yl)ethan-1-one (**8**) S10
6-bromo-1H-pyrazolo[4,3-b]pyridine (**9**) S10
6-bromo-1-cyclopropyl-1H-pyrazolo[4,3-b]pyridine (**10**) S10
1-cyclopropyl-6-(4,4,5,5-tetramethyl-1,3,2-dioxaborolan-2-yl)-1H-pyrazolo[4,3-b]pyridine (**11**) S11
6-(2-chloro-5-fluoropyrimidin-4-yl)-1-cyclopropyl-1H-pyrazolo[4,3-b]pyridine (**12**) S11
4-(1-cyclopropyl-1H-pyrazolo[4,3-b]pyridin-6-yl)-5-fluoro-N-(4-(4-isopropylpiperazin-1-yl)phenyl)pyrimidin-2-amine (**C2213-A**) S11
Cellular proliferation assays S12
Colony formation assay S12
Figure S1. Structures of CDK4/6 inhibitors. S16
Figure S2. Methods used in this work. S17
Figure S3. Root mean square deviation (RMSD) value of heavy atoms of backbone for human CDK6 and that of abemaciclib from 500 ns MD simulation for abemaciclib/CDK6 system S18
Figure S4. Root mean square deviation (RMSD) value of heavy atoms of backbone for human CDK6 and along 500 ns MD simulation for abemaciclib/CDK6 and Apo-CDK6 systems. S19
Figure S5. Root mean square deviation (RMSD) value of heavy atoms of backbone for human CDK6 and that of abemaciclib along with 500 ns MD simulation for two replicated abemaciclib/CDK6 systems (abemaciclib/CDK6-2 and abemaciclib/CDK6-3 systems) S20
Figure S6. Root mean square deviation (RMSD) value of heavy atoms of backbone for

different region (N-lobe and C-lobe of kinase domain for human CDK6) of human CDK6 along with 500 ns MD simulation for abemaciclib/CDK6 system................S21
Figure S7. Crystal structures of inhibitor/CDK6 complex with T-loop....................S22
Figure S8. Crystal structures of inhibitor/CDK6 complex with K43-E61 salt bridge. S23
Figure S9. Root mean square deviation (RMSD) value of heavy atoms of backbone for αC domain for human CDK6 along with 500 ns MD simulation for abemaciclib/CDK6 system. S24
Figure 10. Distance between K43 and E61 along with the simulation time.............S25
Figure S11. Snapshots of the abemaciclib/CDK6 along the dynamic simulation time for 100, 200, 300, 400, and 500 ns. S26
Figure S12. Hydrogen bond analysis for abemaciclib/CDK6 system. S27
Figure S13. Hydrogen bond analysis for the last 200 ns simulation of abemaciclib/CDK6 system..S28
Figure S14. Distance for the hydrogen bonds between abemaciclib and hinge loop of human CDK6 in abemaciclib/CDK6 system. S29
Figure S15. Angle for the hydrogen bonds between abemaciclib and hinge loop of human CDK6 in abemaciclib/CDK6 system. S30
Figure S16. Cluster analysis results for the last 200 ns simulation. S31
Figure S17. Hydrogen bond network for D163. ....S33
Figure S18. Conformation of D163 and R168 of the abemaciclib/CDK6 along the dynamic simulation time for 100, 200, 300, 400, and 500 ns..................................S34
Figure S19. Palbociclib binding with human CDK6. ....S35
Figure S20. Distance between pyridine ring of abemaciclib and side chain of I19...S36
Figure S21. Distance between V27 and imidazole ring of abemaciclib along 500 ns simulation...S37
Figure S22. Distance and angle for A41, L152 and pyrimidine ring of abemaciclib for abemaciclib/CDK6 system..S38
Figure S23. Interaction between abemaciclib and hydrophobic cavity of CDK6. ....S39
Figure S24. Piperazine group of abemaciclib interaction with K29/D104 of human CDK6. ....S40
Figure S25. Cluster analysis results for the last 200 ns simulation. S41
Figure S26. Re-dock results for the selected two abemaciclib/CDK6 representative

models. S42
Figure S27. Designed novel CDK6 inhibitors based on abemaciclib with one region. S43
Figure S28. Structure for CDK inhibitors with different R2 groups. S44
Figure S29. Lowest binding energy conformation of C2213 and C2213-A from molecular docking. S45
Figure S30. Root mean square deviation (RMSD) value of heavy atoms of backbone for human CDK6 and along 500 ns MD simulation for **C2213**/CDK6 and **C2213-A**/CDK6 systems. S46
Figure S31. Snapshots of **C2213**/CDK6 along the dynamic simulation time for 10, 20, 30, 40, 50, 60, 70, 80, 90, and 100 ns. S47
Figure S32. Snapshots of **C2213-A**/CDK6 along the dynamic simulation time for 10, 20, 30, 40, 50, 60, 70, 80, 90, and 100 ns. S48
Figure S33. Biochemical and cellular profiling for abemaciclib and C2213-A for MDA-MB-231, MCF-7, T-47D, MDA-MB-453, and MDA-MB-468.. ....S49
Figure S34. Colony-formation assays for MDA-MB-231 with different concentration C2231-A and abemaciclib. S50
Figure S35. Percentage of apoptotic cells treatment with different concentration of compound C2213-A for 48 h. S51
Figure S36. MDA-MB-231 cell lines exhibited an obvious G1 arrest and a decrease of S phase after incubation with different doses of compound C2213-A or abemaciclib. S52
Figure S37. Compound C2213-A inhibited the phosphorylation of Rb at S780 in a dose-dependent manner in MDA-MB-231. ....S53
Table S1. Binding free energies (ΔGbindcal) for abemaciclib/CDK6 system. S54
Table S2. Binding free energies (ΔGbindcal) for abemaciclib/CDK6 system (replicate 2). S55
Table S3. Binding free energies (ΔGbindcal) for abemaciclib/CDK6 system (replicate 3). S56
Table S4. Binding free energies (ΔGbindcal) for abemaciclib/CDK6 system as cluster 1..S57
Table S5. Binding free energies (ΔGbindcal) for abemaciclib/CDK6 system as cluster 2..S58

Table S6. Free energy decomposition for abemaciclib/CDK6 complex on the individual residue basis. S59
Table S7. Docking results for the designing compounds from one region. S60
Table S8. Docking results for the designing compounds with four region combination. S62
Table S9. Binding free energies (ΔGbindcal) for **C2213**/CDK6 system. S66
Table S10. Binding free energies (ΔGbindcal) for C2213-A/CDK6 system. S67

Molecular Dynamics Simulation

To correctly describe the abemaciclib force field, the general Amber force field (GAFF2)[1] generation procedure was used. The geometry structure was optimized at B3LYP/6-31G* level of theory using Gaussian 09[2]. To obtain the partial atomic charges, the restrained electrostatic potential (RESP) protocol[3] was employed at the HF/6-31G* level of theory. The force field parameters were generated using the Antechamber module. Meanwhile, the AMBER ff19SB force field[4] was used to create topology parameters of human CDK6. The missing residues in the crystal structures of CDK6 are repaired with the SWISS-MODEL online servers[5-7]. The protonation state of each residue of CDK6 was generated according to a pH of 7.4 using the H++ online service[8-11]. The hydrogen atoms unavailable from the crystal structures are bonded to their corresponding heavy atoms with the tool Leap in AmberTools 21. Firstly, the complex systems were dissolved in a TIP3P water[12] with cuboid box, and the total box size was about 102 Å × 87 Å × 87 Å. The systems were neutralized using one Na^+ ions and the final system included 4750 solute atoms and 19723 solvent water molecules. The periodic boundary conditions were applied to avoid edge effects and a cut-off radius of 12 Å was employed for van der Waals interactions. The particle mesh Ewald (PME) algorithm[13] also was used in calculating the long-range electrostatic interactions. The SHAKE algorithm[14] was used to constrain the covalent bond involved with hydrogen atoms. To reduce the effect of unfavorable interactions produced by solvents and ion, the system was subjected to 9000 steps of steepest descent method and then 1000-step conjugate gradient, while fixing all the solute molecules at the initial position. Then the 10000-step conjugate gradient method was used to optimize the whole system including solute molecule and solvent molecule. After the first two-step minimization of the system, the overall system temperature was heated from 0 K to 300 K in 200 ps with Langevin dynamics [15, 16]with the collision frequency g = 2.0 ps^{-1}. The pressure was then kept at 1 bar in 200 ps with isotropic position scaling[17] and temperature at 300 K. After that, the system was equilibrated at 300 K and 1 bar within the NPT (isothermal-isobaric) ensemble with 200 ps. Subsequently, the entire system underwent the 500 ns molecular dynamics simulation for final data collection and analyses. The integration step size was set to 2 fs throughout the molecular dynamics. The random seed was based on the current date and time for every nanosecond simulation. All dynamics were performed using the CUDA version of the PMEMD in AMBER 20[18]. The *CPPTRAJ* module[19, 20]

was used to analysis the data from the MD trajectories.

Cluster Analysis

Cluster analysis is a general unsupervised technique for finding patterns within data. In this work, the root mean-square deviation (RMSD)-based clustering was performed with *CPPTRAJ* module [19, 20], a simulation analysis tools implemented in AMBERTools 21[18]. As one of the most popular clustering algorithms, the average linkage cluster algorithm was used herein. Each cluster contained a representative structure whose RMSD was equidistant to all other cluster members. Structures were collected by sampling at 10 ps intervals in the last 200 ns simulation with 20000 frames, and cluster analysis was based on the heavy atoms of abemaciclib/CDK6 complex to generate some clusters with epsilon = 2.0 Å.

Binding Free Energy Estimation

In this work, the molecular mechanics generalized Born surface area (MM/GBSA) approach[21, 22] were employed to calculate the binding free energies of the inhibitor abemaciclib to human CDK6. It is an efficient method to improve the ability evaluation of ligand and enzyme systems[23-25]. The MM/GBSA framework has been discussed extensively[26-28]. Only a short description is summarized here. The total binding free energy for the binding of inhibitor of human CDK6, namely $\Delta G_{binding}$ was provide in the following:

$$\Delta G_{binding} = \Delta G_{complex} - \Delta G_{protein} - \Delta G_{ligand} \quad (1)$$

$$G = E_{gas} + E_{sol} - TS \quad (2)$$

$$E_{gas} = E_{int} + E_{vdW} + E_{ele} \quad (3)$$

$$E_{sol} = E_{GB} + E_{surf} \quad (4)$$

$$E_{surf} = \gamma \cdot \mathrm{SA} + \mathrm{b} \quad (5)$$

$\Delta G_{complex}$, $\Delta G_{protein}$ and ΔG_{ligand} are denoted as free energies of the abemaciclib/CDK6, CDK6 and inhibitor abemaciclib, respectively. The $\Delta G_{binding}$ can be decomposed into the enthalpy part ($\Delta H = \Delta E_{gas} + \Delta E_{sol}$) and the entropy part ($T\Delta S$). The molecular mechanical energies (E_{gas}) consist of the intramolecular energy (E_{int}), van der Waals forces (E_{vdW}) and electrostatic forces (E_{ele}) which get these values via a statistical average way based on the AMBER force field. The solvation free

energy (E_{sol}) can be principally divided into both electrostatic (E_{GB}) and non-electrostatic (E_{surf}) terms. The E_{surf} comes from the combined effect of unfavorable cost of surface formation and the favorable van der Waals interactions between the solute and solvent, which can be evaluated by the equation of $\gamma \cdot SA + b$, where γ =0.0072 kcal/Å^2 and b=0.0 kcal/mol. The solvent accessible surface area (SA) which was estimated using the linear combination of pairwise overlaps (LCPO) method[29]. The E_{GB} is calculated by the Generalized Born (GB) equation[30, 31] with igb =8[32, 33]. The solute dielectric constant was set to 1, and the exterior dielectric constant was set to 80. We used MM/GBSA method to get these former terms via a statistical average way from the last 100 ns MD trajectory. Meanwhile, entropy contributions to the binding free energy may be added to improve the accuracy. The entropy can be estimated using the normal model analysis with quasi harmonic model based on the same MD trajectory[34]. For each complex system, binding energies were averaged over 1000 frames and the $-TS$ was averaged with interval 2 ns (100 snapshots). Those energy terms were calculated with the MMPBA.py program[35].

Chemistry

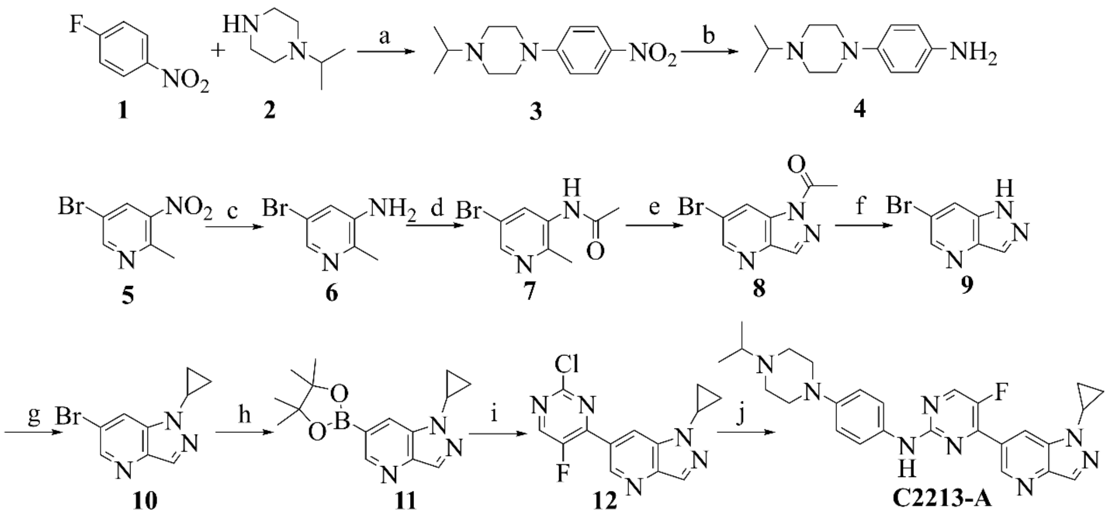

Scheme 1. Synthetic route of compounds **C2213-A**.

Reagents and conditions: (a) K_2CO_3, MeCN, 80 °C; (b) Pd/C, H_2, rt; (c) Fe/NH_4Cl, MeOH/H_2O, 80 °C; (d) AcOK, acetic anhydride, $CHCl_3$, reflux; (e) isopentyl nitrite, 18-Crown-6, K_2CO_3, $CHCl_3$, reflux; (f) KOH, H_2O, rt; (g) cyclopropylboronic acid, Na_2CO_3, $Cu(AcO)_2$, 2,2'-Bipyridine, DCM, 80 °C; (h) bis(pinacolato)diboron, $PdCl_2$(dppf), AcOK, dioxane, 90 °C; (i) 2,4-Dichloro-5-fluoropyrimidine, $PdCl_2$(dppf),

K_2CO_3, dioxane/water (V/V, 10/1), 80 °C; (j) compound **4**, $Pd_2(dba)_3$, Xantphos, Cs_2CO_3, dioxane, reflux.

1-isopropyl-4-(4-nitrophenyl)piperazine (**3**)

Dissolved 1-fluoro-4-nitrobenzene (**1**) (1.4 g, 10 mmol, 1.0 equiv) in 40 mL of acetonitrile, then added potassium carbonate (3.5 g, 25 mmol, 2.5 equiv) and 1-isopropylpiperazine (**2**) (1.5 g, 12 mmol, 1.2 equiv). The reaction mixture was heated at 80 °C for reflux for 60 min. After the reaction, poured the reaction solution into 100mL water, precipitated solid, filtered by suction, washed the filter cake twice, and then dried it. 2.0 g of yellow solid was obtained, yield 81%. The product was used directly in the next reaction without purification.

4-(4-isopropylpiperazin-1-yl)aniline (**4**)

Intermediate **3** (2.0 g, 8.0 mmol, 1.0 equiv) was dissolved in 20 mL of methanol, then 10% palladium carbon (0.8 g, 0.1 equiv) was added and reacted at room temperature in hydrogen atmosphere for 24 h. After the reaction, filtered by suction, concentrated the organic phase under reduced pressure to colorless oil, and washed it with 20 mL of petroleum ether. Suction filtration and drying it gave the target product **4** 1.49 g as a brown solid. yield 85%. ^{1}H NMR (400 MHz, DMSO-d_6) δ 6.70 – 6.63 (m, 2H), 6.51 – 6.44 (m, 2H), 4.53 (s, 2H), 2.88 (dd, J = 6.0, 3.9 Hz, 4H), 2.67 (s, 1H), 2.57 – 2.50 (m, 4H), 0.99 (d, J = 6.6 Hz, 6H).

5-bromo-2-methylpyridin-3-amine (**6**)

2-methyl-3-nitro-5-bromopyridine (**5**) (13 g, 60 mmol, 1.0 equiv), reduced iron powder (8.4 g, 150 mmol, 2.5 equiv) and ammonium chloride (8.1 g, 150 mmol, 2.5 equiv) were added to 100 mL methanol/water (V/V = 4:1), then transferred to 80 °C reflux reaction for 2 h. After the reaction was completed, it was filtered under hot suction, and then the filtrate was concentrated under reduced pressure to dryness of most solvent. Subsequent addition of 100 mL water, suction filtration and drying it gave a white solid 10.6 g, yield 95%. ^{1}H NMR (400 MHz, $CDCl_3$) δ: 8.29 (d, J = 1.9 Hz, 1H), 7.29 (d, J = 2.1 Hz, 1H), 5.41 (s, 2H), 2.28 (s, 3H).

N-(5-bromo-2-methylpyridin-3-yl)acetamide (**7**)

Intermediate **6** (10.6 g, 56.7 mmol, 1.0 equiv) and potassium acetate (14.0 g, 141.8 mmol, 2.5 equiv) were added to 100 mL chloroform, followed by the addition of acetic anhydride (7 mL, 68.0 mmol, 1.2 equiv), and the mixt was stirred at 60 °C for 1 h. After the reaction was finished, it was drawn and filtered. Then, the filtrate was concentrated to an oil under reduced pressure and reacted directly in the next step without further purification.

1-(6-bromo-1H-pyrazolo[4,3-b]pyridin-1-yl)ethan-1-one (**8**)

Intermediate **7** (56.7 mmol, 1.0 equiv) was added to 100 mL chloroform, followed by isoamyl nitrite (9.2 mL, 68.0 mmol, 1.2 equiv), 18-crown-6 (1.5 g, 56.7 mmol, 0.1 equiv) and potassium carbonate (19.5 g, 141.8 mmol, 2.5 equiv), and then the reaction was stirred at 60 °C for 12 h. After the reaction was completed, the liquid was concentrated under reduced pressure, and the crude silica gel was stirred for sample. Pooled sample was separated on a 100 g flashmaster personal + column (mobile phase system PE/EA = 80:20). The target intermediate **8** (10.2 g) was obtained, yield 75%. ^{1}H NMR (400 MHz, DMSO-d_6) δ: 8.67 (d, J = 1.9 Hz, 1H), 8.45 (d, J = 1.6 Hz, 2H), 2.74 (s, 3H).

6-bromo-1H-pyrazolo[4,3-b]pyridine (**9**)

Intermediate **8** (10.2 g, 42.5 mmol, 1.0 equiv) was added to 100 mL methanol/water (V/V = 1:1), followed by potassium hydroxide (4.3 g, 106.3 mmol, 2.5 equiv), and the reaction was stirred at room temperature for 2 h. After the reaction was completed, added 100 mL water and extracted by adding 200 mL dichloromethane. The organic phase was concentrated to a pale-yellow solid under reduced pressure to give the target product **9** (8.0 g), yield 95%. ^{1}H NMR (400 MHz, DMSO-d_6) δ 13.48 (s, 1H), 8.59 (d, J = 1.9 Hz, 1H), 8.35 (d, J = 1.6 Hz, 2H).

6-bromo-1-cyclopropyl-1H-pyrazolo[4,3-b]pyridine (**10**)

Dissolved 6-bromo-1*H*-pyrazolo[4,3-*b*]pyridine (**9**) (800 mg, 4.0 mmol, 1.0 equiv), cyclopropylboronic acid (695 mg, 8.1 mmol, 2.0 equiv), Na_2CO_3 (857 mg, 8.1 mmol, 2.0 equiv), $Cu(AcO)_2$ (735 mg, 4.0 mmol, 1.0 equiv) and 2,2'-Bipyridine (630 mg, 4.0 mmol, 1.0 equiv) in 20 mL DCM, and then stir at 80 °C for 3 h. After the reaction was

completed, the reaction liquid was concentrated under reduced pressure, and the crude silica gel was stirred for sample. Pooled sample was separated on a 25 g flashmaster personal + column (mobile phase system PE/EA = 90:10) to give the white target intermediate **10** (680 mg), yield 71%. ^{1}H NMR (400 MHz, DMSO-d_6) δ 8.59 (d, J = 2.0 Hz, 1H), 8.57 – 8.45 (m, 1H), 8.27 (s, 1H), 3.83 (p, J = 4.8 Hz, 1H), 1.12 (dd, J = 5.9, 3.4 Hz, 4H).

1-cyclopropyl-6-(4,4,5,5-tetramethyl-1,3,2-dioxaborolan-2-yl)-1H-pyrazolo[4,3-b]pyridine (**11**)

Intermediates **10** (680 mg, 2.9 mmol, 1 equiv), bis(pinacolato)diboron (1.1 g, 4.3 mmol, 1.5 equiv), potassium acetate (545 mg, 5.8 mmol, 2.0 equiv) and $PdCl_2$(dppf) (105 mg, 0.14 mmol, 0.05 equiv) were dissolved in 20 mL 1,4-dioxane and then the reaction was stirred at 90 °C for 12 h. After the reaction, the organic phase was filtered and concentrated under reduced pressure which was directly subjected to the next Suzuki reaction.

6-(2-chloro-5-fluoropyrimidin-4-yl)-1-cyclopropyl-1H-pyrazolo[4,3-b]pyridine (**12**)

Intermediate not purified in the previous step (**11**) (1.3 equiv), 2,4-dichloro-5-fluoropyrimidine (373 mg, 2.2 mmol, 1.0 equiv), $PdCl_2$(dppf) (81 mg, 0.11 mmol, 0.05 equiv) and potassium carbonate (616 mg, 4.5 mmol, 2.0 equiv) were dissolved in dioxane/water (V/V, 10/1). The reaction liquid was stirred under nitrogen atmosphere at 80 °C for 2h. After the reaction was complete, the reaction liquid was concentrated under reduced pressure and purified by silica gel column with the mobile phase system (DCM: MeOH = 47: 3). Finally, intermediate **12** (479 mg) was obtained. White solid, yield 75%. ^{1}H NMR (400 MHz, DMSO-d_6) δ 9.11 – 9.03 (m, 1H), 8.72 – 8.65 (m, 1H), 8.42 – 8.34 (m, 1H), 7.99 – 7.89 (m, 1H), 4.11 – 3.88 (m, 1H), 1.19 (s, 1H), 1.18 (s, 1H), 1.15 (s, 2H).

4-(1-cyclopropyl-1H-pyrazolo[4,3-b]pyridin-6-yl)-5-fluoro-N-(4-(4-isopropylpiperazin-1-yl)phenyl)pyrimidin-2-amine (**C2213-A**)

Intermediate **12** (100 mg, 0.34 mmol, 1.0 equiv), intermediate **4** (61 mg, 0.28 mmol, 0.8 equiv), $Pd_2(dba)_3$ (48 mg, 0.05 mmol, 0.15 equiv), Xantphos (60 mg, 0.1 mmol, 0.3 equiv) and Cs_2CO_3 (226 mg, 0.7 mmol, 2.0 equiv) were dissolved in dioxane.

The reaction liquid was stirred under nitrogen atmosphere at 100 °C for overnight. After the reaction was complete, the reaction liquid was concentrated under reduced pressure and purified by silica gel column with the mobile phase system (DCM/MeOH = (95:5)). Finally, the target compound **C2213-A** 68 mg was obtained. Yellow solid, yield 50%. ^{1}H NMR (400 MHz, DMSO-d_6) δ 9.62 (s, 1H), 9.14 (t, J = 1.7 Hz, 1H), 8.70 (s, 1H), 8.65 (d, J = 3.3 Hz, 1H), 8.37 (s, 1H), 7.62 – 7.57 (m, 2H), 6.93 – 6.88 (m, 2H), 3.96 – 3.89 (m, 1H), 3.06 (t, J = 5.1 Hz, 4H), 2.66 (p, J = 6.5 Hz, 1H), 2.60 – 2.54 (m, 4H), 1.22 – 1.16 (m, 4H), 1.00 (d, J = 6.6 Hz, 6H). ^{13}C NMR (101 MHz, DMSO-d_6) δ 172.64, 157.36, 147.04, 145.78, 142.61, 133.61, 133.03, 132.80, 126.69, 120.76, 118.74, 116.25, 113.86, 54.12, 49.84, 48.61, 39.37, 30.23, 18.74, 6.50. HRMS (ESI) *m*/*z* calculated for $C_{26}H_{29}FN_8$ [M+H]$^+$ 473.2572, found 473.2573.

Cellular proliferation assays

Human breast cancer cell line MDA-MB-231, MCF-7, T-47D, MDA-MB-453, and MDA-MB-468 were obtained from the American Type Culture Collection (ATCC; Manassas, Virginia, USA). The cells were cultured in Dulbecco's modified Eagle's medium (DMEM, for MDA-MB-231) or minimum essential medium (MEM, for MCF-7), which was supplemented with 1% Penicillin-Streptomycin (PS) and 10% Fetal bovine serum (FBS). Approximate 4×10^3 cells, the cells were plated into each well in 96-well plate and were incubated in 5% CO_2 at 37 °C for 24 h. The synthesized **C2213-A** at the indicated final concentrations were added to the culture medium and incubated for 72 h. The (3-(4,5-dimethylthiazol-2-yl)-2,5-diphenyl-2H-tetrazolium bromide) (MTT) was added in each cell. Subsequently, the cells were incubated for an additional 2 h in each well which were dissolved in 100 μL dimethyl sulfoxide (DMSO). The absorbance values (OD) of the 96-well tissue culture plates were read at 570 nm on a Spectra MAX M5 microplate spectrophotometer. The cell viability results were calculated using the GraphPad Prism 6.0 software. Each IC_{50} value was expressed as mean ± SD.

Colony formation assay

MDA-MB-231 was seeded in a six-well plate with a concentration of 1000 cells per well and cultured at 37 °C with 5% CO_2 in incubator for 24 h. The candidate compound **C2213-A** with different concentrations (0、0.1563、0.3125、0.625、1.25、

2.5 μM) were added in each well and incubated for two weeks. The medium was changed every 4 – 5 days, for a total of two weeks. The culture was stopped when visible colonies appeared on the plate. The culture solution was removed and the plate was rinsed with PBS buffer solution twice. The colony was fixed by adding 100% formaldehyde for 15 – 20 min (37 °C). Following natural drying, moderate 0.1% crystal violet was added for 15 – 20 min (37 °C). The residual dye was washed off with water and the plate was air-dried.

Reference

(1) Wang, J. M.; Wolf, R. M.; Caldwell, J. W.; Kollman, P. A.; Case, D. A. Development and testing of a general amber force field. *J. Comput. Chem.* **2004**, *25* (9), 1157-1174.

(2) *Gaussian 09*; Gaussian, Inc.: Wallingford, CT, USA, 2009. (accessed.

(3) Bayly, C. I.; Cieplak, P.; Cornell, W. D.; Kollman, P. A. A well-behaved electrostatic potential based method using charge restraints for deriving atomic charges: The resp model. *J. Phys. Chem.* **1993**, *97* (40), 10269-10280.

(4) Tian, C.; Kasavajhala, K.; Belfon, K. A. A.; Raguette, L.; Huang, H.; Migues, A. N.; Bickel, J.; Wang, Y. Z.; Pincay, J.; Wu, Q.; et al. Ff19sb: Amino-acid-specific protein backbone parameters trained against quantum mechanics energy surfaces in solution. *Journal of Chemical Theory and Computation* **2020**, *16* (1), 528-552.

(5) Waterhouse, A.; Bertoni, M.; Bienert, S.; Studer, G.; Tauriello, G.; Gumienny, R.; Heer, F. T.; de Beer, T. A. P.; Rempfer, C.; Bordoli, L.; et al. Swiss-model: Homology modelling of protein structures and complexes. *Nucleic Acids Res.* **2018**, *46* (W1), W296-W303.

(6) Guex, N.; Peitsch, M. C.; Schwede, T. Automated comparative protein structure modeling with swiss-model and swiss-pdbviewer: A historical perspective. *Electrophoresis* **2009**, *30*, S162-S173.

(7) Guex, N.; Peitsch, M. C. Swiss-model and the swiss-pdbviewer: An environment for comparative protein modeling. *Electrophoresis* **1997**, *18* (15), 2714-2723.

(8) Anandakrishnan, R.; Aguilar, B.; Onufriev, A. V. H++3.0: Automating pk prediction and the preparation of biomolecular structures for atomistic molecular modeling and simulations. *Nucleic Acids Res.* **2012**, *40* (W1), W537-W541.

(9) Myers, J.; Grothaus, G.; Narayanan, S.; Onufriev, A. A simple clustering algorithm can be accurate enough for use in calculations of pks in macromolecules. *Proteins* **2006**, *63* (4), 928-938.

(10) Gordon, J. C.; Myers, J. B.; Folta, T.; Shoja, V.; Heath, L. S.; Onufriev, A. H++: A server for estimating pk(a)s and adding missing hydrogens to macromolecules. *Nucleic Acids Res.* **2005**, *33*, W368-W371.

(11) Bashford, D.; Karplus, M. Pka of ionizable groups in proteins: Atomic detail from a continuum electrostatic model. *Biochemistry* **1990**, *29* (44), 10219-10225.

(12) Jorgensen, W. L.; Chandrasekhar, J.; Madura, J. D.; Impey, R. W.; Klein, M. L. Comparison of simple potential functions for simulating liquid water. *The Journal of Chemical Physics* **1983**, *79* (2), 926-935.

(13) Darden, T.; York, D.; Pedersen, L. Particle mesh ewald: An n · log(n) method for ewald sums in large systems. *The Journal of Chemical Physics* **1993**, *98* (12), 10089-10092.

(14) Ryckaert, J.-P.; Ciccotti, G.; Berendsen, H. J. C. Numerical integration of the cartesian equations of motion of a system with constraints: Molecular dynamics of n-alkanes. *Journal of Computational Physics* **1977**, *23* (3), 327-341.

(15) Feller, S. E.; Zhang, Y. H.; Pastor, R. W.; Brooks, B. R. Constant pressure molecular dynamics simulation: The langevin piston method. *J. Chem. Phys.* **1995**, *103* (11), 4613-4621.

(16) Martyna, G. J.; Tobias, D. J.; Klein, M. L. Constant pressure molecular dynamics algorithms. *J. Chem. Phys.* **1994**, *101* (5), 4177-4189.

(17) Berendsen, H. J. C.; Postma, J. P. M.; Vangunsteren, W. F.; Dinola, A.; Haak, J. R. Molecular dynamics with coupling to an external bath. *J. Chem. Phys.* **1984**, *81* (8), 3684-3690.

(18) *Amber 2020, university of california, san francisco*; 2020. (accessed.

(19) Roe, D. R.; Cheatham, T. E. Parallelization of cpptraj enables large scale analysis of molecular dynamics trajectory data. *J. Comput. Chem.* **2018**, *39* (25), 2110-2117.

(20) Roe, D. R.; Cheatham, T. E. Ptraj and cpptraj: Software for processing and analysis of molecular dynamics trajectory data. *Journal of Chemical Theory and Computation* **2013**, *9* (7), 3084-3095.

(21) Srinivasan, J.; Cheatham, T. E.; Cieplak, P.; Kollman, P. A.; Case, D. A. Continuum solvent studies of the stability of DNA, rna, and phosphoramidate - DNA helices. *J. Am. Chem. Soc.* **1998**, *120* (37), 9401-9409.

(22) Lee, M. S.; Salsbury, F. R.; Olson, M. A. An efficient hybrid explicit/implicit solvent method for biomolecular simulations. *J. Comput. Chem.* **2004**, *25* (16), 1967-1978.

(23) Shi, M.; Xu, D. Molecular dynamics investigations suggest a non-specific recognition strategy of 14-3-3σ protein by tweezer: Implication for the inhibition mechanism. *Front. Chem.* **2019**, *7* (237).

(24) Wang, J. Y.; Chen, Q.; Wang, M.; Zhong, C. The opening/closure of the p-loop and hinge of bcr-abl1 decodes the low/high bioactivities of dasatinib and axitinib. *Phys. Chem. Chem. Phys.* **2017**, *19* (33), 22444-22453.

(25) Tse, A.; Verkhivker, G. M. Molecular dynamics simulations and structural network analysis of c-abl and c-src kinase core proteins: Capturing allosteric mechanisms and communication pathways from residue centrality. *J. Chem Inf. Model.* **2015**, *55* (8), 1645-1662.

(26) Honig, B.; Nicholls, A. Classical electrostatics in biology and chemistry. *Science* **1995**, *268* (5214), 1144-1149.

(27) Genheden, S.; Ryde, U. The mm/pbsa and mm/gbsa methods to estimate ligand-binding affinities. *Expert. Opin. Drug Discov.* **2015**, *10* (5), 449-461.

(28) Onufriev, A. V.; Case, D. A. Generalized born implicit solvent models for biomolecules. In *Annual review of biophysics, vol 48*, Dill, K. A. Ed.; Annual review of biophysics, Vol. 48; Annual Reviews, 2019; pp 275-296.

(29) Weiser, J.; Shenkin, P. S.; Still, W. C. Approximate atomic surfaces from linear combinations of pairwise overlaps (lcpo). *J. Comput. Chem.* **1999**, *20* (2), 217-230.

(30) Still, W. C.; Tempczyk, A.; Hawley, R. C.; Hendrickson, T. Semianalytical treatment of solvation for molecular mechanics and dynamics. *J. Am. Chem. Soc.* **1990**, *112* (16), 6127-6129.

(31) Srinivasan, J.; Trevathan, M. W.; Beroza, P.; Case, D. A. Application of a pairwise generalized born model to proteins and nucleic acids: Inclusion of salt effects. *Theor. Chem. Acc.* **1999**, *101* (6), 426-434.

(32) Nguyen, H.; Roe, D. R.; Simmerling, C. Improved generalized born solvent model parameters for protein simulations. *Journal of Chemical Theory and Computation* **2013**, *9* (4), 2020-2034.

(33) Nguyen, H.; Perez, A.; Bermeo, S.; Simmerling, C. Refinement of generalized born implicit solvation parameters for nucleic acids and their complexes with proteins. *Journal of Chemical Theory and Computation* **2015**, *11* (8), 3714-3728.

(34) Gao, P. C.; Li, Z. L. Computation of the boltzmann entropy of a landscape: A review and a generalization. *Landsc. Ecol.* **2019**, *34* (9), 2183-2196.

(35) Miller, B. R.; McGee, T. D.; Swails, J. M.; Homeyer, N.; Gohlke, H.; Roitberg, A. E. Mmpbsa.Py: An efficient program for end-state free energy calculations. *Journal of Chemical Theory and Computation* **2012**, *8* (9), 3314-3321.

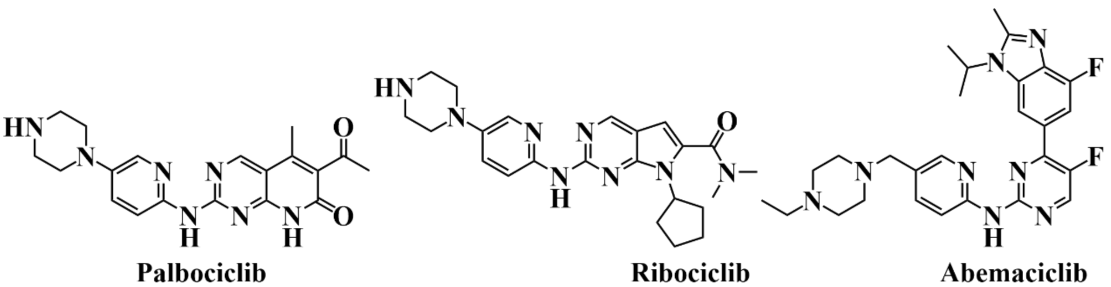

Palbociclib **Ribociclib** **Abemaciclib**

Figure S1. Structures of CDK4/6 inhibitors.

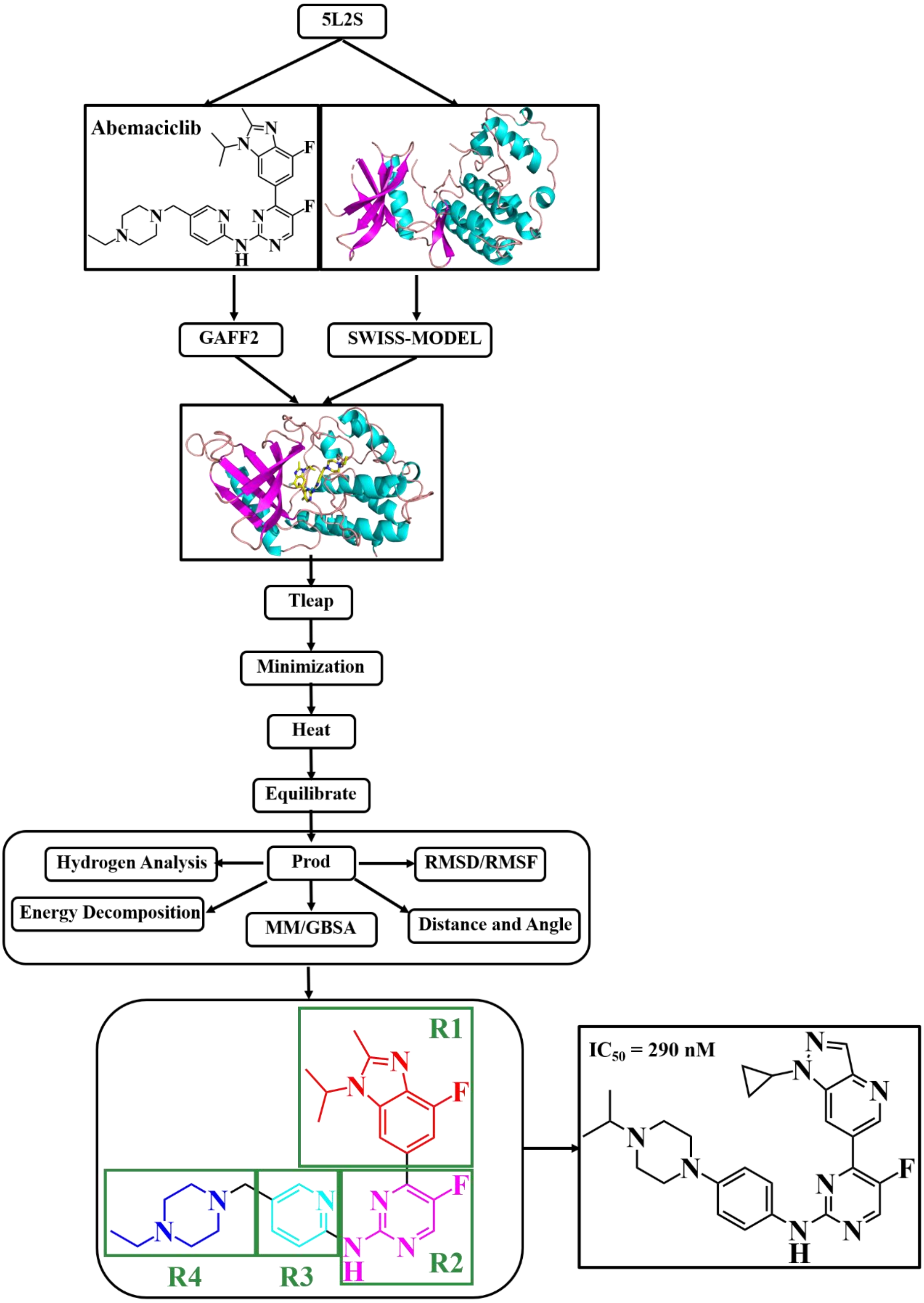

Figure S2. Methods used in this work.

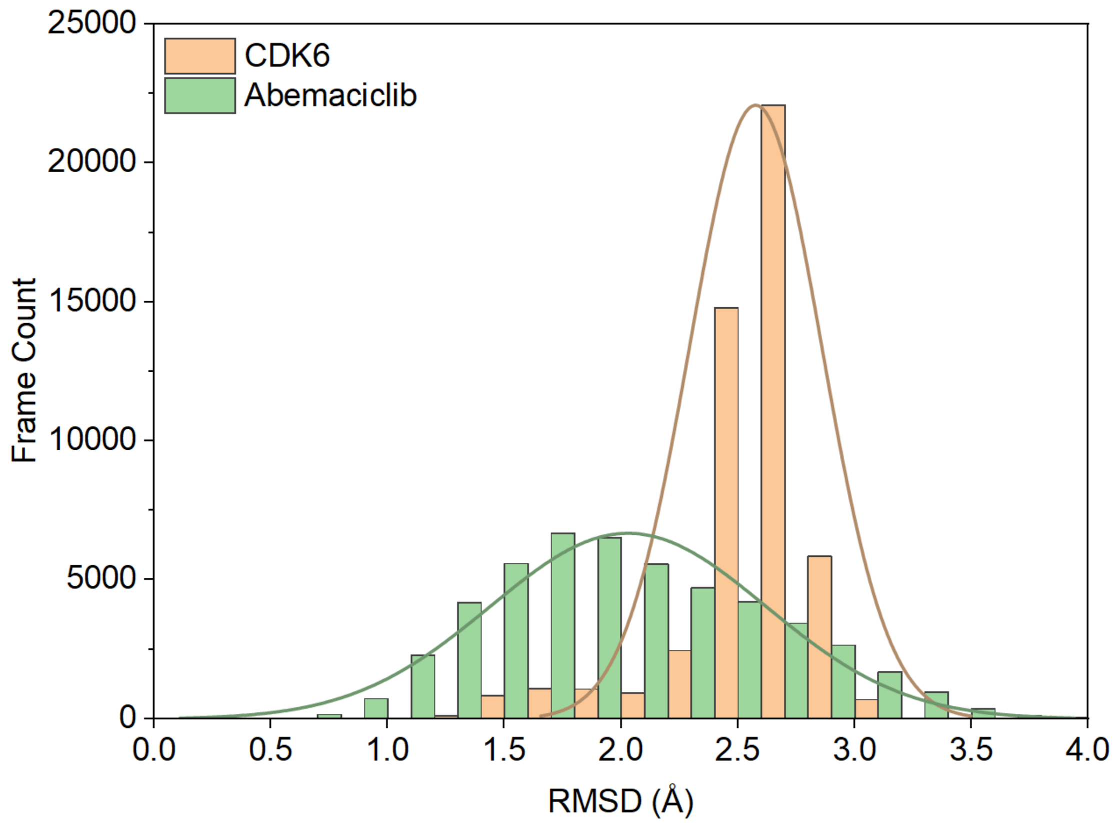

Figure S3. Root mean square deviation (RMSD) value of heavy atoms of backbone for human CDK6 and that of abemaciclib from 500 ns MD simulation for abemaciclib/CDK6 system.

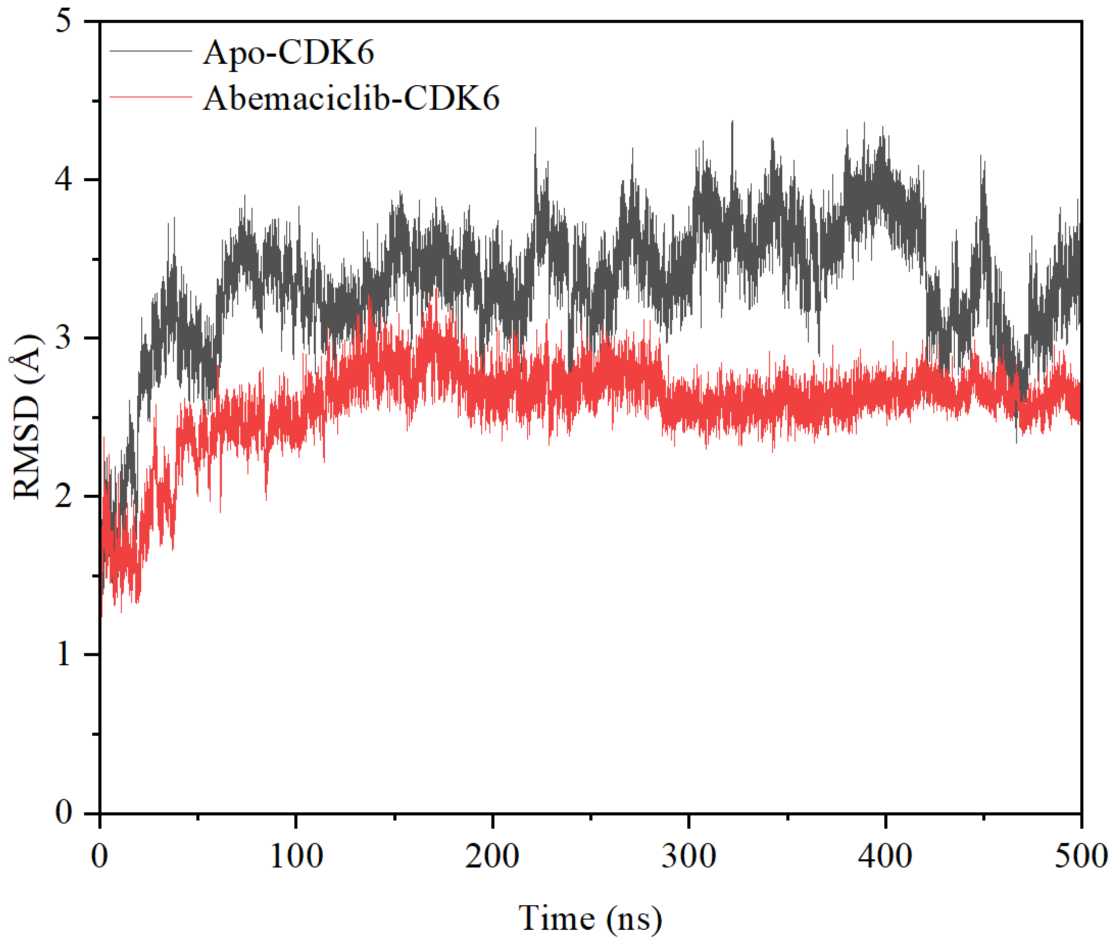

Figure S4. Root mean square deviation (RMSD) value of heavy atoms of backbone for human CDK6 and along 500 ns MD simulation for abemaciclib/CDK6 and Apo-CDK6 systems.

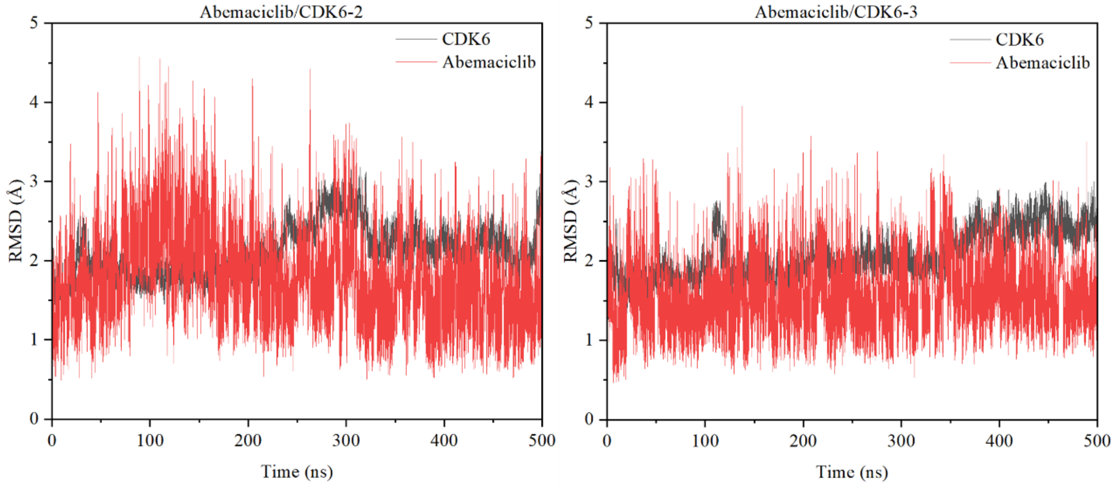

Figure S5. Root mean square deviation (RMSD) value of heavy atoms of backbone for human CDK6 and that of abemaciclib along with 500 ns MD simulation for two replicated abemaciclib/CDK6 systems (abemaciclib/CDK6-2 and abemaciclib/CDK6-3 systems).

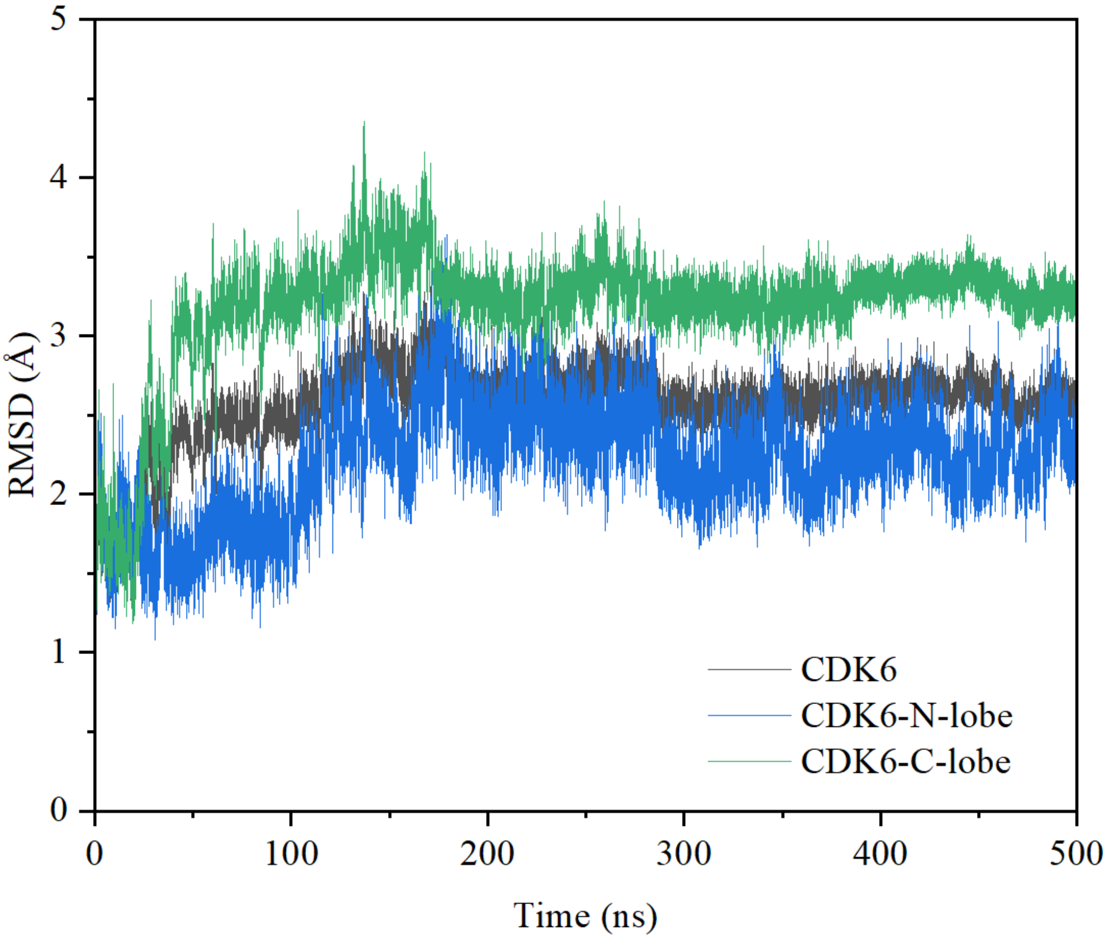

Figure S6. Root mean square deviation (RMSD) value of heavy atoms of backbone for different region (N-lobe and C-lobe of kinase domain for human CDK6) of human CDK6 along with 500 ns MD simulation for abemaciclib/CDK6 system.

The residues for N-lobe (labeled as CDK6-N-lobe) and C-lobe (labeled as CDK6-C-lobe) were 11-98 and 104−300, respectively. Meanwhile, the human CDK6 (resides:11-300applied in this work) was labeled as CDK6 in the figure.

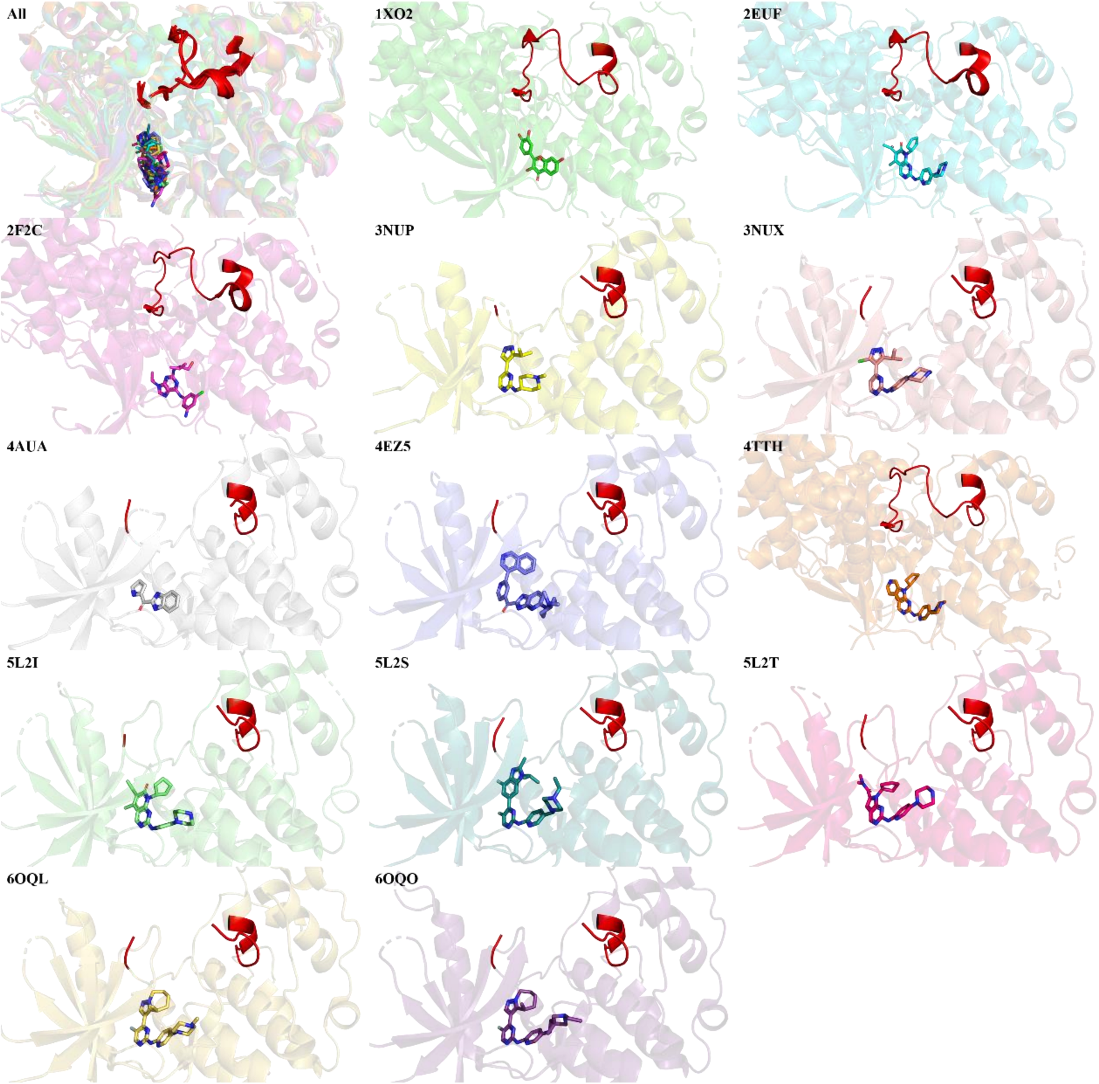

Figure S7. Crystal structures of inhibitor/CDK6 complex with T-loop.

The T-loop region shown with red cartoon style. The inhibitor shown with stick style. Those structures were labeled as PDB ID.

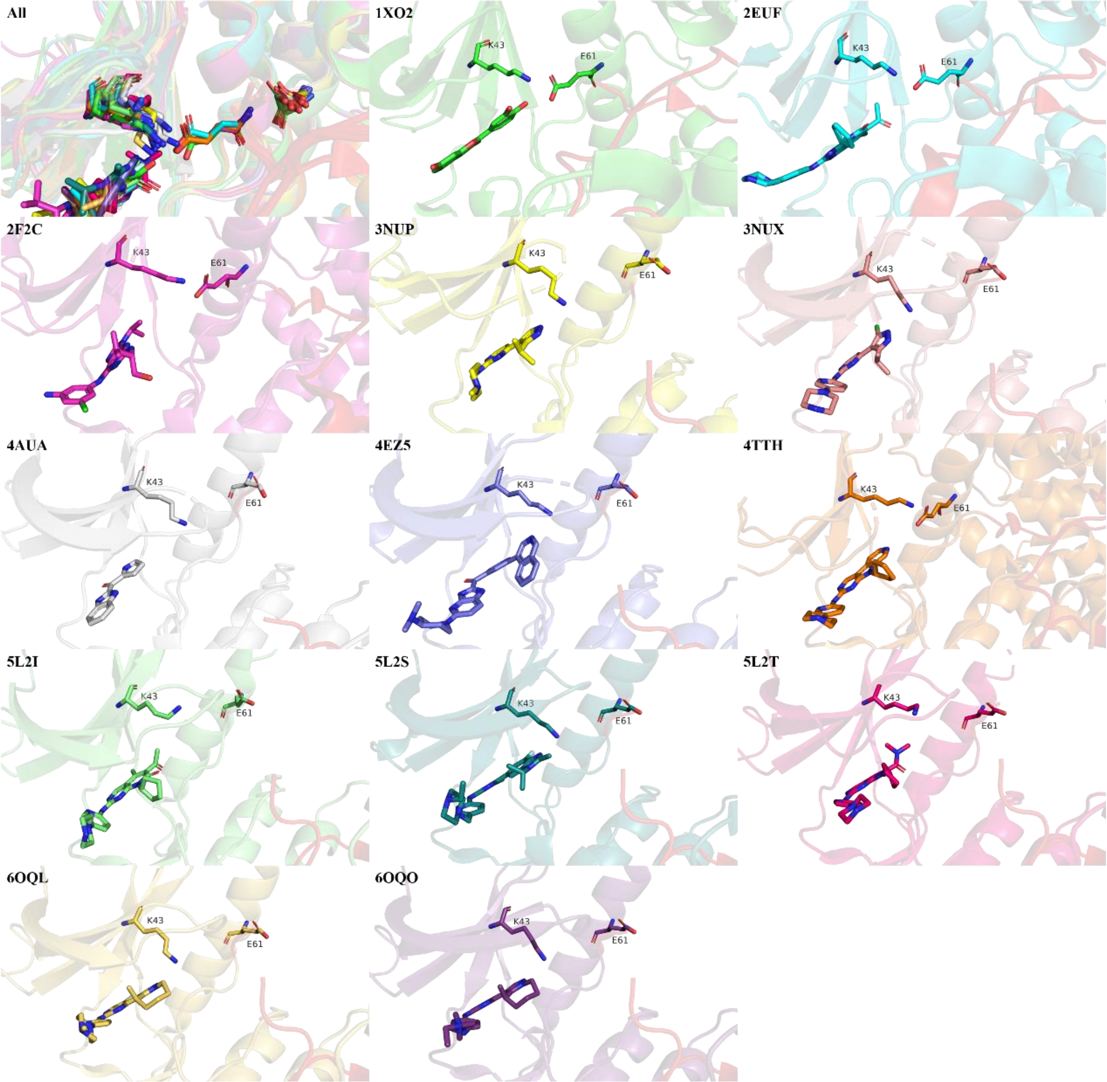

Figure S8. Crystal structures of inhibitor/CDK6 complex with K43-E61 salt bridge. The salt bridge residue K43 and E61 shown with stick style. The inhibitor shown with stick style. Those structures were labeled as PDB ID.

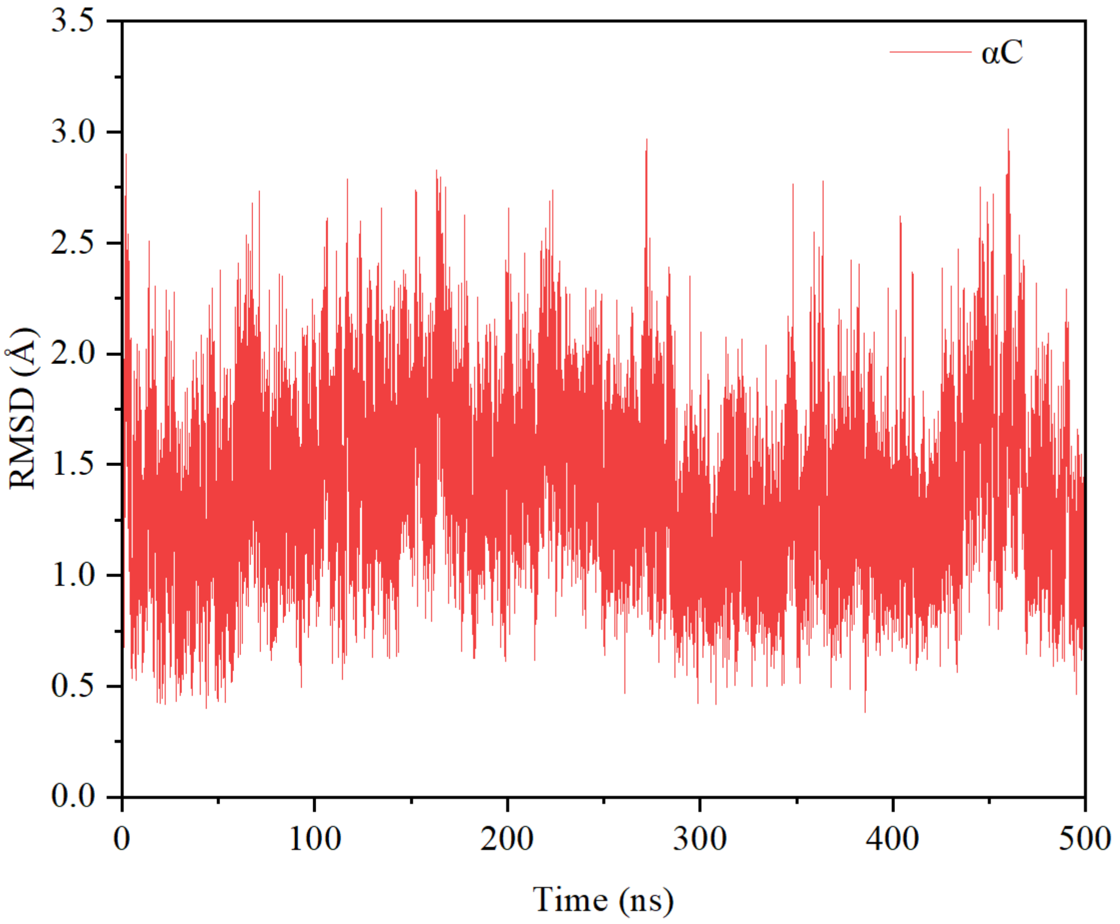

Figure S9. Root mean square deviation (RMSD) value of heavy atoms of backbone for αC domain for human CDK6 along with 500 ns MD simulation for abemaciclib/CDK6 system.

The residues for αC were from 56 to 70.

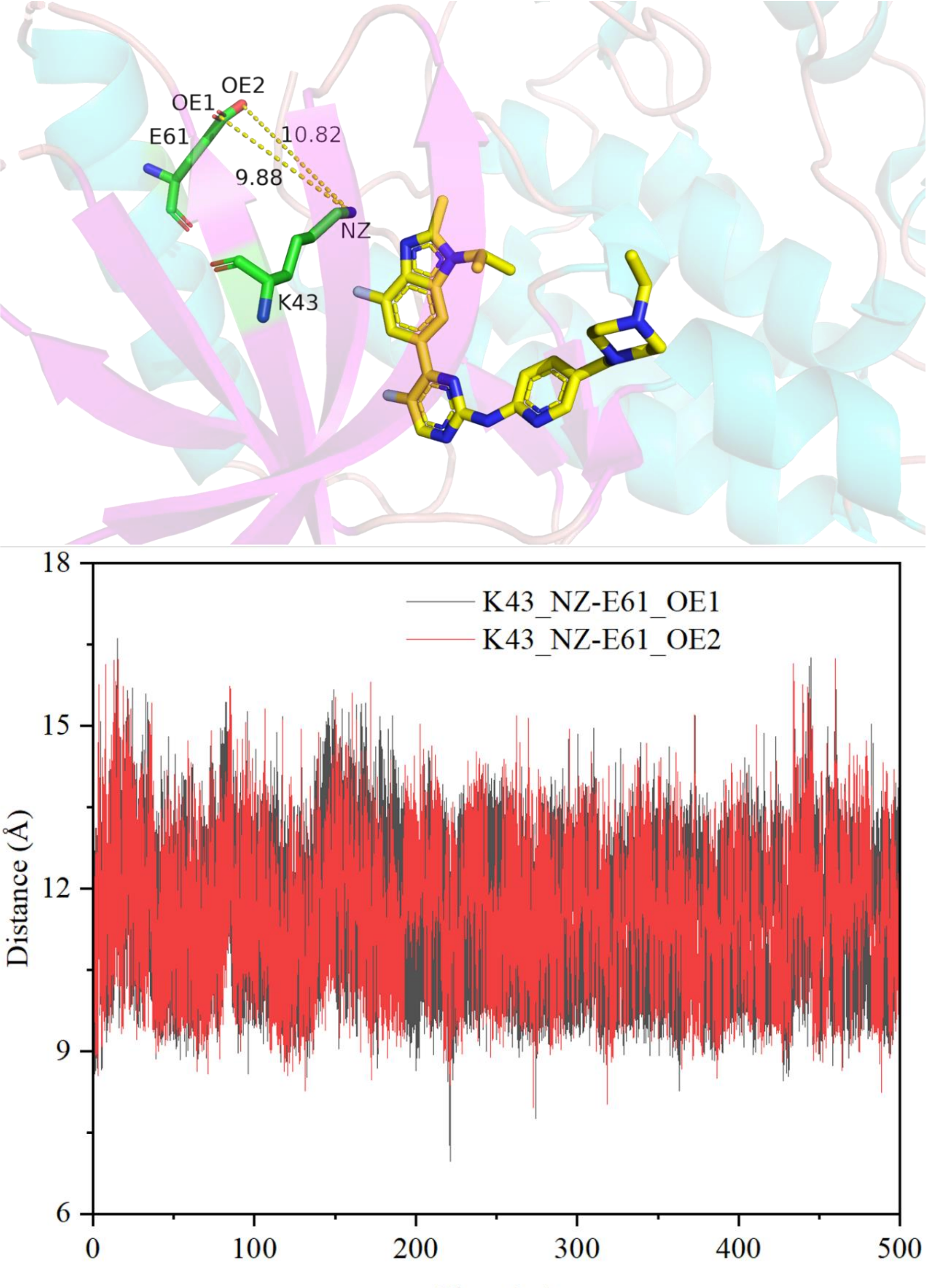

Figure 10. Distance between K43 and E61 along with the simulation time.

Distance between K43 and E61 was defined as NZ atom of K43 with atom OE1 or OE2 of E61 for the human CDK6.

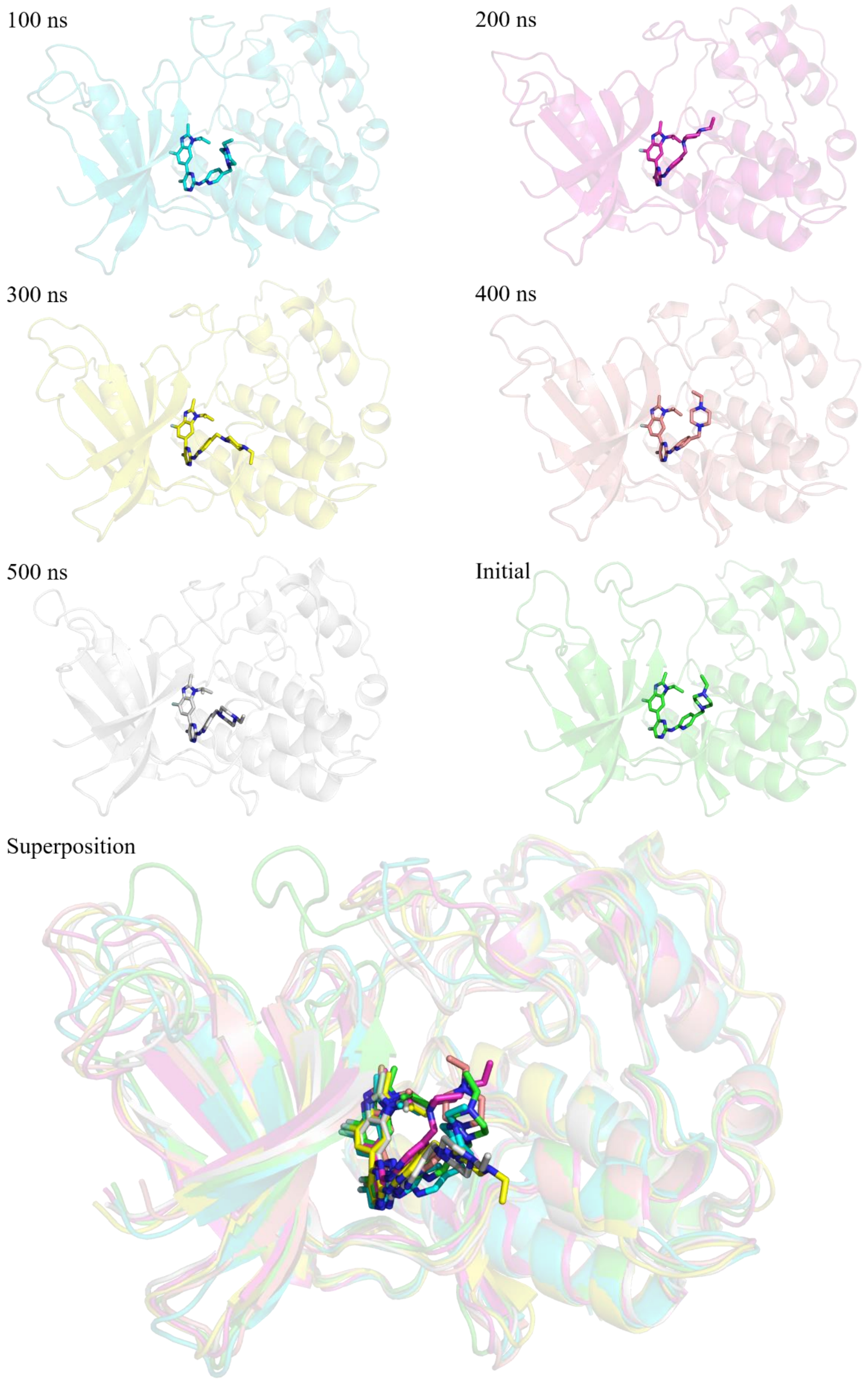

Figure S11. Snapshots of the abemaciclib/CDK6 along the dynamic simulation time for 100, 200, 300, 400, and 500 ns.

For clarity, the water molecules have been removed. The inhibitor abemaciclib is plotted using stick style, while cartoon style for human CDK6.

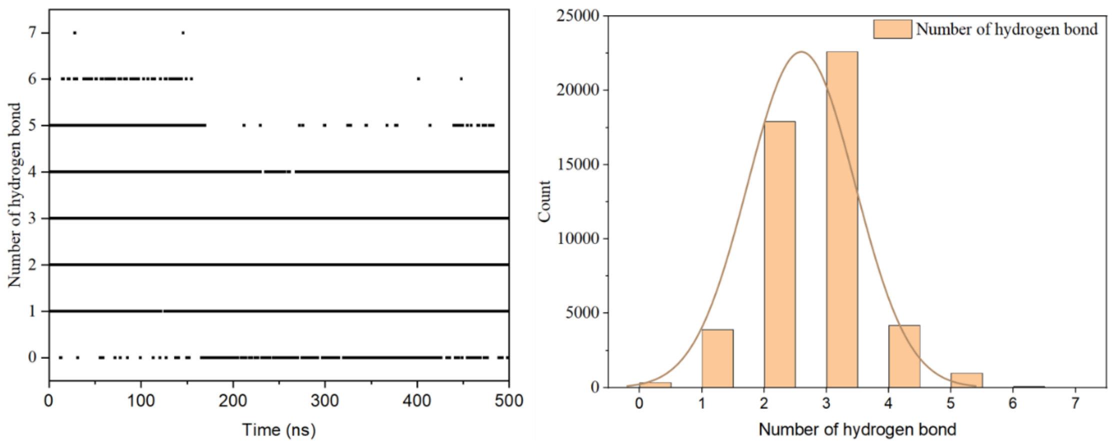

Figure S12. Hydrogen bond analysis for abemaciclib/CDK6 system.

Hydrogen bond is defined as the distance between the acceptor and donor atoms (< 3.5 Å), with an internal angle between the H-acceptor and H-donor (> 120°). The total frames for hydrogen bond analysis are 50000 which extracted from the 500 ns simulation.

Hydrogen Bond	Occupancy
Abe@N3…V101@N	85.31%
H100@ND1…Abe@N8	80.27%
V101@O…Abe@N8	8.35%
Abe@N4…K43@NZ	20.74%

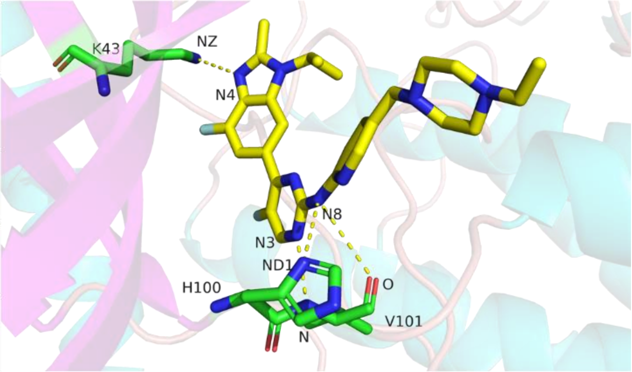

Figure S13. Hydrogen bond analysis for the last 200 ns simulation of abemaciclib/CDK6 system.

A hydrogen bond is formed when the distance between the acceptor and donor atoms is shorter than 3.5 Å, and the internal angle of the acceptor···H-donor is larger than 120°. The occupancy was calculated for the last 200 ns simulation.

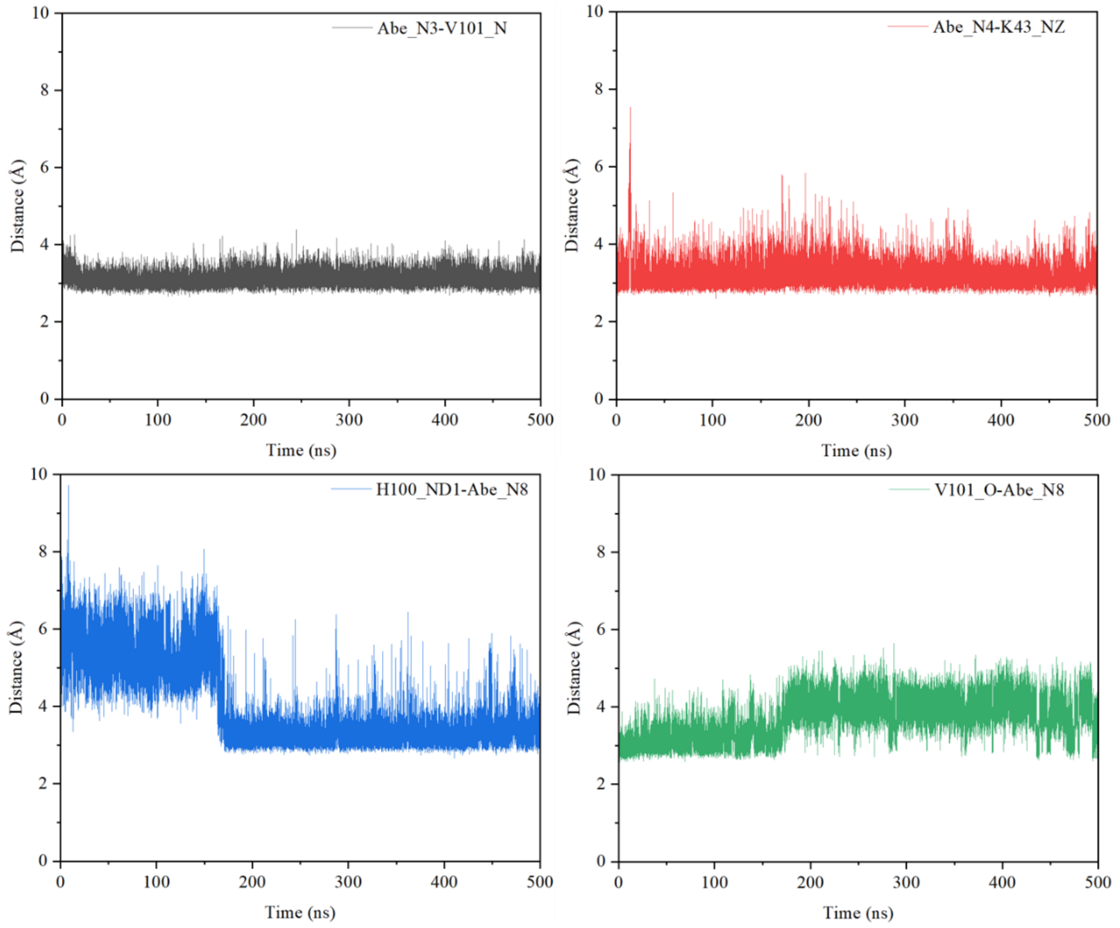

Figure S14. Distance for the hydrogen bonds between abemaciclib and hinge loop of human CDK6 in abemaciclib/CDK6 system.

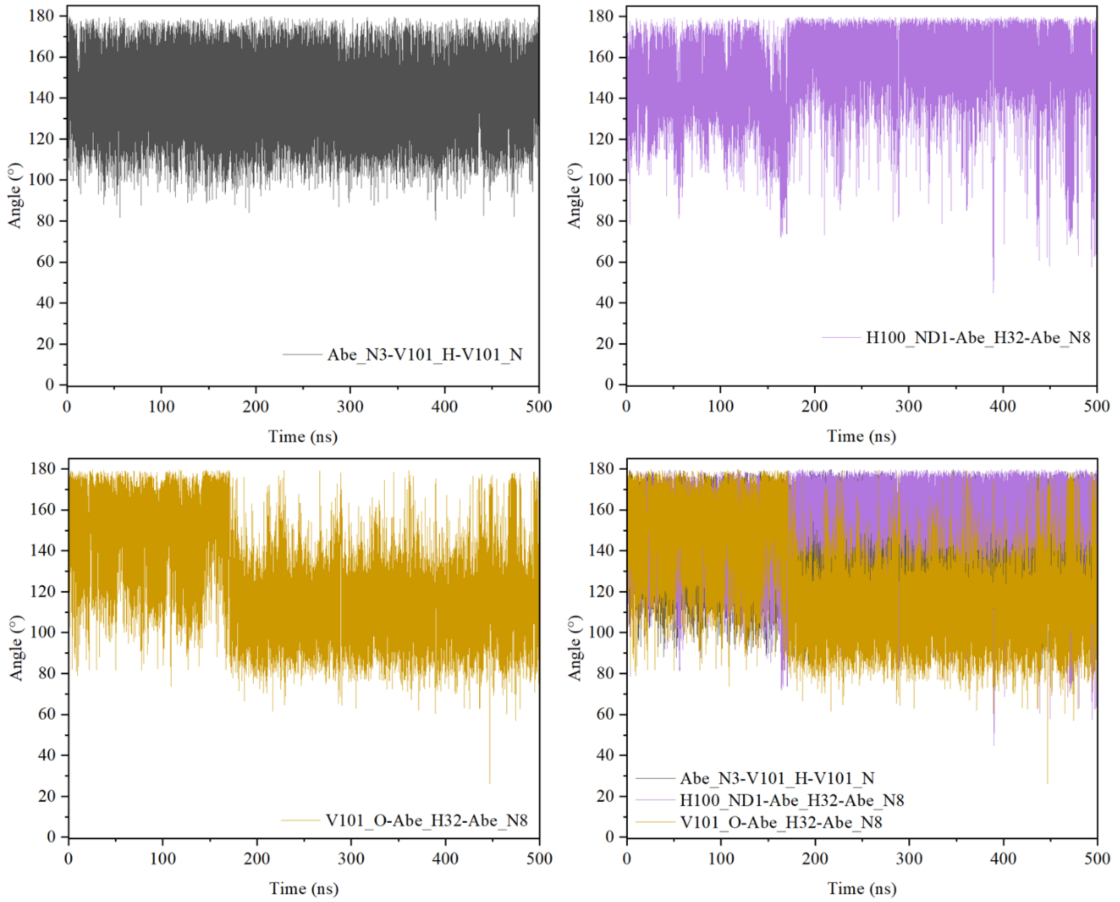

Figure S15. Angle for the hydrogen bonds between abemaciclib and hinge loop of human CDK6 in abemaciclib/CDK6 system.

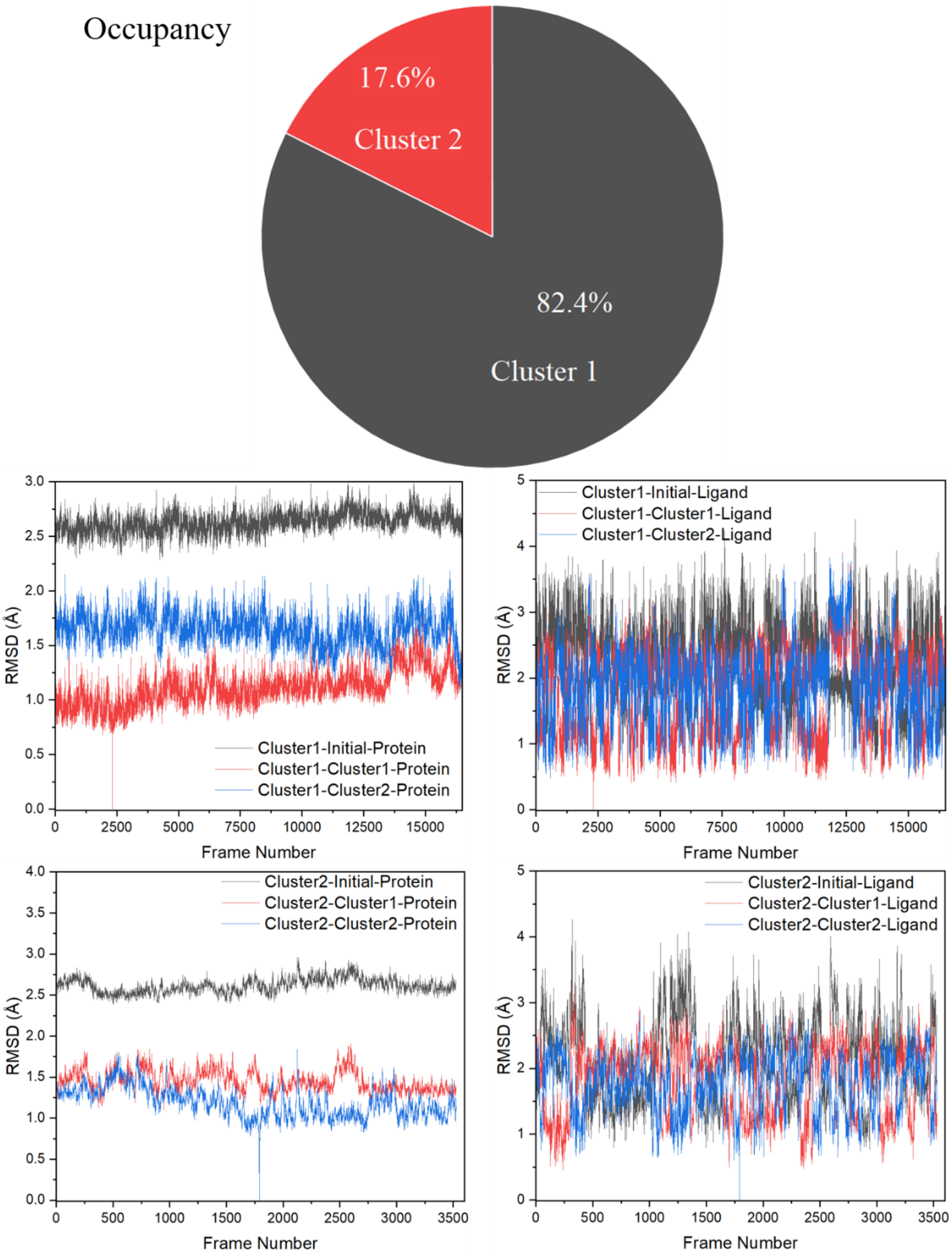

Figure S16. Cluster analysis results for the last 200 ns simulation.

Cluster analysis was based on the heavy atoms of abemaciclib/CDK6 complex to generate some clusters with epsilon = 2.0 Å. There are 16473 and 3527 frames for Cluster 1 and 2, respectively. The RMSD was calculated for frames in Cluster 1 (or Cluster 2) compared with initial frame, centroid frame of Cluster 1 (Cluster1) and Cluster 2 (Cluster2), respectively.

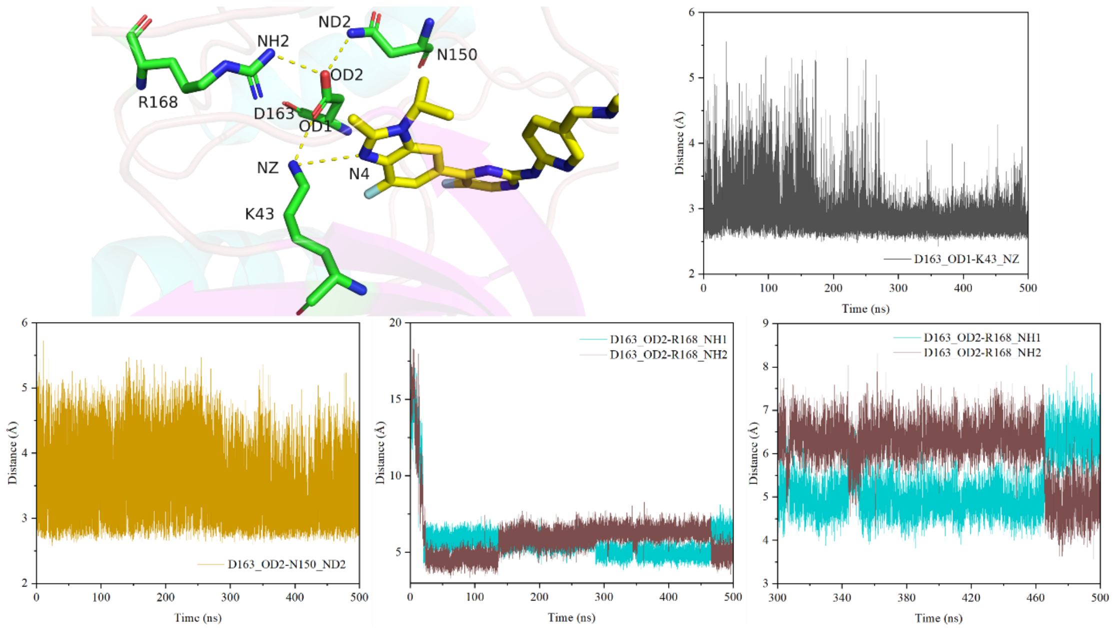

Figure S17. Hydrogen bond network for D163.

Hydrogen bond network formed among abemaciclib, K43, N150, D163, R168 along with 500 ns simulation.

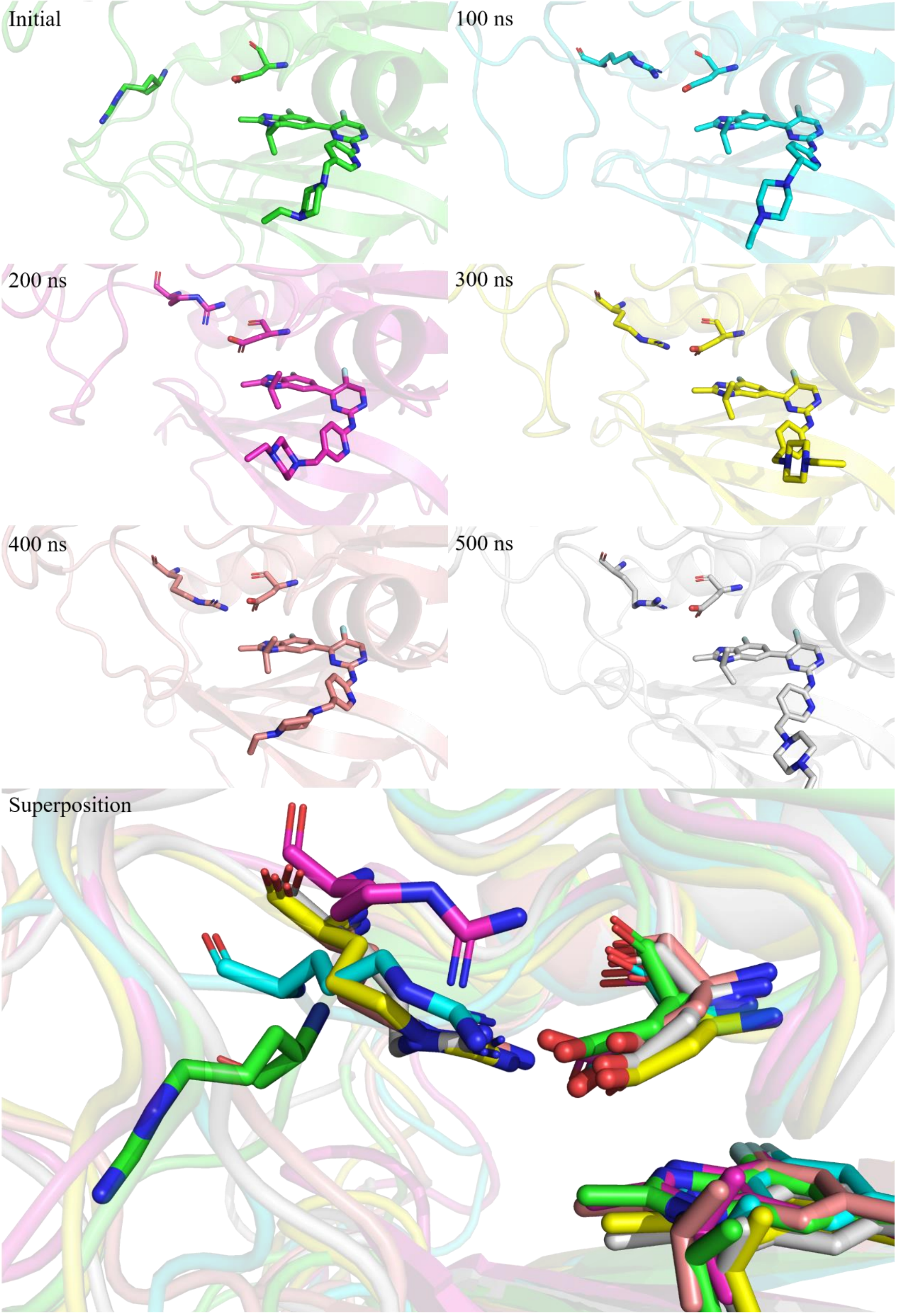

Figure S18. Conformation of D163 and R168 of the abemaciclib/CDK6 along the dynamic simulation time for 100, 200, 300, 400, and 500 ns.

For clarity, the water molecules have been removed. The inhibitor abemaciclib is plotted using stick style, while cartoon style for human CDK6.

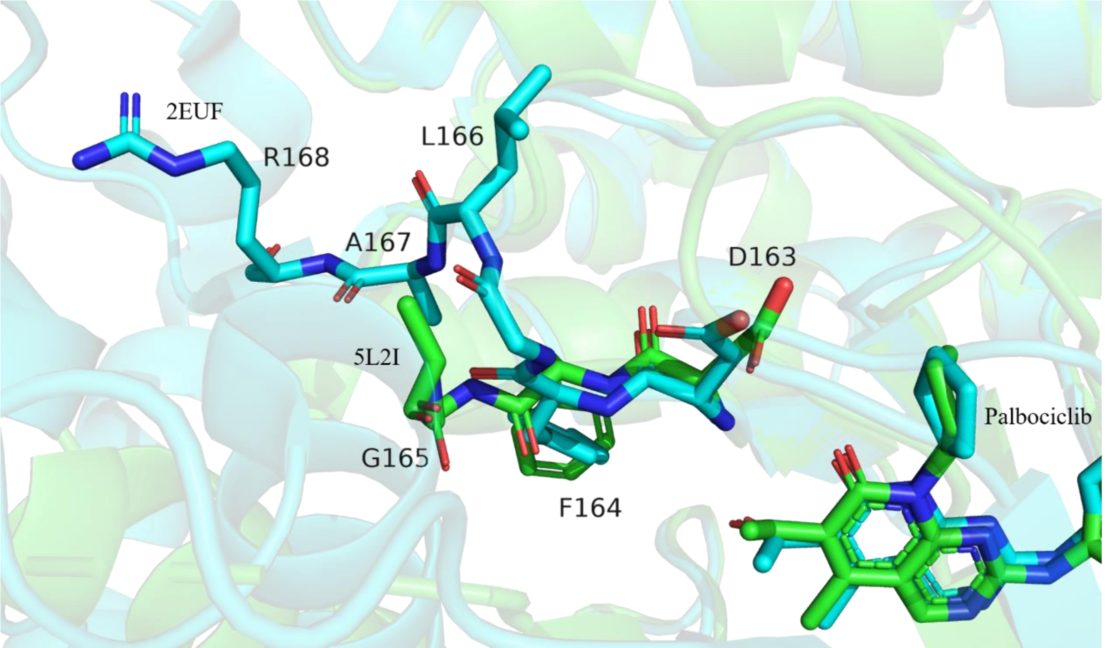

Figure S19. Palbociclib binding with human CDK6.

The crystal structures for palbociclib bound with human CDK6 were obtained the Protein Database Bank with 2EUF (PDB ID) and 5L2I. The ^{163}DFGLAR^{168} and palbociclib were shown with stick style and human CDK6 with cartoon style.

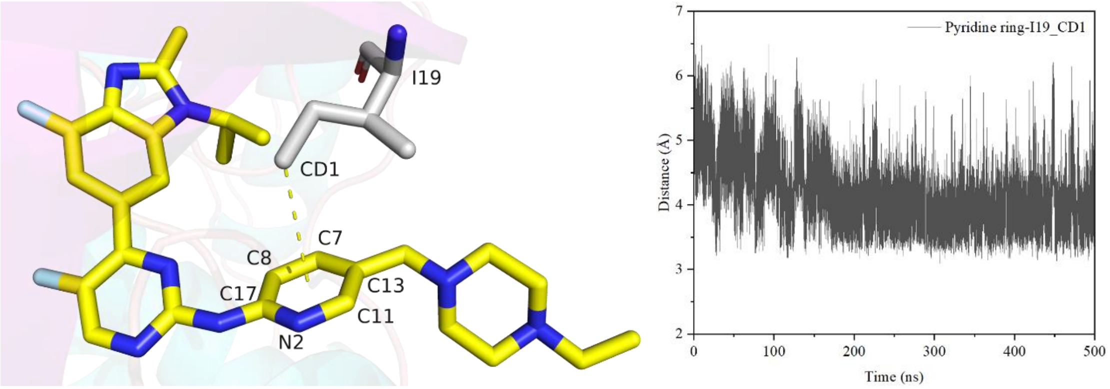

Figure S20. Distance between pyridine ring of abemaciclib and side chain of I19. The distance between pyridine ring of abemaciclib and side chain of I19 was defined with the center of pyridine ring (C8, C7, C13, C11, N2, and C17) of abemaciclib and CD1 of I19.

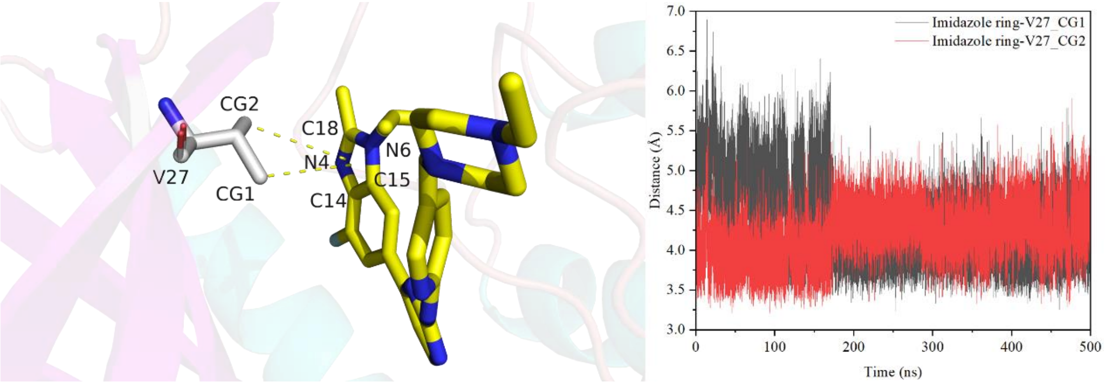

Figure S21. Distance between V27 and imidazole ring of abemaciclib along 500 ns simulation.

The distance was calculated between the CG1/CG2 of V27 and the center of imidazole ring (C14, C15, N6, C18, and N4) of abemaciclib along the 500 ns molecular dynamics simulation for abemaciclib/CDK6 complex system.

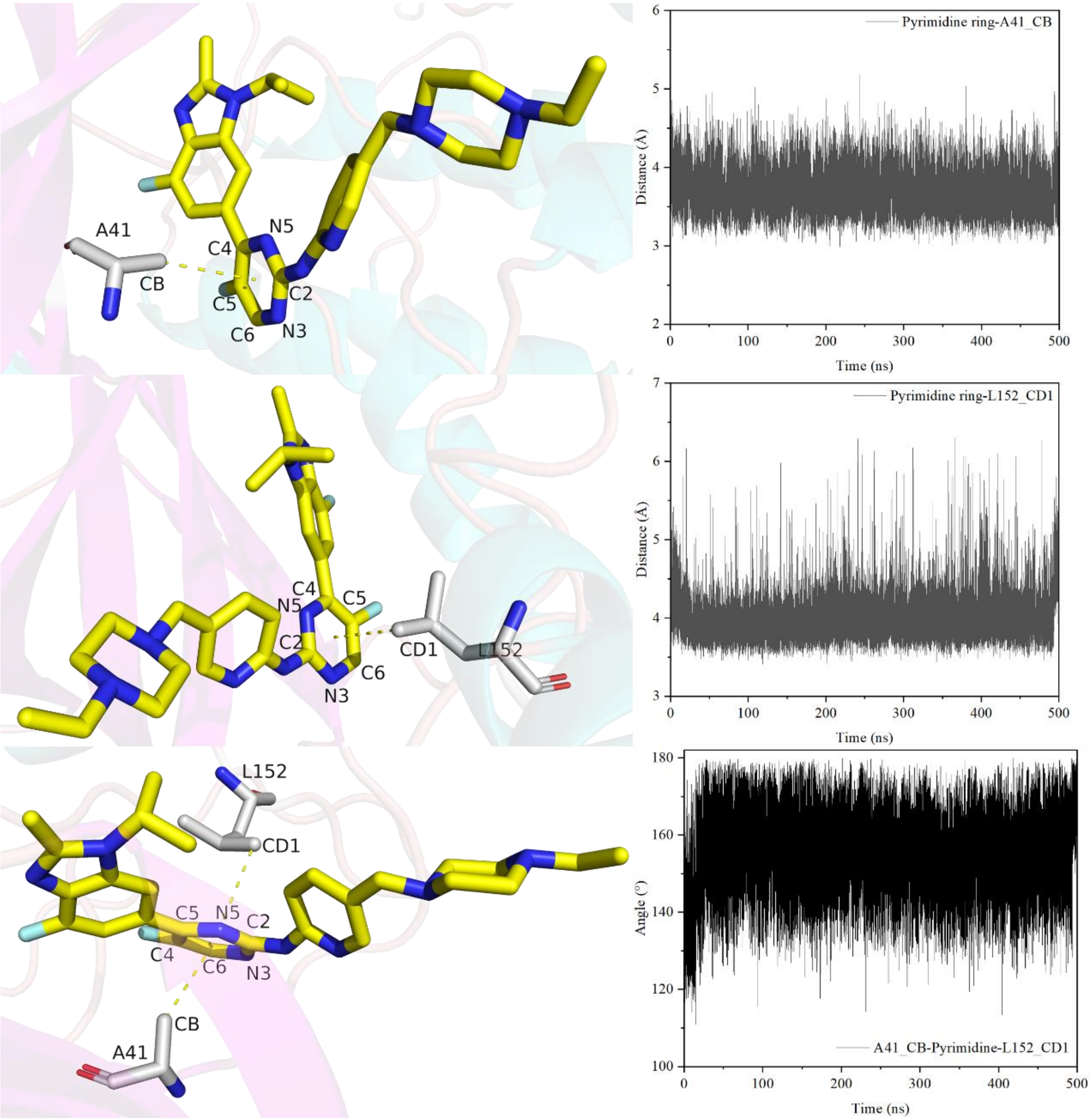

Figure S22. Distance and angle for A41, L152 and pyrimidine ring of abemaciclib for abemaciclib/CDK6 system.

The distance was calculated between the CB of A41 or CD1 of L152 and the center of pyrimidine ring (C6, N3, C2, N5, C5, and C4) of abemaciclib along the 500 ns molecular dynamics simulation for abemaciclib/CDK6 complex system.

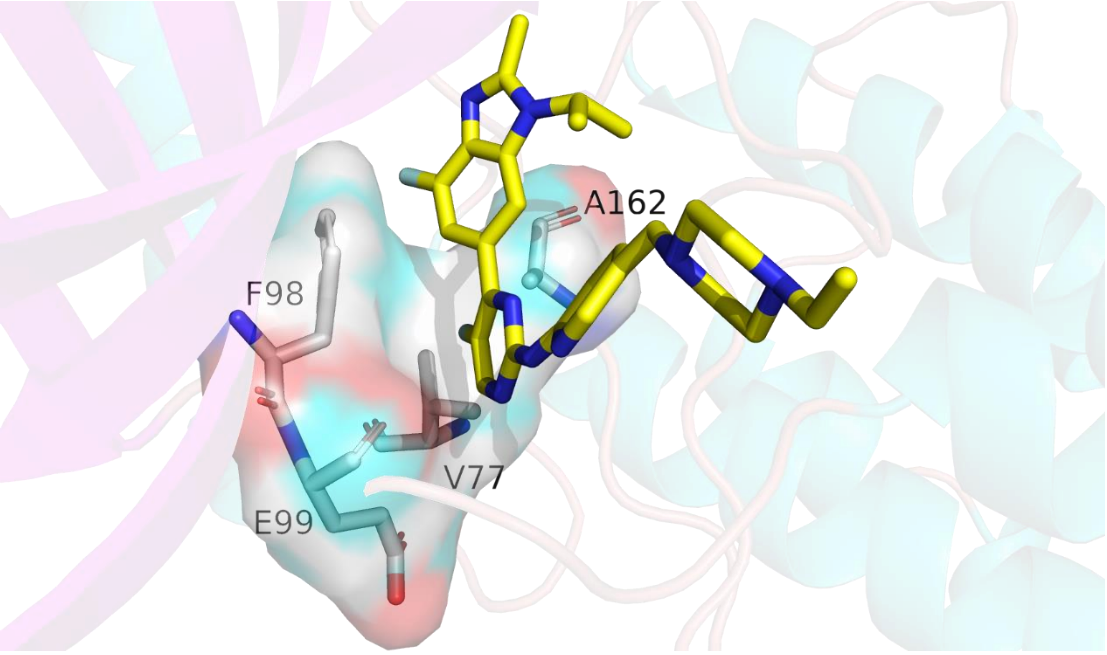

Figure S23. Interaction between abemaciclib and hydrophobic cavity of CDK6.

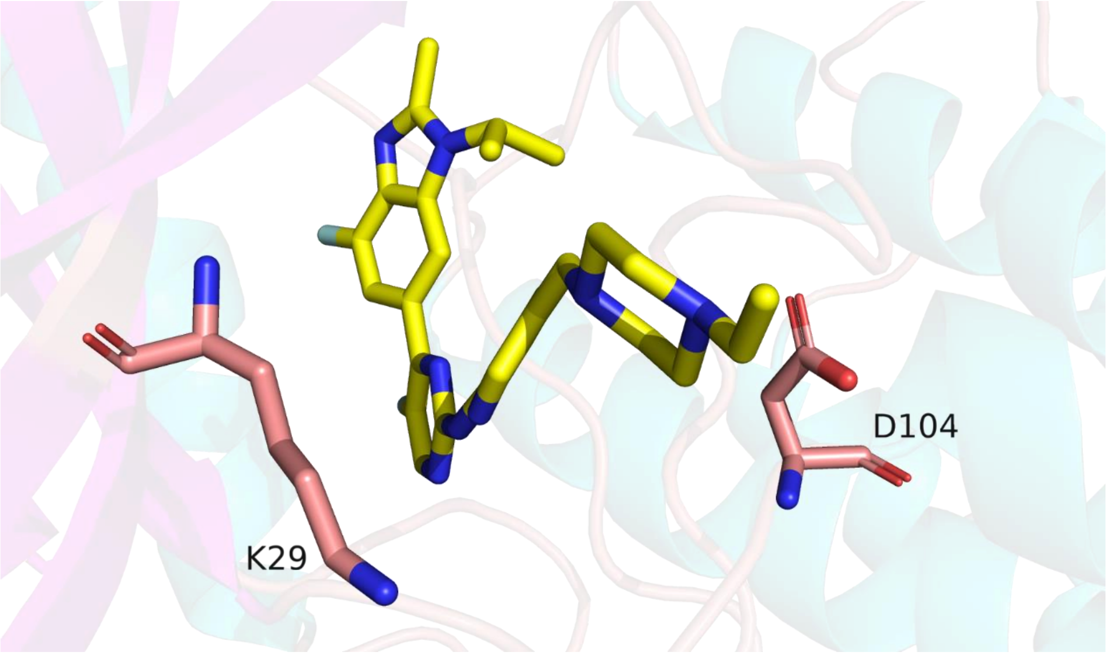

Figure S24. Piperazine group of abemaciclib interaction with K29/D104 of human CDK6.

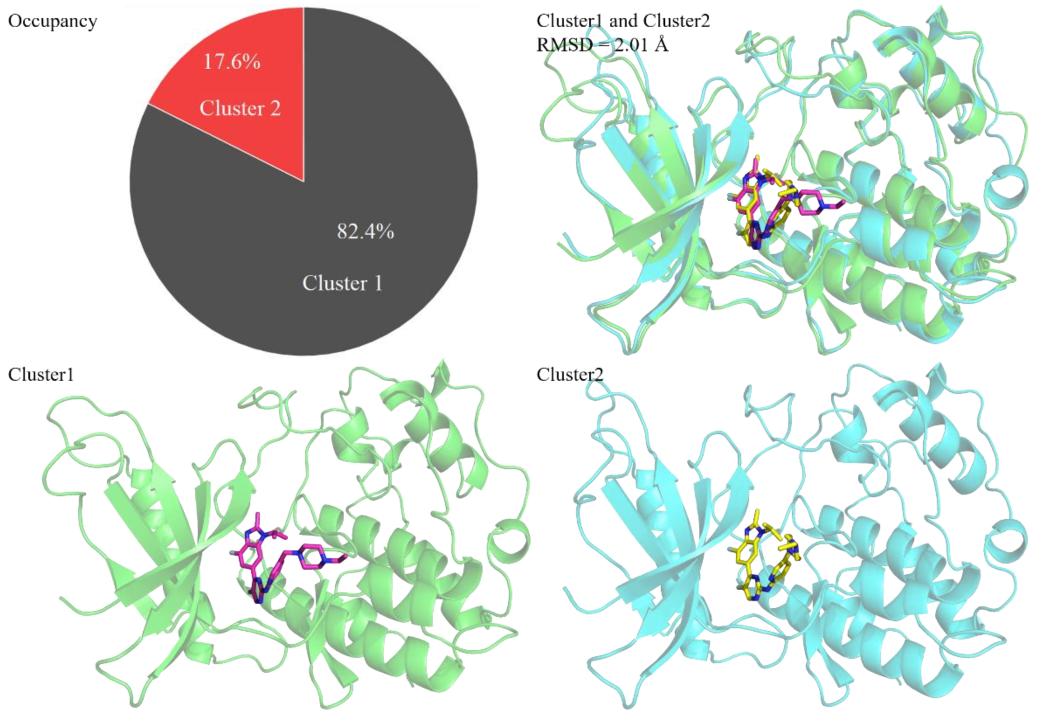

Figure S25. Cluster analysis results for the last 200 ns simulation.

Cluster analysis was based on the heavy atoms of abemaciclib/CDK6 complex to generate some clusters with epsilon = 2.0 Å.

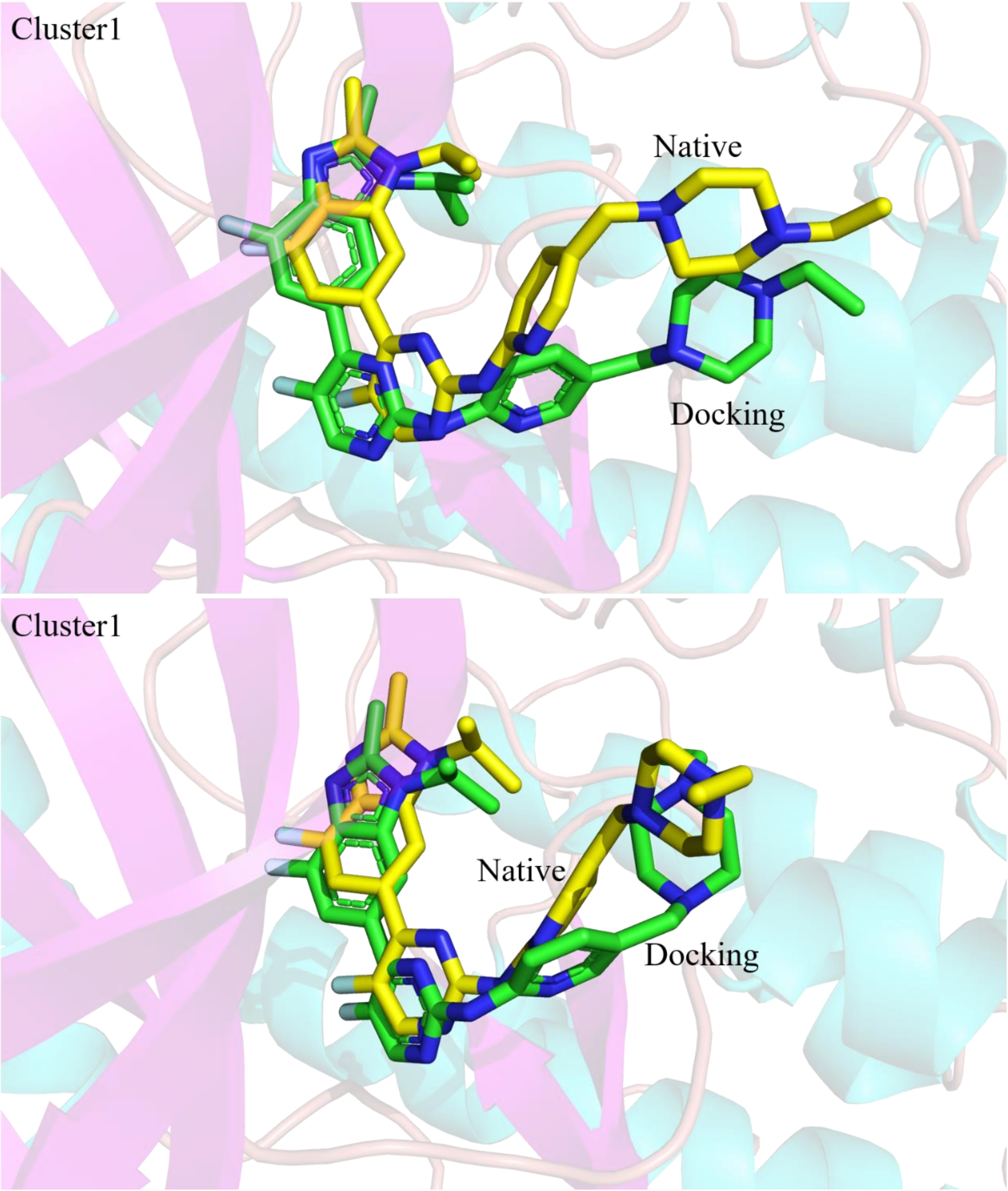

Figure S26. Re-dock results for the selected two abemaciclib/CDK6 representative models.

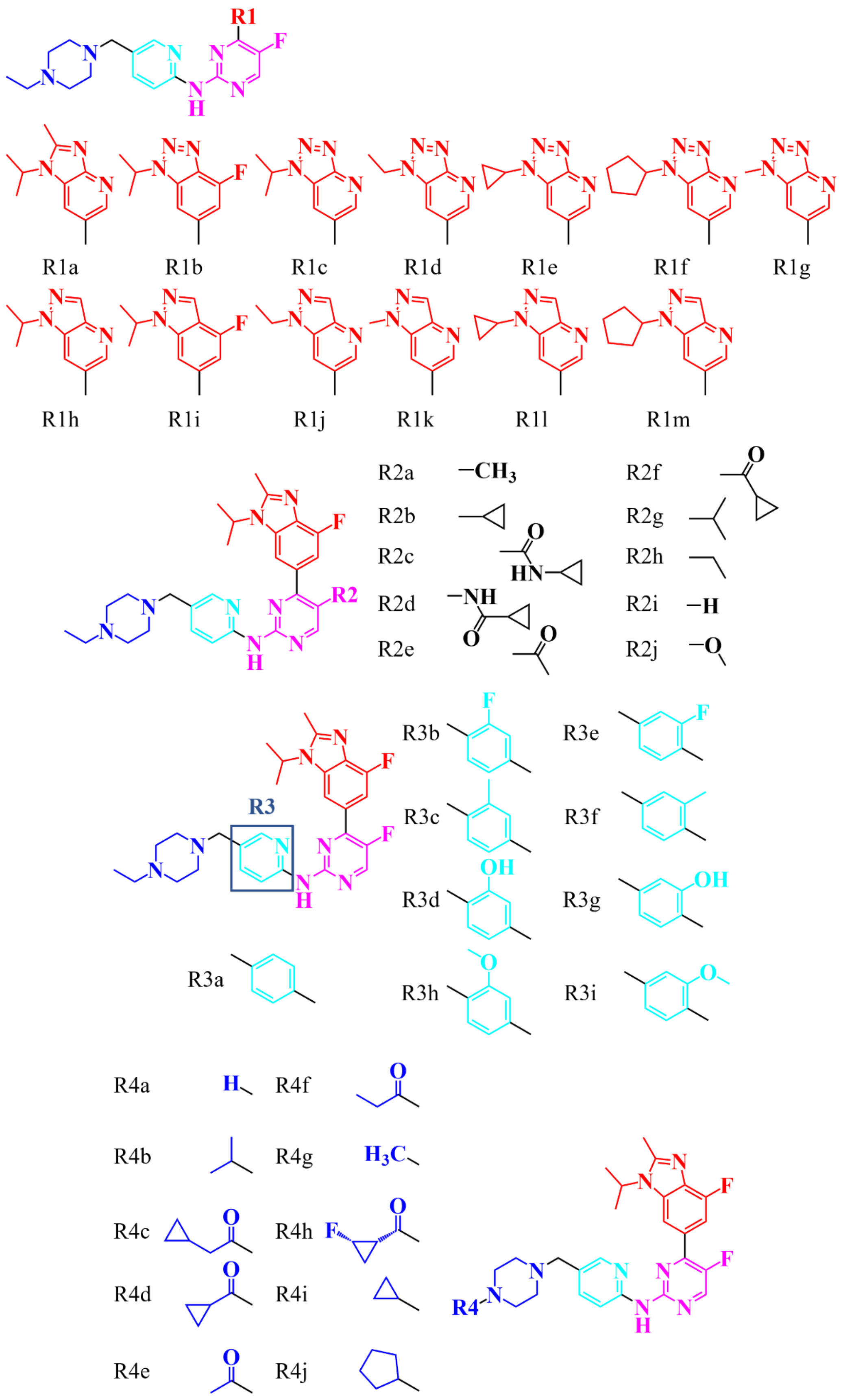

Figure S27. Designed novel CDK6 inhibitors based on abemaciclib with one region.

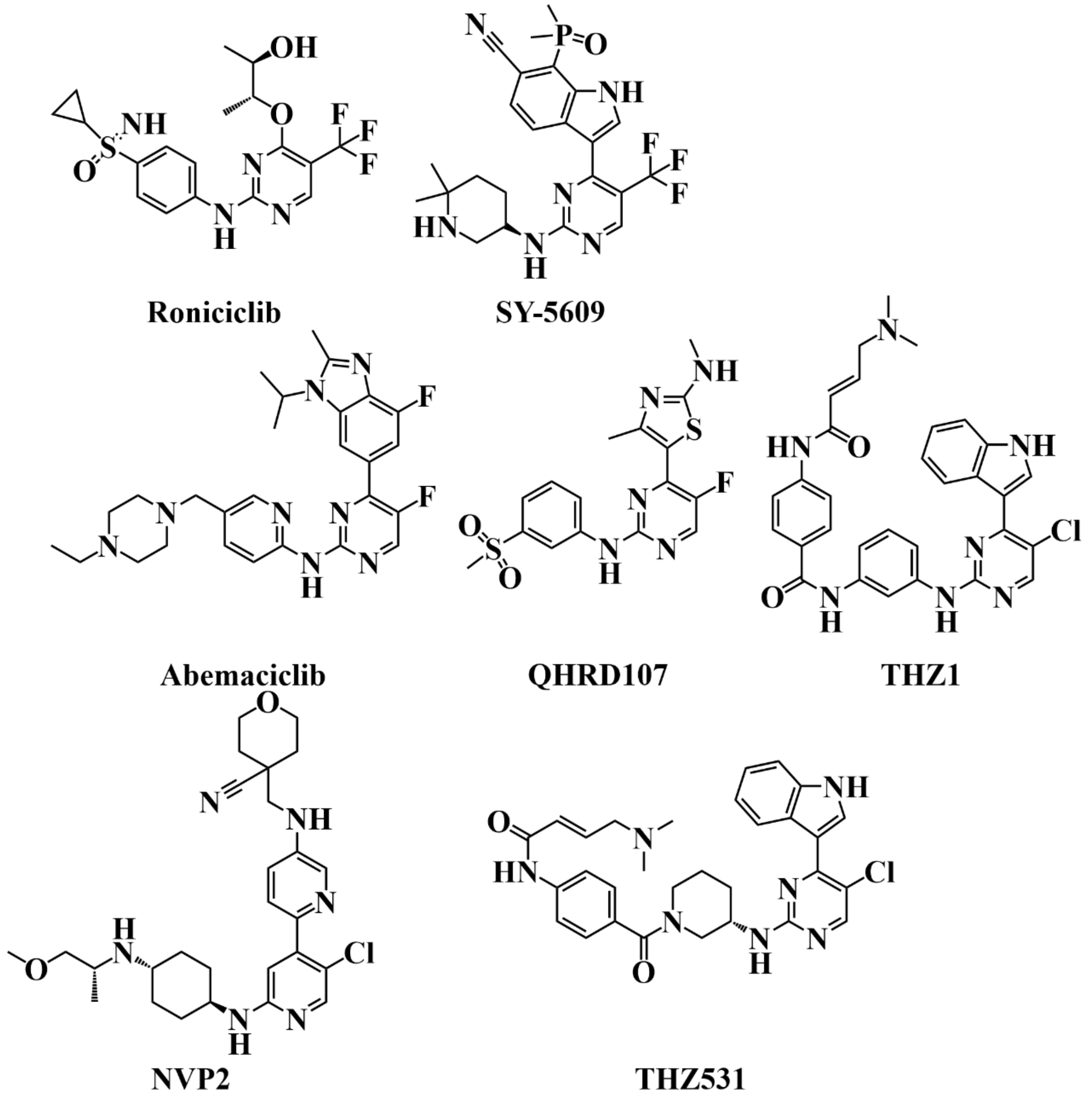

Figure S28. Structure for CDK inhibitors with different R2 groups.

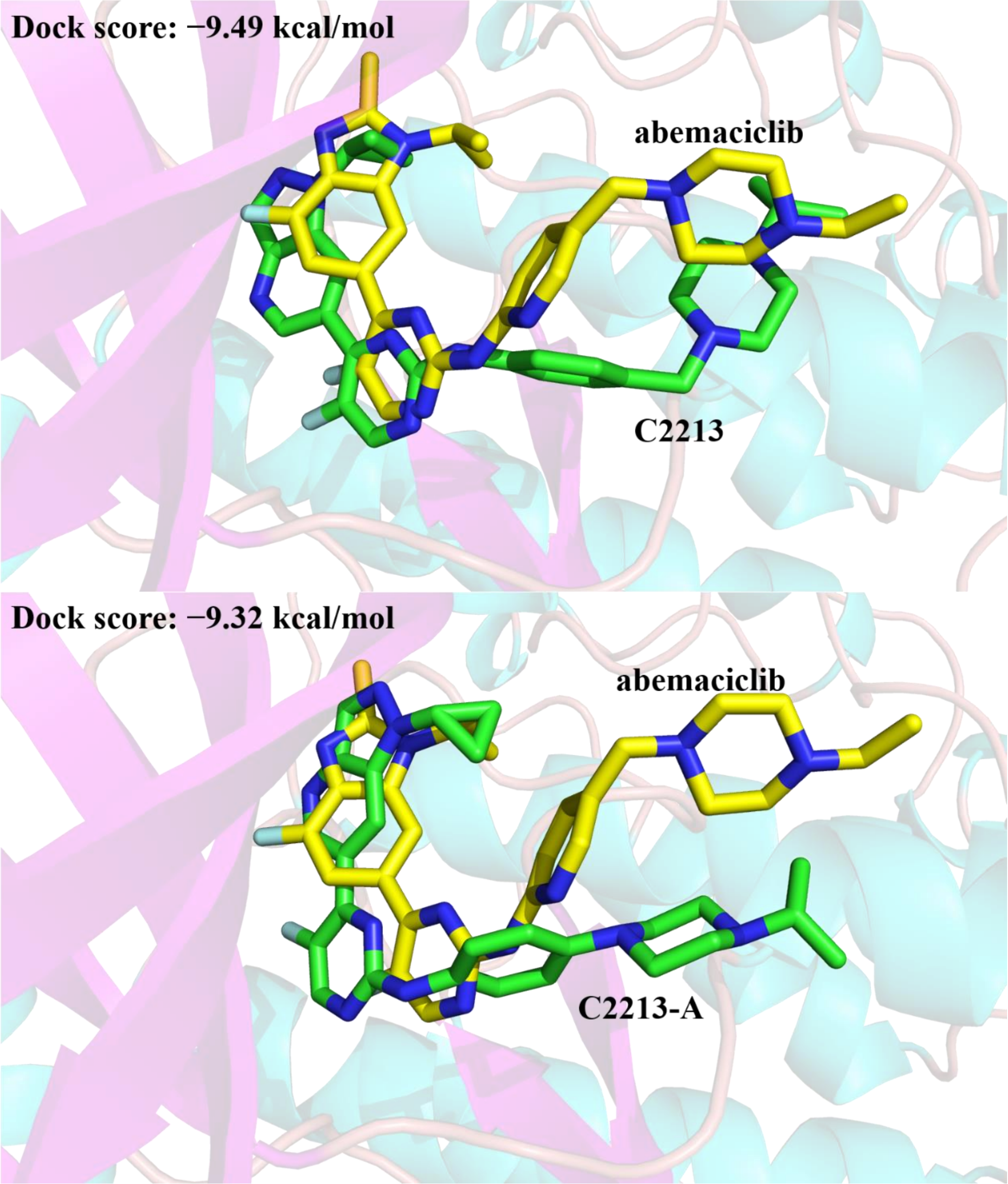

Figure S29. Lowest binding energy conformation of C2213 and C2213-A from molecular docking.

Abemaciclib/CDK6 complex structure was also shown in this figure to compare. Human CDK6 shown with cartoon and ligand molecules with stick (abemaciclib for yellow, C2213 and C2213-A for green).

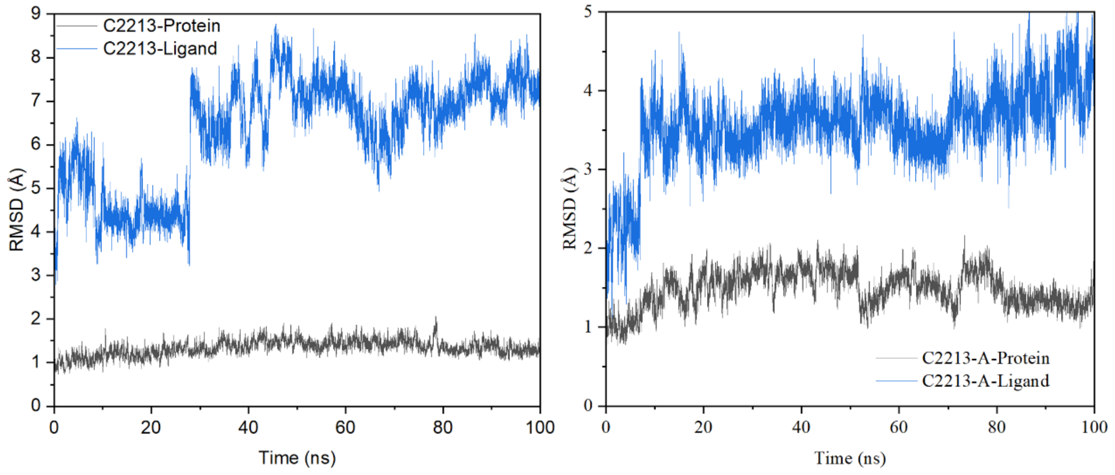

Figure S30. Root mean square deviation (RMSD) value of heavy atoms of backbone for human CDK6 and along 500 ns MD simulation for **C2213**/CDK6 and **C2213-A**/CDK6 systems.

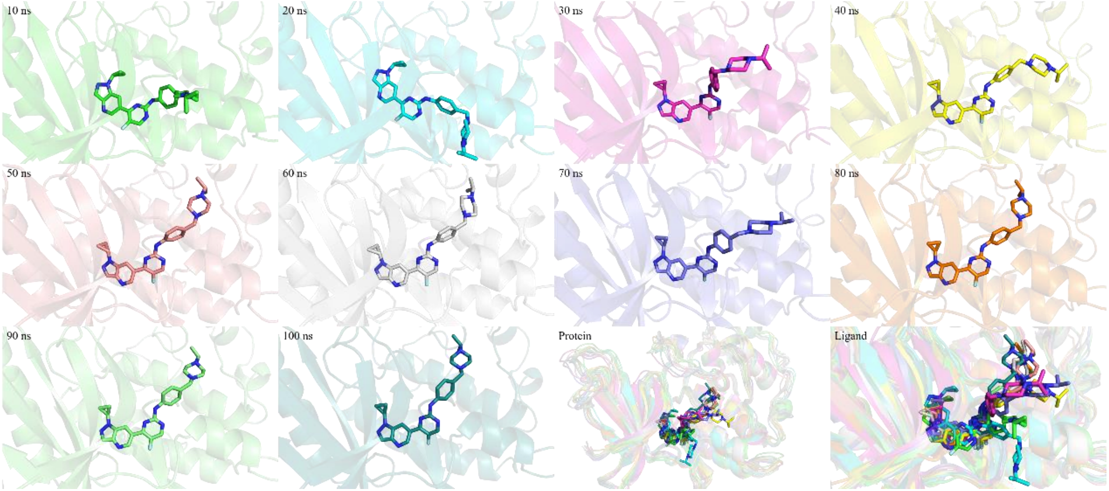

Figure S31. Snapshots of **C2213**/CDK6 along the dynamic simulation time for 10, 20, 30, 40, 50, 60, 70, 80, 90, and 100 ns.

For clarity, the water molecules have been removed. The inhibitor **C2213** is plotted using stick style, while cartoon style for human CDK6.

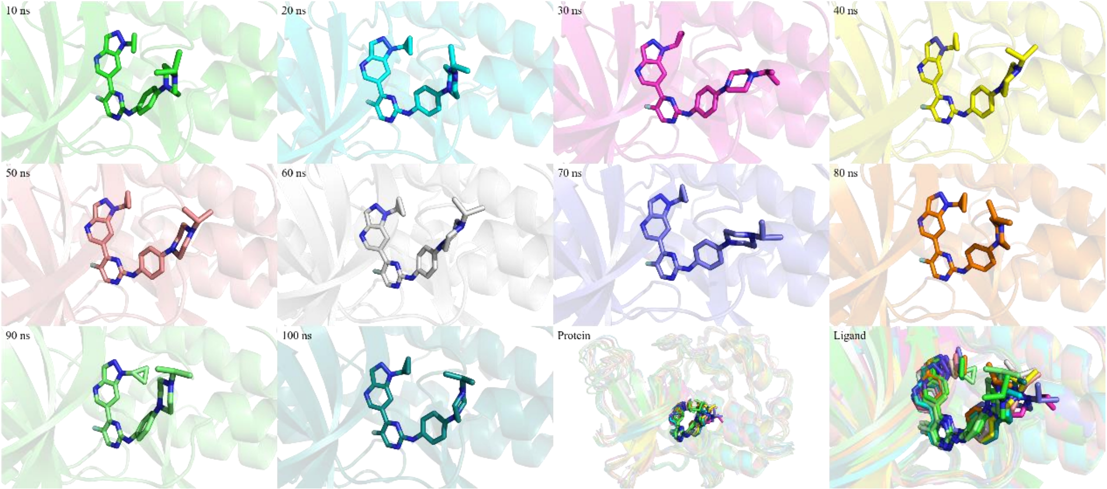

Figure S32. Snapshots of **C2213-A**/CDK6 along the dynamic simulation time for 10, 20, 30, 40, 50, 60, 70, 80, 90, and 100 ns.

For clarity, the water molecules have been removed. The inhibitor **C2213-A** is plotted using stick style, while cartoon style for human CDK6.

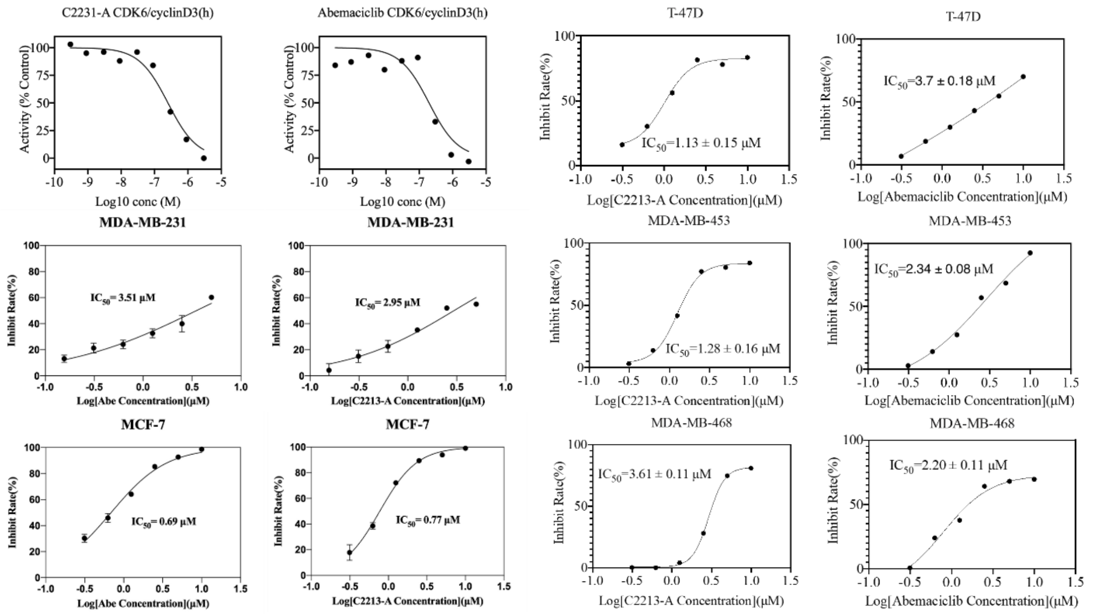

Figure S33. Biochemical and cellular profiling for abemaciclib and C2213-A for MDA-MB-231, MCF-7, T-47D, MDA-MB-453, and MDA-MB-468.

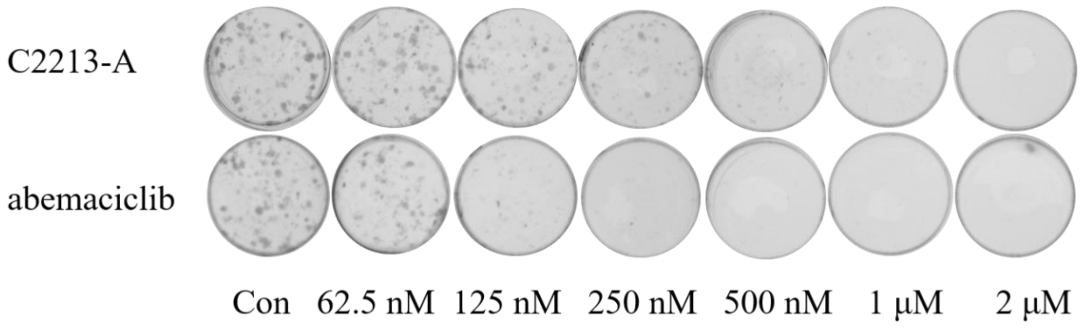

Figure S34. Colony-formation assays for MDA-MB-231 with different concentration C2231-A and abemaciclib.

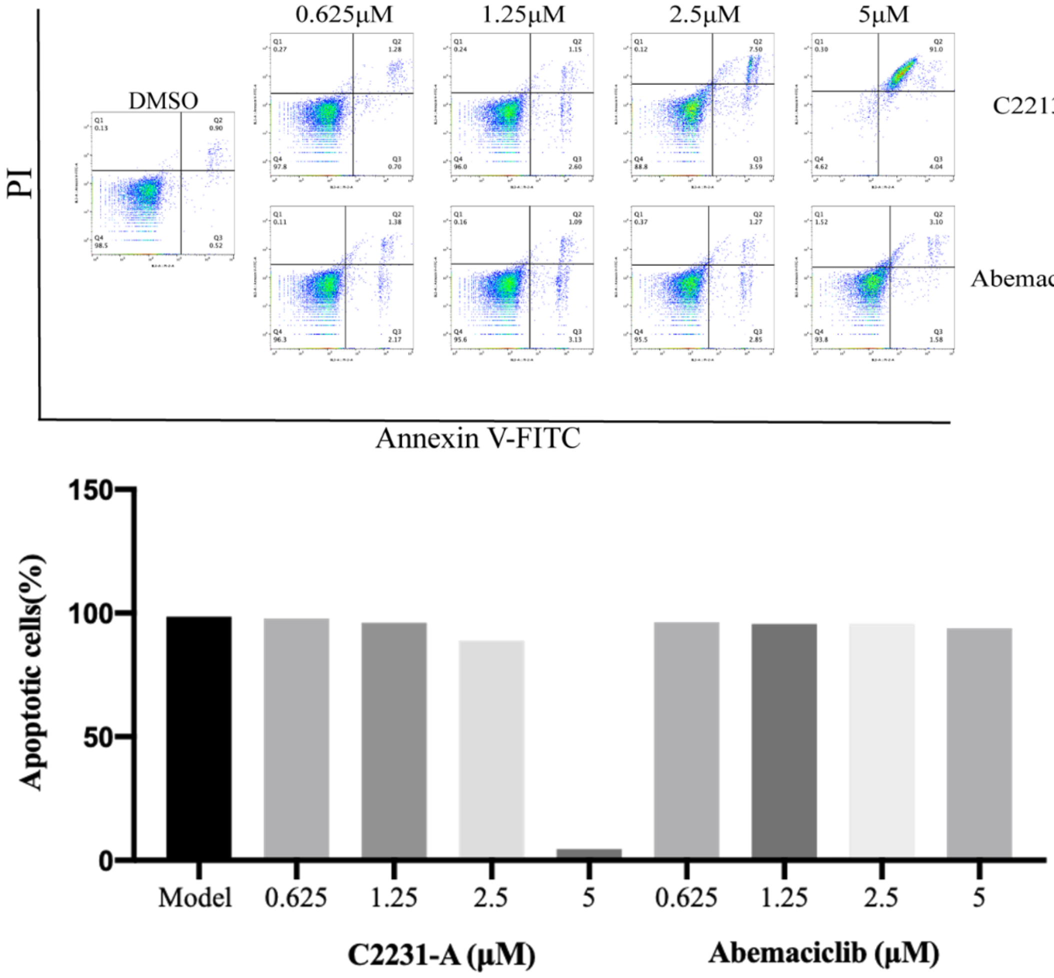

Figure S35. Percentage of apoptotic cells treatment with different concentration of compound C2213-A for 48 h.

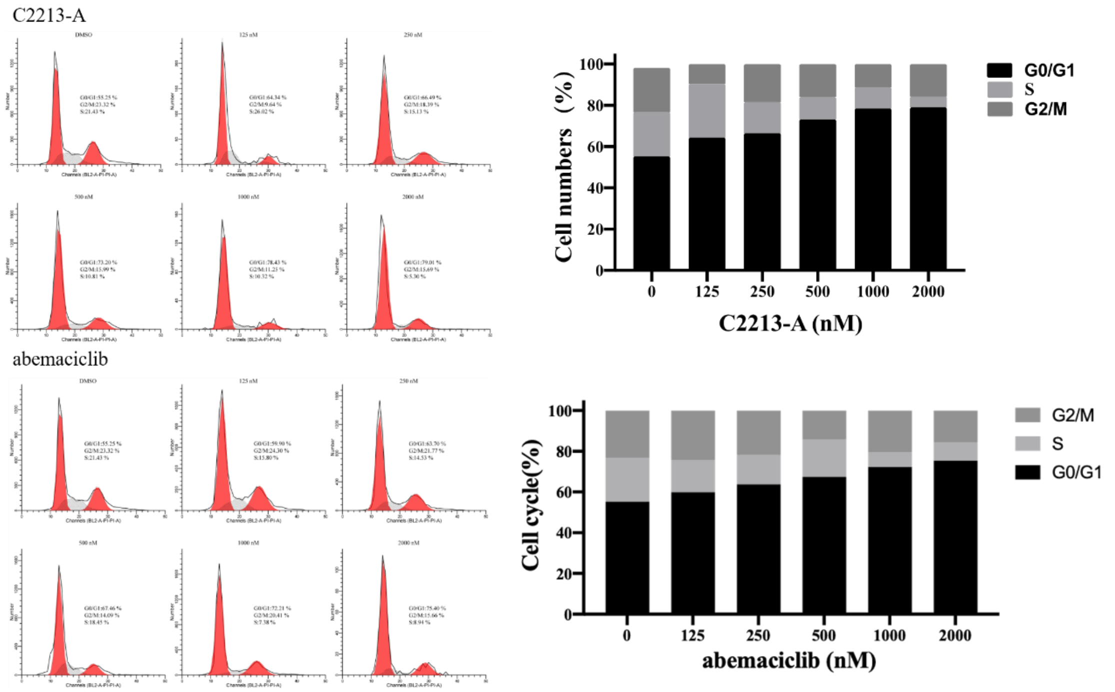

Figure S36. MDA-MB-231 cell lines exhibited an obvious G1 arrest and a decrease of S phase after incubation with different doses of compound C2213-A or abemaciclib.

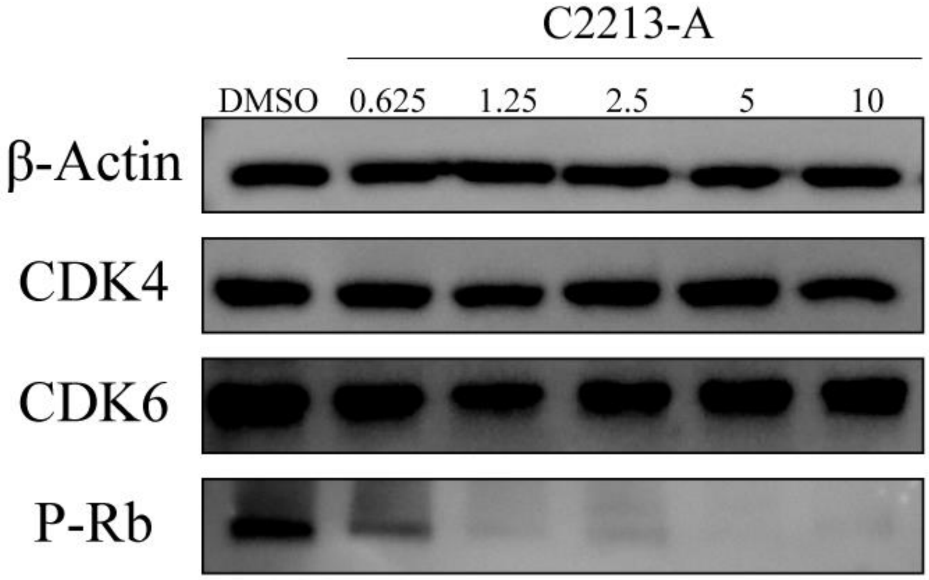

Figure S37. Compound C2213-A inhibited the phosphorylation of Rb at S780 in a dose-dependent manner in MDA-MB-231.

Table S1. Binding free energies ($\Delta\mathbf{G}_{\mathbf{bind}}^{\mathbf{cal}}$) for abemaciclib/CDK6 system.

Energy	Complex Average	Complex Std. Dev.	Receptor Average	Receptor Std. Dev.	Ligand Average	Ligand Std. Dev.	Delta Average	Delta Std. Dev.
$\mathrm{E}_{\mathrm{vdW}}$	−2396.80	22.90	−2339.28	22.57	−5.83	1.64	−51.70	3.79
$\mathrm{E}_{\mathrm{ele}}$	−19936.00	107.15	−20063.71	106.60	155.02	2.21	−27.31	4.10
E_{GB}	−3259.72	85.71	−3291.36	85.26	−19.65	0.64	51.29	4.10
E_{surf}	104.35	2.19	106.18	2.10	4.37	0.09	−6.20	0.36
E_{gas}	−6224.90	109.14	−5990.32	108.36	−155.57	5.95	−79.01	5.27
E_{solv}	−3155.37	84.50	−3185.18	84.14	−15.28	0.63	45.09	3.98
$\mathrm{E}_{\mathrm{gas}} + E_{\mathrm{sol}}$	−9380.27	51.69	−9175.50	51.36	−170.84	5.90	−33.92	3.70
$\mathrm{TS}_{\mathrm{total}}$	3282.60	9.02	3241.42	7.85	65.36	0.53	−24.18	4.82
$\Delta\mathrm{G}_{\mathrm{bind}}^{\mathrm{cal}}$							−9.74	6.07
$\Delta\mathrm{G}_{\mathrm{bind}}^{exp}$							−9.60	

The binding free energies ($\Delta\mathrm{G}_{\mathrm{bind}}^{\mathrm{cal}}$) for abemaciclib/CDK6 complex and decomposition to electrostatic interaction ($\mathrm{E}_{\mathrm{ele}}$), van der Walls interaction ($\mathrm{E}_{\mathrm{vdW}}$), polar solvation free energies (E_{GB}), nonpolar solvation free energies ($\mathrm{E}_{\mathrm{surf}}$), and entropy ($\mathrm{TS}_{\mathrm{total}}$). Energy values are presented in kcal/mol. Uncertainties were calculated as the root mean square error for all frames extracted from the trajectories.

Table S2. Binding free energies ($\Delta \mathbf{G}_{\mathbf{bind}}^{\mathbf{cal}}$) for abemaciclib/CDK6 system (replicate 2).

Energy	Complex		Receptor		Ligand		Delta	
	Average	Std. Dev.	Average	Std. Dev.	Average	Std. Dev.	Average	Std. Dev.
E_{vdW}	−2395.78	23.68	−2338.10	23.16	−5.87	1.59	−51.82	3.65
E_{ele}	−19935.04	104.24	−20063.06	103.80	154.98	2.13	−26.95	4.06
E_{GB}	−3259.78	84.98	−3291.25	84.59	−19.64	0.64	51.12	4.13
E_{surf}	104.32	2.23	106.15	2.12	4.38	0.09	−6.20	0.37
E_{gas}	−6226.58	107.51	−5992.27	106.47	−155.53	5.88	−78.77	5.38
E_{solv}	−3155.46	83.79	−3185.11	83.49	−15.27	0.63	44.91	3.99
$E_{gas} + E_{sol}$	−9382.04	53.32	−9177.38	52.80	−170.80	5.84	−33.86	3.67
TS_{total}	3284.54	7.09	3244.06	6.71	65.36	0.54	−24.87	6.34
ΔG_{bind}^{cal}							−8.99	7.32
ΔG_{bind}^{exp}							−9.60	

The binding free energies (ΔG_{bind}^{cal}) for abemaciclib/CDK6 complex and decomposition to electrostatic interaction (E_{ele}), van der Walls interaction (E_{vdW}), polar solvation free energies (E_{GB}), nonpolar solvation free energies (E_{surf}), and entropy (TS_{total}). Energy values are presented in kcal/mol. Uncertainties were calculated as the root mean square error for all frames extracted from the trajectories.

Table S3. Binding free energies ($\Delta \mathbf{G}_{\mathbf{bind}}^{\mathbf{cal}}$) for abemaciclib/CDK6 system (replicate 3).

Energy	Complex Average	Std. Dev.	Receptor Average	Std. Dev.	Ligand Average	Std. Dev.	Delta Average	Std. Dev.
E_{vdW}	−2396.84	23.86	−2339.26	23.38	−5.93	1.60	−51.65	3.75
E_{ele}	−19930.59	107.14	−20058.25	106.87	155.03	2.10	−27.37	3.89
E_{GB}	−3263.30	87.02	−3295.00	86.65	−19.67	0.64	51.36	4.07
E_{surf}	104.37	2.21	106.19	2.13	4.38	0.09	−6.19	0.37
E_{gas}	−6221.63	109.54	−5986.88	108.81	−155.72	5.84	−79.02	5.20
E_{solv}	−3158.93	85.78	−3188.81	85.48	−15.29	0.63	45.17	3.92
$E_{gas} + E_{sol}$	−9380.56	53.02	−9175.69	52.69	−171.01	5.79	−33.85	3.60
TS_{total}	3282.26	6.43	3238.38	6.16	65.48	0.54	−21.59	4.82
ΔG_{bind}^{cal}							−12.26	6.02
ΔG_{bind}^{exp}							−9.60	

The binding free energies (ΔG_{bind}^{cal}) for abemaciclib/CDK6 complex and decomposition to electrostatic interaction (E_{ele}), van der Walls interaction (E_{vdW}), polar solvation free energies (E_{GB}), nonpolar solvation free energies (E_{surf}), and entropy (TS_{total}). Energy values are presented in kcal/mol. Uncertainties were calculated as the root mean square error for all frames extracted from the trajectories.

Table S4. Binding free energies ($\mathbf{\Delta G_{bind}^{cal}}$) for abemaciclib/CDK6 system as cluster 1.

Energy	Complex		Receptor		Ligand		Delta	
	Average	Std. Dev.	Average	Std. Dev.	Average	Std. Dev.	Average	Std. Dev.
E_{vdW}	−2392.65	24.10	−2335.92	23.45	−6.19	1.57	−50.54	3.62
E_{ele}	−19949.54	94.27	−20077.02	94.34	155.28	2.12	−27.80	3.84
E_{GB}	−3257.70	73.41	−3289.43	73.54	−19.71	0.63	51.45	3.94
E_{surf}	104.74	2.00	106.49	1.92	4.37	0.08	−6.12	0.35
E_{gas}	−6228.65	90.66	−5995.10	90.41	−155.22	5.42	−78.34	5.09
E_{solv}	−3152.96	72.42	−3182.95	72.58	−15.33	0.62	45.32	3.82
$E_{gas} + E_{sol}$	−9381.61	50.97	−9178.04	50.64	−170.55	5.41	−33.02	3.59
TS_{total}	3284.09	7.71	3245.57	7.90	65.48	0.56	−26.96	7.42
ΔG_{bind}^{cal}							−6.05	8.24
ΔG_{bind}^{exp}							−9.60	

The binding free energies (ΔG_{bind}^{cal}) for abemaciclib/CDK6 complex and decomposition to electrostatic interaction (E_{ele}), van der Walls interaction (E_{vdW}), polar solvation free energies (E_{GB}), nonpolar solvation free energies (E_{surf}), and entropy (TS_{total}). Energy values are presented in kcal/mol. Uncertainties were calculated as the root mean square error for all frames extracted from the trajectories.

Table S5. Binding free energies ($\Delta \mathbf{G}_{\mathbf{bind}}^{\mathbf{cal}}$) for abemaciclib/CDK6 system as cluster 2.

Energy	Complex		Receptor		Ligand		Delta	
	Average	Std. Dev.	Average	Std. Dev.	Average	Std. Dev.	Average	Std. Dev.
E_{vdW}	−2389.68	24.39	−2332.30	24.36	−5.51	1.61	−51.87	3.66
E_{ele}	−19884.87	111.23	−20014.06	110.96	154.72	2.13	−25.52	3.94
E_{GB}	−3306.57	89.64	−3337.00	89.60	−19.55	0.65	49.98	3.69
E_{surf}	105.79	2.20	107.51	2.19	4.39	0.07	−6.11	0.34
E_{gas}	−6166.86	110.80	−5933.80	110.12	−155.66	5.79	−77.39	5.15
E_{solv}	−3200.77	88.80	−3229.49	88.81	−15.16	0.63	43.88	3.51
$E_{gas} + E_{sol}$	−9367.63	54.28	−9163.29	53.56	−170.82	5.74	−33.52	3.54
TS_{total}	3284.01	7.16	3244.61	8.13	65.46	0.50	−26.07	4.34
ΔG_{bind}^{cal}							−7.45	5.60
ΔG_{bind}^{exp}							−9.60	

The binding free energies (ΔG_{bind}^{cal}) for abemaciclib/CDK6 complex and decomposition to electrostatic interaction (E_{ele}), van der Walls interaction (E_{vdW}), polar solvation free energies (E_{GB}), nonpolar solvation free energies (E_{surf}), and entropy (TS_{total}). Energy values are presented in kcal/mol. Uncertainties were calculated as the root mean square error for all frames extracted from the trajectories.

Table S6. Free energy decomposition for abemaciclib/CDK6 complex on the individual residue basis.

Residues	ΔE_{vdW}		ΔE_{ele}		ΔE_{GB}		ΔE_{surf}		$\Delta E_{subtotal}$		$S\Delta E_{subtotal}$		$B\Delta E_{subtotal}$	
	Avg.	Std. Dev.	Avg.	Std. Dev.	Avg.	Std. Dev.	Avg.	Std. Dev.	Avg.	Std. Dev.	Avg.	Std. Dev.	Avg.	Std. Dev.
I19	−3.67	0.62	−0.78	0.21	1.77	0.59	−0.60	0.07	−3.27	0.72	−3.59	0.62	0.31	0.44
V27	−1.70	0.30	−0.32	0.09	0.22	0.08	−0.15	0.03	−1.95	0.30	−1.78	0.30	−0.17	0.07
K29	−0.84	0.33	−1.71	1.10	2.10	1.68	−0.08	0.05	−0.53	0.75	−0.19	0.70	−0.34	0.10
A41	−1.35	0.17	−0.07	0.08	0.14	0.10	−0.13	0.02	−1.40	0.18	−1.36	0.16	−0.05	0.08
K43	−0.81	0.46	−7.62	1.25	5.36	1.23	−0.07	0.02	−3.14	1.03	−2.77	1.03	−0.37	0.10
V77	−0.54	0.15	0.02	0.04	−0.44	0.06	−0.03	0.01	−0.99	0.16	−0.70	0.15	−0.28	0.04
F98	−1.15	0.20	0.03	0.07	0.12	0.09	−0.08	0.02	−1.08	0.21	−0.87	0.18	−0.21	0.07
E99	−0.31	0.39	−0.23	0.28	2.29	0.33	−0.02	0.01	1.72	0.44	0.08	0.09	1.64	0.44
H100	−1.49	0.39	−1.65	0.68	2.08	0.54	−0.11	0.04	−1.17	0.51	0.05	0.41	−1.23	0.29
V101	−1.07	0.36	−0.69	0.62	0.90	0.49	−0.05	0.02	−0.91	0.43	−0.76	0.18	−0.15	0.44
D104	−0.67	0.48	−1.46	0.45	3.90	2.03	−0.13	0.06	1.64	1.39	1.50	1.42	0.14	0.12
N150	−0.74	0.15	0.45	0.12	−0.31	0.19	−0.05	0.02	−0.65	0.28	−0.52	0.27	−0.13	0.07
L152	−1.66	0.38	−0.07	0.06	−0.38	0.13	−0.25	0.04	−2.36	0.43	−2.24	0.41	−0.12	0.05
A162	−0.85	0.20	−0.44	0.07	0.28	0.08	−0.08	0.02	−1.09	0.22	−0.82	0.18	−0.27	0.07
D163	−2.02	0.35	−0.09	0.93	6.25	1.57	−0.20	0.03	3.94	1.30	3.89	1.26	0.05	0.11
R168	−0.34	0.09	0.17	0.46	−1.15	0.50	−0.01	0.01	−1.32	0.41	−1.31	0.41	−0.01	0.01

The total energy ($\Delta E_{subtotal}$) decomposition is performed in terms of the contributions from van der Waals energy (ΔE_{vdW}), the electrostatic interaction energy (ΔE_{ele}), the polar solvation free energy (ΔE_{GB}), the nonpolar solvation free energy (ΔE_{surf}), the backbone energy ($B\Delta E_{subtotal}$), and the side chain energy ($S\Delta E_{subtotal}$). Energy values are presented in kcal/mol.

Table S7. Docking results for the designing compounds from one region.

Name	SMILES	Docking Energy (kcal/mol)	
		Cluster1	Cluster2
abemaciclib	FC(C(C1=CC2=C(C(F)=C1)N=C(N2C(C)C)C)=N3)=CN=C3NC(C=C4)=NC=C4CN5CCN(CC)CC5	−9.46	−9.19
R1a	CCN1CCN(CC1)CC2=CN=C(C=C2)NC3=NC=C(C(C4=CC5=C(N=C4)N=C(N5C(C)C)C)=N3)F	−9.31	−8.83
R1b	CCN1CCN(CC1)CC2=CN=C(C=C2)NC3=NC=C(C(C4=CC5=C(C(F)=C4)N=NN5C(C)C)=N3)F	−9.32	−9.75
R1c	CCN1CCN(CC1)CC2=CN=C(C=C2)NC3=NC=C(C(C4=CC5=C(N=C4)N=NN5C(C)C)=N3)F	−9.19	−9.04
R1d	CCN1CCN(CC1)CC2=CN=C(C=C2)NC3=NC=C(C(C4=CC5=C(N=C4)N=NN5CC)=N3)F	−8.78	−8.86
R1e	CCN1CCN(CC1)CC2=CN=C(C=C2)NC3=NC=C(C(C4=CC5=C(N=C4)N=NN5C6CC6)=N3)F	−9.08	−9.15
R1f	CCN1CCN(CC1)CC2=CN=C(C=C2)NC3=NC=C(C(C4=CC5=C(N=C4)N=NN5C6CCCC6)=N3)F	−10.03	−9.86
R1g	CCN1CCN(CC1)CC2=CN=C(C=C2)NC3=NC=C(C(C4=CC5=C(N=C4)N=NN5C)=N3)F	−9.00	−9.42
R1h	CCN1CCN(CC1)CC2=CN=C(C=C2)NC3=NC=C(C(C4=CC5=C(N=C4)C=NN5C(C)C)=N3)F	−8.53	−9.06
R1i	CCN1CCN(CC1)CC2=CN=C(C=C2)NC3=NC=C(C(C4=CC5=C(C(F)=C4)C=NN5C(C)C)=N3)F	−9.30	−9.21
R1j	CCN1CCN(CC1)CC2=CN=C(C=C2)NC3=NC=C(C(C4=CC5=C(N=C4)C=NN5CC)=N3)F	−8.58	−8.80
R1k	CCN1CCN(CC1)CC2=CN=C(C=C2)NC3=NC=C(C(C4=CC5=C(N=C4)C=NN5C)=N3)F	−8.63	−8.64
R1l	CCN1CCN(CC1)CC2=CN=C(C=C2)NC3=NC=C(C(C4=CC5=C(N=C4)C=NN5C6CC6)=N3)F	−8.61	−8.86
R1m	CCN1CCN(CC1)CC2=CN=C(C=C2)NC3=NC=C(C(C4=CC5=C(N=C4)C=NN5C6CCCC6)=N3)F	−9.78	−9.22
R2a	CCN1CCN(CC1)CC2=CN=C(C=C2)NC3=NC=C(C(C4=CC5=C(C(F)=C4)N=C(N5C(C)C)C)=N3)C	−9.71	−9.54
R2b	CCN1CCN(CC1)CC2=CN=C(C=C2)NC3=NC=C(C(C4=CC5=C(C(F)=C4)N=C(N5C(C)C)C)=N3)C6CC6	−9.72	−9.44
R2c	CCN1CCN(CC1)CC2=CN=C(C=C2)NC3=NC=C(C(C4=CC5=C(C(F)=C4)N=C(N5C(C)C)C)=N3)C(NC6CC6)=O	−9.40	−9.36
R2d	CCN1CCN(CC1)CC2=CN=C(C=C2)NC3=NC=C(C(C4=CC5=C(C(F)=C4)N=C(N5C(C)C)C)=N3)NC(C6CC6)=O	−9.25	−8.78
R2e	CCN1CCN(CC1)CC2=CN=C(C=C2)NC3=NC=C(C(C4=CC5=C(C(F)=C4)N=C(N5C(C)C)C)=N3)C(C)=O	−9.74	−9.31
R2f	CCN1CCN(CC1)CC2=CN=C(C=C2)NC3=NC=C(C(C4=CC5=C(C(F)=C4)N=C(N5C(C)C)C)=N3)C(C6CC6)=O	−9.48	−9.49
R2g	CCN1CCN(CC1)CC2=CN=C(C=C2)NC3=NC=C(C(C4=CC5=C(C(F)=C4)N=C(N5C(C)C)C)=N3)C(C)C	−9.42	−9.65
R2h	CCN1CCN(CC1)CC2=CN=C(C=C2)NC3=NC=C(C(C4=CC5=C(C(F)=C4)N=C(N5C(C)C)C)=N3)CC	−9.86	−9.26
R2i	CCN1CCN(CC1)CC2=CN=C(C=C2)NC3=NC=CC(C4=CC5=C(C(F)=C4)N=C(N5C(C)C)C)=N3	−9.70	−10.01
R2j	CCN1CCN(CC1)CC2=CN=C(C=C2)NC3=NC=C(OC)C(C4=CC5=C(C(F)=C4)N=C(N5C(C)C)C)=N3	−9.30	−8.98

R3a	FC(C(C1=CC2=C(C(F)=C1)N=C(N2C(C)C)C)=N3)=CN=C3NC(C=C4)=CC=C4CN5CCN(CC)CC5	−9.85	−9.87
R3b	FC(C(C1=CC2=C(C(F)=C1)N=C(N2C(C)C)C)=N3)=CN=C3NC(C=C4)=CC(F)=C4CN5CCN(CC)CC5	−9.63	−9.79
R3c	FC(C(C1=CC2=C(C(F)=C1)N=C(N2C(C)C)C)=N3)=CN=C3NC(C=C4)=CC(C)=C4CN5CCN(CC)CC5	−8.83	−9.09
R3d	FC(C(C1=CC2=C(C(F)=C1)N=C(N2C(C)C)C)=N3)=CN=C3NC(C=C4)=CC(O)=C4CN5CCN(CC)CC5	−8.91	−8.83
R3e	FC(C(C1=CC2=C(C(F)=C1)N=C(N2C(C)C)C)=N3)=CN=C3NC(C=C4)=C(C)C=C4CN5CCN(CC)CC5	−10.39	−9.93
R3f	FC(C(C1=CC2=C(C(F)=C1)N=C(N2C(C)C)C)=N3)=CN=C3NC(C=C4)=C(C)C=C4CN5CCN(CC)CC5	−10.37	−9.94
R3g	FC(C(C1=CC2=C(C(F)=C1)N=C(N2C(C)C)C)=N3)=CN=C3NC(C=C4)=C(O)C=C4CN5CCN(CC)CC5	−9.15	−9.22
R3h	FC(C(C1=CC2=C(C(F)=C1)N=C(N2C(C)C)C)=N3)=CN=C3NC(C=C4)=CC(OC)=C4CN5CCN(CC)CC5	−9.58	−9.54
R3i	FC(C(C1=CC2=C(C(F)=C1)N=C(N2C(C)C)C)=N3)=CN=C3NC(C=C4)=C(OC)C=C4CN5CCN(CC)CC5	−10.28	−9.81
R4a	FC(C(C1=CC2=C(C(F)=C1)N=C(N2C(C)C)C)=N3)=CN=C3NC(C=C4)=NC=C4CN5CCNCC5	−10.31	−10.12
R4b	FC(C(C1=CC2=C(C(F)=C1)N=C(N2C(C)C)C)=N3)=CN=C3NC(C=C4)=NC=C4CN5CCN(C(C)C)CC5	−9.87	−9.42
R4c	FC(C(C1=CC2=C(C(F)=C1)N=C(N2C(C)C)C)=N3)=CN=C3NC(C=C4)=NC=C4CN5CCN(C(CC6CC6)=O)CC5	−9.42	−9.56
R4d	FC(C(C1=CC2=C(C(F)=C1)N=C(N2C(C)C)C)=N3)=CN=C3NC(C=C4)=NC=C4CN5CCN(C(C6CC6)=O)CC5	−9.81	−9.43
R4e	FC(C(C1=CC2=C(C(F)=C1)N=C(N2C(C)C)C)=N3)=CN=C3NC(C=C4)=NC=C4CN5CCN(C(C)=O)CC5	−9.68	−9.38
R4f	FC(C(C1=CC2=C(C(F)=C1)N=C(N2C(C)C)C)=N3)=CN=C3NC(C=C4)=NC=C4CN5CCN(C(CC)=O)CC5	−9.34	−9.10
R4g	FC(C(C1=CC2=C(C(F)=C1)N=C(N2C(C)C)C)=N3)=CN=C3NC(C=C4)=NC=C4CN5CCN(C)CC5	−10.13	−10.01
R4h	FC(C(C1=CC2=C(C(F)=C1)N=C(N2C(C)C)C)=N3)=CN=C3NC(C=C4)=NC=C4CN5CCN(C([C@H]6C[C@H]6F)=O)CC5	−9.00	−9.12
R4i	FC(C(C1=CC2=C(C(F)=C1)N=C(N2C(C)C)C)=N3)=CN=C3NC(C=C4)=NC=C4CN5CCN(C6CC6)CC5	−9.60	−9.35
R4j	FC(C(C1=CC2=C(C(F)=C1)N=C(N2C(C)C)C)=N3)=CN=C3NC(C=C4)=NC=C4CN5CCN(C6CCCC6)CC5	−10.01	−9.74

The centroid frame for every cluster was selected as the representative complex structures and labeled as Cluster1 and Cluster2. The docking score was obtained from the molecular docking by Autodock4.2 softwater.

Table S8. Docking results for the designing compounds with four region combination.

Compounds	SMILES	Cluster1	Cluster2	Dock Score
abemaciclib	FC(C(C1=CC2=C(C(F)=C1)N=C(N2C(C)C)C)=N3)=CN=C3NC(C=C4)=NC=C4CN5CCN(CC)CC5	−9.46	−9.19	−9.46
C1111	CC(C)N1N=CC(C1=C2)=NC=C2C3=CC=NC(NC(C=C4)=CC=C4CN5CCNCC5)=N3	−9.96	−10.26	−10.26
C1112	CN1CCN(CC1)CC2=CC=C(C=C2)NC3=NC(C4=CN=C(C=NN5C(C)C)C5=C4)=CC=N3	−9.39	−9.42	−9.42
C1113	CC(C)N1CCN(CC1)CC2=CC=C(C=C2)NC3=NC(C4=CN=C(C=NN5C(C)C)C5=C4)=CC=N3	−9.29	−9.34	−9.34
C1121	CC(C)N1N=CC(C1=C2)=NC=C2C3=CC=NC(NC(C=C4)=CC(F)=C4CN5CCNCC5)=N3	−9.95	−9.79	−9.95
C1122	CC(C)N1N=CC(C1=C2)=NC=C2C3=CC=NC(NC(C=C4)=CC(F)=C4CN5CCN(C)CC5)=N3	−9.40	−9.41	−9.41
C1123	CC(C)N1CCN(CC1)CC2=C(F)C=C(C=C2)NC3=NC(C4=CN=C(C=NN5C(C)C)C5=C4)=CC=N3	−8.86	−9.61	−9.61
C1131	CC(C)N1N=CC(C1=C2)=NC=C2C3=CC=NC(NC(C=C4)=CC(OC)=C4CN5CCNCC5)=N3	−9.99	−9.77	−9.99
C1132	CN1CCN(CC1)CC2=C(OC)C=C(C=C2)NC3=NC(C4=CN=C(C=NN5C(C)C)C5=C4)=CC=N3	−9.21	−9.25	−9.25
C1133	CC(C)N1CCN(CC1)CC2=C(OC)C=C(C=C2)NC3=NC(C4=CN=C(C=NN5C(C)C)C5=C4)=CC=N3	−8.99	−9.44	−9.44
C1211	FC(C=N1)=C(C2=CN=C(C=NN3C(C)C)C3=C2)N=C1NC(C=C4)=CC=C4CN5CCNCC5	−9.50	−9.81	−9.81
C1212	CN1CCN(CC1)CC2=CC=C(C=C2)NC3=NC(C4=CN=C(C=NN5C(C)C)C5=C4)=C(C=N3)F	−9.18	−9.45	−9.45
C1213	CC(C)N1CCN(CC1)CC2=CC=C(C=C2)NC3=NC(C4=CN=C(C=NN5C(C)C)C5=C4)=C(C=N3)F	−9.10	−9.28	−9.28
C1221	FC(C=N1)=C(C2=CN=C(C=NN3C(C)C)C3=C2)N=C1NC(C=C4)=CC(F)=C4CN5CCNCC5	−9.39	−9.96	−9.96
C1222	CN1CCN(CC1)CC2=C(F)C=C(C=C2)NC3=NC(C4=CN=C(C=NN5C(C)C)C5=C4)=C(C=N3)F	−9.18	−9.14	−9.18
C1223	CC(C)N1CCN(CC1)CC2=C(F)C=C(C=C2)NC3=NC(C4=CN=C(C=NN5C(C)C)C5=C4)=C(C=N3)F	−8.96	−9.72	−9.72
C1231	FC(C=N1)=C(C2=CN=C(C=NN3C(C)C)C3=C2)N=C1NC(C=C4)=CC(OC)=C4CN5CCNCC5	−10.09	−10.10	−10.10
C1232	CN1CCN(CC1)CC2=C(OC)C=C(C=C2)NC3=NC(C4=CN=C(C=NN5C(C)C)C5=C4)=C(C=N3)F	−9.04	−9.13	−9.13
C1233	CC(C)N1CCN(CC1)CC2=C(OC)C=C(C=C2)NC3=NC(C4=CN=C(C=NN5C(C)C)C5=C4)=C(C=N3)F	−8.90	−9.09	−9.09
C1311	CC(C)N1N=CC(C1=C2)=NC=C2C3=C(C)C=NC(NC(C=C4)=CC=C4CN5CCNCC5)=N3	−10.76	−10.25	−10.76
C1312	CN1CCN(CC1)CC2=CC=C(C=C2)NC3=NC(C4=CN=C(C=NN5C(C)C)C5=C4)=C(C)C=N3	−9.74	−9.79	−9.79

C1313	CC(C)N1CCN(CC1)CC2=CC=C(C=C2)NC3=NC(C4=CN=C(C=NN5C(C)C)C5=C4)=C(C)C=N3	−9.56	−9.85	−9.85
C1321	CC(C)N1N=CC(C1=C2)=NC=C2C3=C(C)C=NC(NC(C=C4)=CC(F)=C4CN5CCNCC5)=N3	−10.00	−10.35	−10.35
C1322	CN1CCN(CC1)CC2=C(F)C=C(C=C2)NC3=NC(C4=CN=C(C=NN5C(C)C)C5=C4)=C(C)C=N3	−9.78	−9.74	−9.78
C1323	CC(C)N1CCN(CC1)CC2=C(F)C=C(C=C2)NC3=NC(C4=CN=C(C=NN5C(C)C)C5=C4)=C(C)C=N3	−9.45	−9.53	−9.53
C1331	CC(C)N1N=CC(C1=C2)=NC=C2C3=C(C)C=NC(NC(C=C4)=CC(OC)=C4CN5CCNCC5)=N3	−10.39	−10.44	−10.44
C1332	CN1CCN(CC1)CC2=C(OC)C=C(C=C2)NC3=NC(C4=CN=C(C=NN5C(C)C)C5=C4)=C(C)C=N3	−9.75	−9.85	−9.85
C1333	CC(C)N1CCN(CC1)CC2=C(OC)C=C(C=C2)NC3=NC(C4=CN=C(C=NN5C(C)C)C5=C4)=C(C)C=N3	−9.26	−9.76	−9.76
C2111	N1(CC2=CC=C(C=C2)NC3=NC(C4=CN=C(C=NN5C6CC6)C5=C4)=CC=N3)CCNCC1	−9.86	−9.75	−9.86
C2112	CN1CCN(CC1)CC2=CC=C(C=C2)NC3=NC(C4=CN=C(C=NN5C6CC6)C5=C4)=CC=N3	−9.30	−9.48	−9.48
C2113	CC(C)N1CCN(CC1)CC2=CC=C(C=C2)NC3=NC(C4=CN=C(C=NN5C6CC6)C5=C4)=CC=N3	−9.24	−9.45	−9.45
C2121	FC(C=C(C=C1)NC2=NC(C3=CN=C(C=NN4C5CC5)C4=C3)=CC=N2)=C1CN6CCNCC6	−10.06	−10.26	−10.26
C2122	FC(C=C(C=C1)NC2=NC(C3=CN=C(C=NN4C5CC5)C4=C3)=CC=N2)=C1CN6CCN(C)CC6	−9.23	−9.37	−9.37
C2123	CC(C)N1CCN(CC1)CC2=C(F)C=C(C=C2)NC3=NC(C4=CN=C(C=NN5C6CC6)C5=C4)=CC=N3	−8.96	−9.40	−9.40
C2131	COC(C=C(C=C1)NC2=NC(C3=CN=C(C=NN4C5CC5)C4=C3)=CC=N2)=C1CN6CCNCC6	−9.48	−10.04	−10.04
C2132	CN1CCN(CC1)CC2=C(OC)C=C(C=C2)NC3=NC(C4=CN=C(C=NN5C6CC6)C5=C4)=CC=N3	−9.18	−9.34	−9.34
C2133	CC(C)N1CCN(CC1)CC2=C(OC)C=C(C=C2)NC3=NC(C4=CN=C(C=NN5C6CC6)C5=C4)=CC=N3	−8.93	−9.04	−9.04
C2211	FC(C=N1)=C(C2=CN=C(C=NN3C4CC4)C3=C2)N=C1NC(C=C5)=CC=C5CN6CCNCC6	−9.74	−10.06	−10.06
C2212	CN1CCN(CC1)CC2=CC=C(C=C2)NC3=NC(C4=CN=C(C=NN5C6CC6)C5=C4)=C(C=N3)F	−9.14	−9.17	−9.17
C2213	CC(C)N1CCN(CC1)CC2=CC=C(C=C2)NC3=NC(C4=CN=C(C=NN5C6CC6)C5=C4)=C(C=N3)F	−9.05	−9.23	−9.23
C2221	FC(C=N1)=C(C2=CN=C(C=NN3C4CC4)C3=C2)N=C1NC(C=C5)=CC(F)=C5CN6CCNCC6	−9.24	−9.72	−9.72
C2222	CN1CCN(CC1)CC2=C(F)C=C(C=C2)NC3=NC(C4=CN=C(C=NN5C6CC6)C5=C4)=C(C=N3)F	−9.00	−9.07	−9.07
C2223	CC(C)N1CCN(CC1)CC2=C(F)C=C(C=C2)NC3=NC(C4=CN=C(C=NN5C6CC6)C5=C4)=C(C=N3)F	−9.02	−9.21	−9.21
C2231	FC(C=N1)=C(C2=CN=C(C=NN3C4CC4)C3=C2)N=C1NC(C=C5)=CC(OC)=C5CN6CCNCC6	−9.71	−9.59	−9.71
C2232	CN1CCN(CC1)CC2=C(OC)C=C(C=C2)NC3=NC(C4=CN=C(C=NN5C6CC6)C5=C4)=C(C=N3)F	−9.02	−9.03	−9.03

C2233	CC(C)N1CCN(CC1)CC2=C(OC)C=C(C=C2)NC3=NC(C4=CN=C(C=NN5C6CC6)C5=C4)=C(C=N3)F	−8.98	−8.95	−8.98
C2311	CC(C=N1)=C(C2=CN=C(C=NN3C4CC4)C3=C2)N=C1NC(C=C5)=CC=C5CN6CCNCC6	−9.92	−10.34	−10.34
C2312	CN1CCN(CC1)CC2=CC=C(C=C2)NC3=NC(C4=CN=C(C=NN5C6CC6)C5=C4)=C(C)C=N3	−9.64	−9.74	−9.74
C2313	CC(C)N1CCN(CC1)CC2=CC=C(C=C2)NC3=NC(C4=CN=C(C=NN5C6CC6)C5=C4)=C(C)C=N3	−9.47	−9.77	−9.77
C2321	CC(C=N1)=C(C2=CN=C(C=NN3C4CC4)C3=C2)N=C1NC(C=C5)=CC(F)=C5CN6CCNCC6	−9.72	−10.29	−10.29
C2322	CN1CCN(CC1)CC2=C(F)C=C(C=C2)NC3=NC(C4=CN=C(C=NN5C6CC6)C5=C4)=C(C)C=N3	−9.79	−9.71	−9.79
C2323	CC(C)N1CCN(CC1)CC2=C(F)C=C(C=C2)NC3=NC(C4=CN=C(C=NN5C6CC6)C5=C4)=C(C)C=N3	−9.49	−9.83	−9.83
C2331	CC(C=N1)=C(C2=CN=C(C=NN3C4CC4)C3=C2)N=C1NC(C=C5)=CC(OC)=C5CN6CCNCC6	−10.43	−9.84	−10.43
C2332	CN1CCN(CC1)CC2=C(OC)C=C(C=C2)NC3=NC(C4=CN=C(C=NN5C6CC6)C5=C4)=C(C)C=N3	−9.67	−9.71	−9.71
C2333	CC(C)N1CCN(CC1)CC2=C(OC)C=C(C=C2)NC3=NC(C4=CN=C(C=NN5C6CC6)C5=C4)=C(C)C=N3	−9.41	−9.58	−9.58
C3111	N1(CC2=CC=C(C=C2)NC3=NC(C4=CN=C(C=NN5C6CCCC6)C5=C4)=CC=N3)CCNCC1	−10.41	−10.45	−10.45
C3112	CN1CCN(CC1)CC2=CC=C(C=C2)NC3=NC(C4=CN=C(C=NN5C6CCCC6)C5=C4)=CC=N3	−10.00	−9.92	−10.00
C3113	CC(C)N1CCN(CC1)CC2=CC=C(C=C2)NC3=NC(C4=CN=C(C=NN5C6CCCC6)C5=C4)=CC=N3	−10.02	−9.98	−10.02
C3121	FC(C=C(C=C1)NC2=NC(C3=CN=C(C=NN4C5CCCC5)C4=C3)=CC=N2)=C1CN6CCNCC6	−10.70	−10.88	−10.88
C3122	FC(C=C(C=C1)NC2=NC(C3=CN=C(C=NN4C5CCCC5)C4=C3)=CC=N2)=C1CN6CCN(C)CC6	−10.14	−10.17	−10.17
C3123	CC(C)N1CCN(CC1)CC2=C(F)C=C(C=C2)NC3=NC(C4=CN=C(C=NN5C6CCCC6)C5=C4)=CC=N3	−9.88	−9.68	−9.88
C3131	COC(C=C(C=C1)NC2=NC(C3=CN=C(C=NN4C5CCCC5)C4=C3)=CC=N2)=C1CN6CCNCC6	−10.53	−10.45	−10.53
C3132	CN1CCN(CC1)CC2=C(OC)C=C(C=C2)NC3=NC(C4=CN=C(C=NN5C6CCCC6)C5=C4)=CC=N3	−9.93	−9.88	−9.93
C3133	CC(C)N1CCN(CC1)CC2=C(OC)C=C(C=C2)NC3=NC(C4=CN=C(C=NN5C6CCCC6)C5=C4)=CC=N3	−9.81	−9.82	−9.82
C3211	FC(C=N1)=C(C2=CN=C(C=NN3C4CCCC4)C3=C2)N=C1NC(C=C5)=CC=C5CN6CCNCC6	−10.73	−10.73	−10.73
C3212	CN1CCN(CC1)CC2=CC=C(C=C2)NC3=NC(C4=CN=C(C=NN5C6CCCC6)C5=C4)=C(C=N3)F	−10.01	−9.59	−10.01
C3213	CC(C)N1CCN(CC1)CC2=CC=C(C=C2)NC3=NC(C4=CN=C(C=NN5C6CCCC6)C5=C4)=C(C=N3)F	−9.56	−9.93	−9.93
C3221	FC(C=N1)=C(C2=CN=C(C=NN3C4CCCC4)C3=C2)N=C1NC(C=C5)=CC(F)=C5CN6CCNCC6	−10.20	−10.52	−10.52
C3222	CN1CCN(CC1)CC2=C(F)C=C(C=C2)NC3=NC(C4=CN=C(C=NN5C6CCCC6)C5=C4)=C(C=N3)F	−9.72	−9.88	−9.88

C3223	CC(C)N1CCN(CC1)CC2=C(F)C=C(C=C2)NC3=NC(C4=CN=C(C=NN5C6CCCC6)C5=C4)=C(C=N3)F	−9.59	−9.47	−9.59
C3231	FC(C=N1)=C(C2=CN=C(C=NN3C4CCCC4)C3=C2)N=C1NC(C=C5)=CC(OC)=C5CN6CCNCC6	−10.52	−10.77	−10.77
C3232	CN1CCN(CC1)CC2=C(OC)C=C(C=C2)NC3=NC(C4=CN=C(C=NN5C6CCCC6)C5=C4)=C(C=N3)F	−9.61	−9.52	−9.61
C3233	CC(C)N1CCN(CC1)CC2=C(OC)C=C(C=C2)NC3=NC(C4=CN=C(C=NN5C6CCCC6)C5=C4)=C(C=N3)F	−9.80	−9.68	−9.80
C3311	CC(C=N1)=C(C2=CN=C(C=NN3C4CCCC4)C3=C2)N=C1NC(C=C5)=CC=C5CN6CCNCC6	−10.73	−11.25	−11.25
C3312	CN1CCN(CC1)CC2=CC=C(C=C2)NC3=NC(C4=CN=C(C=NN5C6CCCC6)C5=C4)=C(C)C=N3	−10.17	−10.31	−10.31
C3313	CC(C)N1CCN(CC1)CC2=CC=C(C=C2)NC3=NC(C4=CN=C(C=NN5C6CCCC6)C5=C4)=C(C)C=N3	−9.85	−10.2	−10.20
C3321	CC(C=N1)=C(C2=CN=C(C=NN3C4CCCC4)C3=C2)N=C1NC(C=C5)=CC(F)=C5CN6CCNCC6	−10.35	−10.78	−10.78
C3322	CN1CCN(CC1)CC2=C(F)C=C(C=C2)NC3=NC(C4=CN=C(C=NN5C6CCCC6)C5=C4)=C(C)C=N3	−10.13	−10.3	−10.30
C3323	CC(C)N1CCN(CC1)CC2=C(F)C=C(C=C2)NC3=NC(C4=CN=C(C=NN5C6CCCC6)C5=C4)=C(C)C=N3	−10.01	−10.29	−10.29
C3331	CC(C=N1)=C(C2=CN=C(C=NN3C4CCCC4)C3=C2)N=C1NC(C=C5)=CC(OC)=C5CN6CCNCC6	−10.48	−10.69	−10.69
C3332	CN1CCN(CC1)CC2=C(OC)C=C(C=C2)NC3=NC(C4=CN=C(C=NN5C6CCCC6)C5=C4)=C(C)C=N3	−10.10	−10.08	−10.10
C3333	CC(C)N1CCN(CC1)CC2=C(OC)C=C(C=C2)NC3=NC(C4=CN=C(C=NN5C6CCCC6)C5=C4)=C(C)C=N3	−9.76	−10.03	−10.03

Table S9. Binding free energies ($\Delta \mathbf{G}_{\mathbf{bind}}^{\mathbf{cal}}$) for **C2213**/CDK6 system.

Energy	Complex		Receptor		Ligand		Delta	
	Average	Std. Dev.	Average	Std. Dev.	Average	Std. Dev.	Average	Std. Dev.
E_{vdW}	-2401.07	21.52	-2347.66	21.17	-4.41	1.74	-49.00	3.40
E_{ele}	-20093.82	90.96	-20114.54	90.90	35.88	2.08	-15.17	3.02
E_{GB}	-3210.87	69.78	-3231.13	69.82	-23.99	0.83	44.25	3.33
E_{surf}	103.27	1.55	104.91	1.53	3.93	0.08	-5.57	0.31
E_{gas}	-6206.30	92.47	-6054.86	92.12	-87.27	5.62	-64.17	4.29
E_{solv}	-3107.60	69.19	-3126.22	69.22	-20.06	0.80	38.68	3.17
$E_{gas} + E_{sol}$	-9313.90	50.32	-9181.08	49.85	-107.33	5.58	-25.49	2.80
TS_{total}	3282.50	8.23	3244.43	4.95	61.10	0.10	-23.03	5.63
ΔG_{bind}^{cal}							-2.46	6.29

The binding free energies (ΔG_{bind}^{cal}) for **C2213**/CDK6 complex and decomposition to electrostatic interaction (E_{ele}), van der Walls interaction (E_{vdW}), polar solvation free energies (E_{GB}), nonpolar solvation free energies (E_{surf}), and entropy (TS_{total}). Energy values are presented in kcal/mol. Uncertainties were calculated as the root mean square error for all frames extracted from the trajectories.

Table S10. Binding free energies ($\Delta\mathbf{G}_{\mathbf{bind}}^{\mathbf{cal}}$) for C2213-A/CDK6 system.

Energy	Complex		Receptor		Ligand		Delta	
	Average	Std. Dev.	Average	Std. Dev.	Average	Std. Dev.	Average	Std. Dev.
E_{vdW}	-2388.93	21.43	-2332.55	20.91	-4.16	1.54	-52.21	3.80
E_{ele}	-20053.69	99.49	-20153.31	99.81	109.56	2.03	-9.94	3.41
E_{GB}	-3226.47	76.50	-3243.58	77.20	-23.65	0.67	40.76	3.31
E_{surf}	103.40	1.77	105.43	1.68	4.28	0.03	-6.31	0.44
E_{gas}	-6219.45	96.88	-6037.87	97.41	-119.42	5.76	-62.16	4.69
E_{solv}	-3123.07	75.99	-3138.15	76.62	-19.37	0.66	34.45	3.15
$E_{gas} + E_{sol}$	-9342.51	51.37	-9176.01	50.70	-138.79	5.69	-27.71	3.65
TS_{total}	3280.16	11.34	3235.36	11.87	63.37	0.00	-18.57	6.00
ΔG_{bind}^{cal}							-9.14	7.02

The binding free energies (ΔG_{bind}^{cal}) for **C2213-A**/CDK6 complex and decomposition to electrostatic interaction (E_{ele}), van der Walls interaction (E_{vdW}), polar solvation free energies (E_{GB}), nonpolar solvation free energies (E_{surf}), and entropy (TS_{total}). Energy values are presented in kcal/mol. Uncertainties were calculated as the root mean square error for all frames extracted from the trajectories.
